# Mechanistic Insights into Molecular Crystalline Organometallic Heterogeneous Catalysis through Parahydrogen-Based Nuclear Magnetic Resonance Studies

**DOI:** 10.1021/jacs.2c12642

**Published:** 2023-01-23

**Authors:** Matthew R. Gyton, Cameron G. Royle, Simon K. Beaumont, Simon B. Duckett, Andrew S. Weller

**Affiliations:** † Department of Chemistry, University of York, York YO10 5DD, U.K.; ‡ Centre for Hyperpolarisation in Magnetic Resonance, Department of Chemistry, University of York, Heslington, York YO10 5DD, U.K.; § Department of Chemistry, University of Oxford, Mansfield Road, Oxford OX1 3TA, U.K.; ∥ Department of Chemistry, Durham University, South Road, Durham DH1 3LE, U.K.

## Abstract

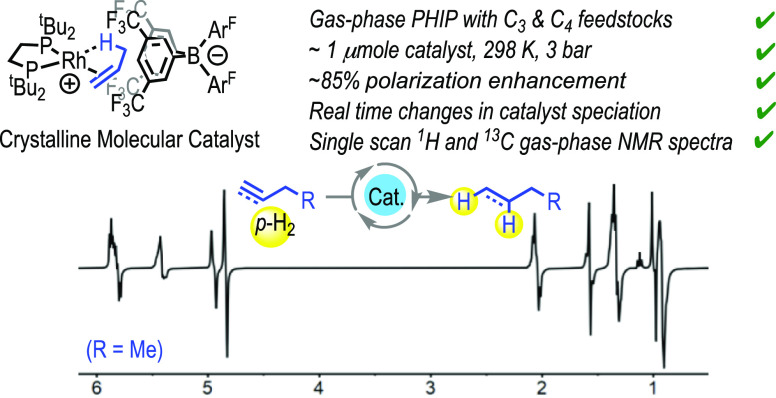

The heterogeneous solid–gas reactions of crystals of [Rh(L_2_)(propene)][BAr^F^_4_] (**1**, L_2_ = ^*t*^Bu_2_PCH_2_CH_2_P^*t*^Bu_2_) with H_2_ and propene, 1-butene, propyne, or 1-butyne are explored by gas-phase nuclear magnetic resonance (NMR) spectroscopy under batch conditions at 25 °C. The temporal evolution of the resulting parahydrogen-induced polarization (PHIP) effects measures catalytic flux and thus interrogates the efficiency of catalytic pairwise *para*-H_2_ transfer, speciation changes in the crystalline catalyst at the molecular level, and allows for high-quality single-scan ^1^H, ^13^C NMR gas-phase spectra for the products to be obtained, as well as 2D-measurements. Complex **1** reacts with H_2_ to form dimeric [Rh(L_2_)(H)(μ-H)]_2_[BAr^F^_4_]_2_ (**4**), as probed using EXAFS; meanwhile, a single-crystal of **1** equilibrates NMR silent *para*-H_2_ with its NMR active ortho isomer, contemporaneously converting into **4**, and **1** and **4** each convert *para*-H_2_ into *ortho*-H_2_ at different rates. Hydrogenation of propene using **1** and *para*-H_2_ results in very high initial polarization levels in propane (>85%). Strong PHIP was also detected in the hydrogenation products of 1-butene, propyne, and 1-butyne. With propyne, a competing cyclotrimerization deactivation process occurs to afford [Rh(^*t*^Bu_2_PCH_2_CH_2_P^*t*^Bu_2_)(1,3,4-Me_3_C_6_H_3_)][BAr^F^_4_], while with 1-butyne, rapid isomerization of 1-butyne occurs to give a butadiene complex, which then reacts with H_2_ more slowly to form catalytically active **4**. Surprisingly, the high PHIP hydrogenation efficiencies allow hyperpolarization effects to be seen when H_2_ is taken directly from a regular cylinder at 25 °C. Finally, changing the chelating phosphine to Cy_2_PCH_2_CH_2_PCy_2_ results in initial high polarization efficiencies for propene hydrogenation, but rapid quenching of the catalyst competes to form the zwitterion [Rh(Cy_2_PCH_2_CH_2_PCy_2_){η^6^-(CF_3_)_2_(C_6_H_3_)}BAr^F^_3_].

## 1 Introduction

Catalytic processes are often conveniently divided into homogeneous or heterogeneous, and while both are important, industrial catalysis often operates using the latter due to the benefits associated with catalyst stability, the physical separation of catalyst and substrates/products, operation in flow, and recyclability.^1−3^ Central to optimizing both types of catalysis, though, is the ability to define and control the catalytically active site(s) through the determination of structure–activity relationships, and attenuation of deactivation processes.^4−6^ Compared with the atomic-level precision that homogeneous systems provide in both the synthesis and interrogation of active sites, heterogeneous catalysts are arguably more challenging to characterize and manipulate due to the complex and diverse manifold of active surface sites, which are often also only present in low abundance. This challenge is amplified under operando conditions where catalyst reconstruction can lead to changes in catalyst performance.^7^ Elegant solutions to controlling, and enhancing, activity in heterogeneous catalysis often comes at the nexus of molecular and extended solids through single atom catalysis,^8−10^ surface organometallic chemistry (SOMC),^11,12^ ligand coordinated single atom catalysts,^13^ or catalysts supported in mesoporous framework materials.^14^

In this report, we show that gas-phase NMR analysis can deliver real-time insights into changes in catalyst speciation of a solid-state molecular organometallic (SMOM^15^) heterogeneous catalyst. As NMR spectroscopy is inherently insensitive, we use gas-phase *para*-hydrogen (*p*-H_2_)-induced polarization (PHIP^3^) to achieve this outcome by correlating molecular level changes to the catalyst with both product identity and flux in the, industrially important,^16^ catalytic solid/gas hydrogenation of unsaturated C_3_ (propene, propyne) and C_4_ (butene/butyne) substrates at 298 K.

*Para*-H_2_ is the NMR silent nuclear-spin isomer of H_2_ that is thermodynamically preferred over *ortho*-H_2_ because of the 120 cm^–1^ separation between their rotational levels. At room temperature, H_2_ exists in an approximate 3:1 ratio of ortho to para spin isomers.^17^ This can be enhanced to ∼100% *para*-H_2_ by cooling over a catalyst to approximately −250 °C. The benefit of using *para*-enriched H_2_ in hydrogenation reactions comes from the non-Boltzmann spin distributions that exceed those normally available to NMR through the Zeeman effect.^18^ These arise when pairwise addition of *para*-H_2_ occurs to a reactant with retention of spin correlation, such that significant signal enhancements in resulting NMR spectra result, of up to 31,000 fold in resulting NMR spectra recorded on a 400 MHz spectrometer.^19^ This signal gain aids in the detection of both products and, low concentration, intermediate species in a catalytic cycle (the latter normally in the solution state^20,21^). In principle weak PHIP should also be observed using normal H_2_ at room temperature, but the low retention of hyperpolarization in the resulting products means that such an enhancement is very rarely observed.^22,23^ 100% enriched *para*-H_2_ is thus routinely used—with the attendant requirement for specialized equipment.

The development of PHIP methods for generating hyperpolarized propane, by propene hydrogenation, is also of significant interest in human lung imaging,^24^ reactor and microfluidic device visulation,^25−27^ and high-resolution MRI detection.^28^ Here, heterogeneous catalysts operating at high temperature have been extensively used to generate hyperpolarized propane, often under experimentally challenging flow conditions,^29−31^ for example, supported heterogeneous catalysts that operate at 100 °C or above. However relatively weak polarizations normally result (∼3 to 11%)^32−34^ and catalyst efficiencies normally remain low (∼10%),^35−38^ which combined lead to low overall catalytic flux. As propane’s ^1^H and ^13^C *T*_1_ relaxation times are short, rapid and efficient creation of a high flux of hyperpolarized product is critical if operando catalytic methods or imagining applications using PHIP are to be developed.^25,33^

**Scheme 1:**
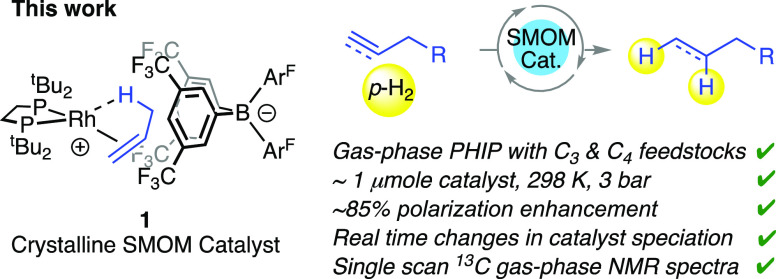
This Work [Ar^F^ = (3,5-CF_3_)_2_C_6_H_3_], R = H, Me

In this contribution, we show that by using the straightforward to prepare SMOM catalyst, [Rh(^*t*^Bu_2_PCH_2_CH_2_P^*t*^Bu_2_)(propene)][BAr^F^_4_], **1** [Ar^F^ = 3,5-(CF_3_)_2_C_6_H_3_], Scheme 1, a number of important observations can be made, which revolve around the high catalytic flux observed in solid/gas *para*-hydrogenation reactions of C_3_ (propene and propyne) and C_4_ (butene and butyne) substrates that this catalyst promotes. In addition, we show that molecular-level changes to the catalyst structure in the solid-state can be signaled by temporal changes to this catalytic flux.

(i) Changes in catalyst speciation in the molecular solid-state can be measured by the flux of *para*-H_2_ to *ortho*-H_2_ conversion.
(ii) High levels of polarization enhancement (up to 85%) for C_3_ and C_4_ substrate hydrogenation occur at 25 °C, that produces a significant flux of hyperpolarized product, allowing for detailed mechanistic insights into the catalytic manifold.
(iii) High-quality single-scan gas phase ^13^C{^1^H} NMR spectra, and rapid 2D ^1^H–^1^H COSY and ^1^H–^13^C HMQC measurements of products are possible.
(iv) The high polarization enhancements allow for *para*-H_2_ use direct from a normal cylinder, enabling PHIP measurements without the need for specialist equipment.

## 2 Results and Discussion

### 2.1 Synthesis of Complex **1** and Solid/Gas Reactivity with H_2_ and Propene: The Formation of a Hydride-Bridged Dimer in the Solid-State

**Scheme 2:**
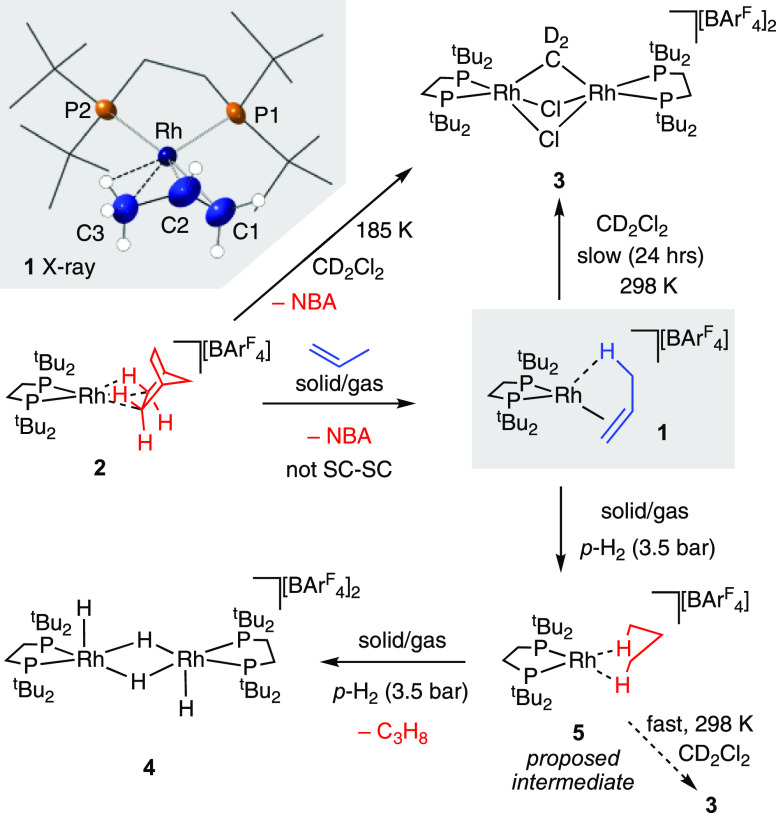
Synthesis and Reactivity of Complex **1** with H_2_

Complex **1** is synthesized by the solid/gas reaction of propene with the previously reported σ-alkane complex^39^ [Rh(^*t*^Bu_2_PCH_2_CH_2_P^*t*^Bu_2_)(NBA)][BAr^F^_4_], **2** (NBA = norbornane), Scheme 2, that is itself generated by solid/gas hydrogenation of a norbornadiene (NBD) precursor^40^ that can be prepared on ∼5 g scale. Long-range order is not retained for the formation of **1**, as there is a loss of significant Bragg peaks by X-ray diffraction, but low temperature (−98 °C) ^31^P{^1^H} solid-state NMR (SSNMR) spectroscopy shows relatively sharp peaks centered at δ 114.9 and 118.9, consistent with the retention of short range order.^41,42^ This likely reflects a well-defined organometallic cation sitting inside a cage of [BAr^F^_4_]^−^ anions that have randomly disordered aryl groups.^39^ While complex **1** is stable in the solid-state at 25 °C for months under Ar, it decomposes slowly over 24 h in CD_2_Cl_2_ solution at 25 °C to form the known solvent-activated dimer [Rh_2_(^*t*^Bu_2_PCH_2_CH_2_P^*t*^Bu_2_)_2_(μ-CD_2_)(μ-Cl)_2_][BAr^F^_4_]_2_, **3**.^39^ At 25 °C, signals due to bound propene are not observed in the ^1^H NMR spectrum (CD_2_Cl_2_) of complex **1**, while a single environment is observed in the ^31^P{^1^H} NMR spectrum, suggesting a fluxional process is occurring: likely a 1,3-hydride shift that reflects a degenerate isomerization.^15^ At −80 °C, the ^1^H and ^31^P{^1^H} NMR data support a static structure for **1** that has a single propene ligand bound with a supporting CH_3_···Rh agostic interaction, for example: δ(^1^H): −0.64, 3H; δ(^31^P): 112.3, *J*(RhP) = 162 Hz; 116.4 *J*(RhP) = 211 Hz. This is confirmed by a solid-state structure of **1** as recrystallized from solution (hexane/CH_2_Cl_2_) under an atmosphere of propene.^43^

Reaction of finely crushed **1** with *para*-H_2_ under standardized conditions (3.5 bar, 298 K, 2 mg, sealed NMR tube, 2 min) and interrogation of the resulting dissolved solid (i.e., after full relaxation of hyperpolarized nuclear spin isomers) by ^1^H and ^31^P{^1^H} NMR spectroscopies (CD_2_Cl_2_, 298 K) showed the complete consumption of **1** and the formation of the previously reported^39^ Rh(III) hydride bridged dimer [Rh(^*t*^Bu_2_PCH_2_CH_2_P^*t*^Bu_2_)H(μ-H)]_2_[BAr^F^_4_]_2_, **4**, alongside a small amount of complex **3**, in a 80:20 ratio, respectively. Propane is also observed by gas-phase NMR spectroscopy. We speculate that **3** forms from a reaction of the proposed, but not directly observed, σ-propane intermediate [Rh(^*t*^Bu_2_PCH_2_CH_2_P^*t*^Bu_2_)(C_3_H_8_)][BAr^F^_4_] **5** with H_2_, that itself is formed on initial hydrogenation of **1**. In support of this assignment, the very close analogue of **5**, crystallographically characterized, but short-lived at 25 °C, propane σ-complex [Rh(Cy_2_PCH_2_CH_2_PCy_2_)(C_3_H_8_)][BAr^F^_4_], has been prepared by solid/gas hydrogenation,^44^ while the more stable σ-alkane complex **2** reacts rapidly in CD_2_Cl_2_ to also give complex **3** (Scheme 2).^39^ In situ SSNMR experiments on bulk crystalline material (∼60 mg **1**, H_2_ addition for 2 min) showed the formation of **4** (br, δ 124.0), and small amount (∼10%) of a complex with a characteristically downfield shifted signal^44^ at δ 128 that is assigned to **5** in comparison with **2** (δ 126).^39^ A separate sample of **1** taken through five successive H_2_ cycles demonstrated complete conversion of **1** into **4** (both by solution and SSNMR experiments), while the addition of H_2_ to **1** for only ∼10 s results in a reversed ratio of **4**:**3** of 20:80. Overall these observations and data show that complex **4** forms from H_2_ addition to **1**, and is suggested to proceed via a σ-propane complex **5** for which complex **3** can be used as an indirect spectroscopic marker. Addition of *para*-H_2_ to single monolithic crystals of complex **1** (0.4 mm × 0.4 mm × 0.4 mm) (∼0.05 mg, 2 min) results in the same speciation to the detection limit of ^31^P{^1^H} NMR spectroscopy.

**Figure 1:**
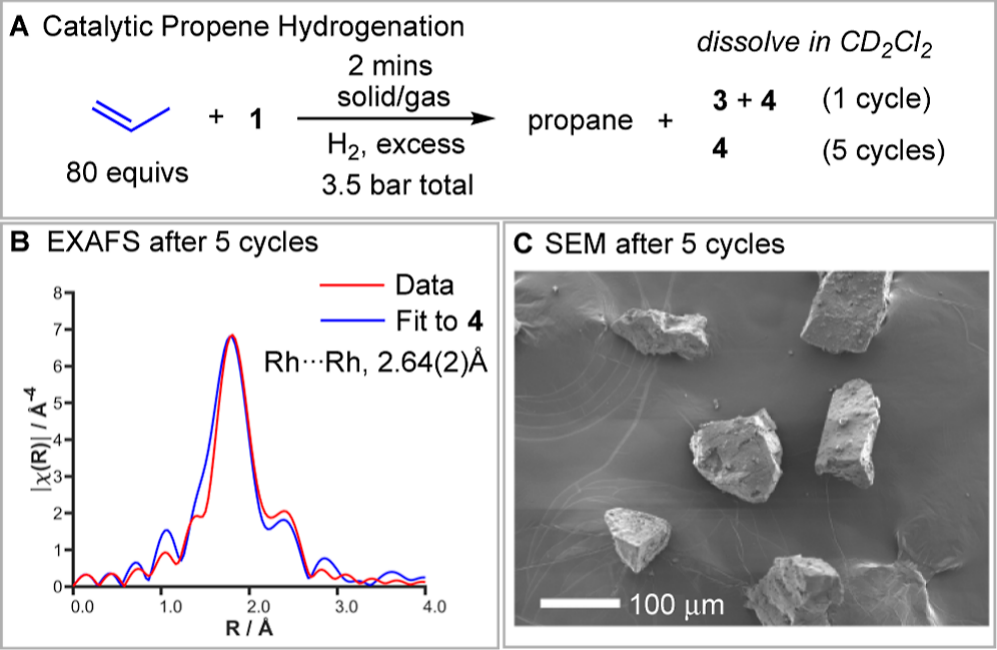
(A) Products observed during the catalytic solid/gas propene hydrogenation by finely crushed **1** (2 mg); (B) Rh K-edge EXAFS data and near neighbor fit (1.0–3.2 Å) after 5 H_2_/propene cycles (fitting details given in the Supporting Information) (C) SEM images of post-catalysis materials (after 5 H_2_/propene cycles).

The catalytic solid/gas hydrogenation of propene under standardized conditions using complex **1** (3.5 bar absolute, H_2_/propene ∼2:1, 298 K, 2 mg of finely crushed crystals) occurs rapidly as probed by gas-phase ^1^H NMR spectroscopy (100% conversion in 2 min, unoptimized, TON_app_^45^ = 80), Figure 1A. Dissolved material, post catalysis (CD_2_Cl_2_, 25 °C) after 1 H_2_ addition cycle shows a mixture of **3**, **4** in a 20:45 ratio, the former arising from σ-alkane complex **5** before it undergoes the onward reaction with H_2_ to form **4**. The mass balance of 35% is taken up with unidentified hydride-containing species (Figure S16). However, five cycles of H_2_/propene results in the formation of **4** alone, with no **3** observed. Independently synthesized **4** also mediates the solid/gas hydrogenation of propene under standardized conditions, but more slowly (2 min, TON_app_ = 65). To determine whether the hydride bridged dimer **4** is generated directly in the solid/state, or from quenching of a reactive {Rh(L_2_)H_2_}^+^ monomer on dissolving in CD_2_Cl_2_, Rh K-edge EXAFS experiments were performed in samples of **1** that had mediated five cycles of propene hydrogenation, Figure 1B. These show the direct formation of a dimeric species in the solid-state, similar to that reported for the Ir-congener.^46^ The formation of similar hydride bridged dimers in the solid-state has been reported on hydrogenation of [Ir(triphos)H_2_(ethene)][BPh_4_] at 70 °C,^47^ while other solid-state monomer/dimer transformations are known in organometallic chemistry.^48,49^ The Rh–Rh distance of 2.64(2) Å compares favorably with independently synthesized **4**: EXAFS, 2.66(2) Å and single-crystal X-ray diffraction, 2.6575(5) Å.^39^ Inclusion of Rh–Rh bonding was essential to fit the obtained data, confirming the dimeric nature of the solid.^50^ The ^31^P{^1^H} SSNMR spectrum of complex **4** formed in this way after catalysis shows relatively sharp signals, suggesting the retention of short-range order, while SEM analysis shows intact, but significantly fractured, crystalline material, Figure 1C (Figure S39 shows SEM of the starting complex **1**). No evidence for [BAr^F^_4_]^−^ coordination at a Rh(I) center is observed, which is different from [Rh(Cy_2_PCH_2_CH_2_PCy_2_)][BAr^F^_4_] SMOM systems which form such zwitterions on decomposition (vide infra).^40,44^ This is likely a consequence of the increased steric profile of the ^*t*^Bu groups, coupled with the relative accessibility of a Rh(III) dihydride.^51^ Collectively these observations suggest that σ-alkane complex **5** is a more efficient propene hydrogenation catalyst than in situ formed dimeric **4**.

### 2.2 Reaction of Complex **1** with C_3_ and C_4_ Alkenes and Alkynes: Substitution, Cyclotrimerization, and Isomerization

**Scheme 3:**
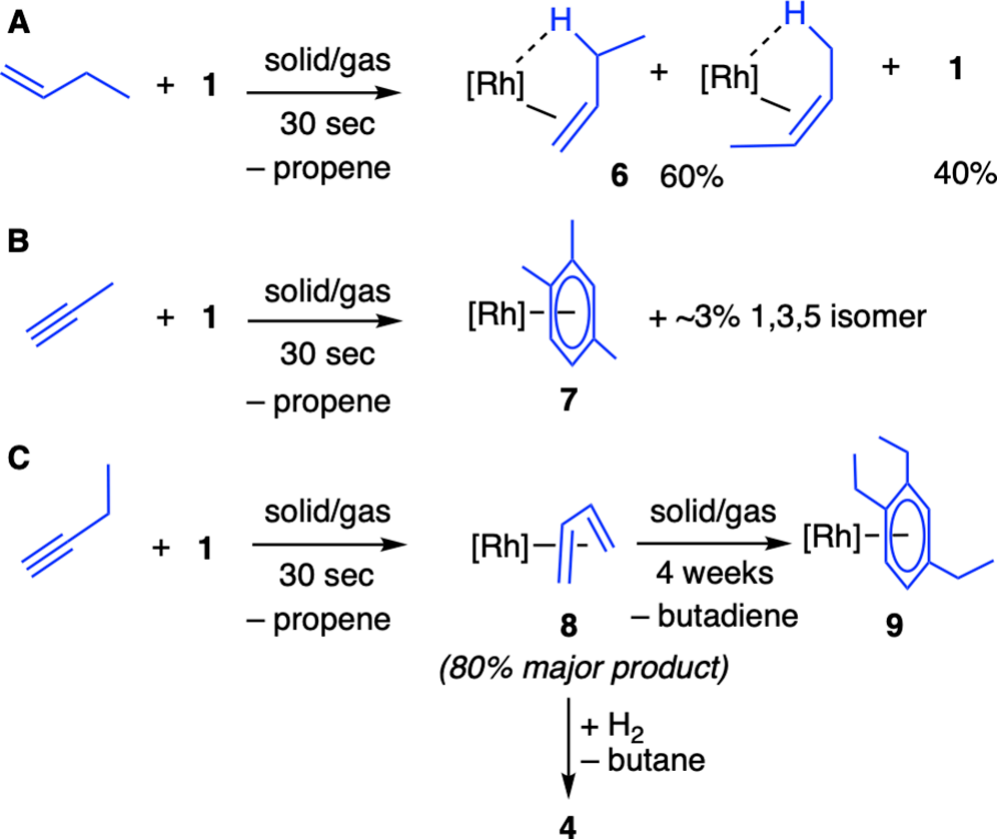
Reaction of **1** with 1-Butene, Propyne, and 1-Butyne [Rh] = [Rh(^*t*^Bu_2_PCH_2_CH_2_P^*t*^Bu_2_)][BAr^F^_4_].

To further baseline the reactivity of **1** before *para*-H_2_ experiments are discussed, the stoichiometric reactivity with 1-butene, propyne, and 1-butyne is presented, Scheme 3.

The solid/gas reaction with butene (1 bar, 30 s) results in the formation of a mixture of **1** and previously reported [Rh(^*t*^Bu_2_PCH_2_CH_2_P^*t*^Bu_2_)(butenes)][BAr^F^_4_], **6**, in a 40:60 ratio, respectively. The solid/gas reaction with propyne results in the rapid (1 bar, 30 sec) alkyne cyclotrimerization^52^ to form trimethylbenzene-bound [Rh(^*t*^Bu_2_PCH_2_CH_2_P^*t*^Bu_2_)(1,2,4-Me_3_C_6_H_3_)][BAr^F^_4_], **7**, that was characterized by solution state NMR spectroscopy,^53^ and compared with independently, solution-synthesized, material (Supporting Information, Materials). A small amount (∼3%) of the 1,3,5-isomer is formed in both reactions, demonstrating that there is no reaction pathway bias in the solid-state reaction compared with solution. Free propene is also observed by gas phase ^1^H NMR spectroscopy as a very minor component. Isolated **7**, with its relatively strongly bound arene, is a very slow propene hydrogenation catalyst (hours) in the solid-state, also being returned unchanged at the end of catalysis. Supressed hydrogenation activity by arene coordination is well-established in homogeneous catalysis using [Rh(chelating-diphosphine)]^+^ systems,^54^ and here we extended this into SMOM chemistry.

In contrast, the solid/gas reaction of complex **1** with 1-butyne (1 bar, 30 sec) results in the formation of the known^39^ butadiene complex [Rh(^*t*^Bu_2_PCH_2_CH_2_P^*t*^Bu_2_) (butadiene)][BAr^F^_4_], **8**, as the main product (∼80%)^55^ alongside Rh(I) species tentatively identified as enyne-containing products,^56^ and free propene. This ensemble reacts with H_2_ in a solid/gas reaction to form hydride bridged dimer, **4**, butane and a mixture of linear and branched C_8_-hydrocarbons. In the solid-state, the cyclotrimerization product [Rh(^*t*^Bu_2_PCH_2_CH_2_P^*t*^Bu_2_)(1,2,4-Et_3_C_6_H_3_)][BAr^F^_4_], **9**, now forms considerably slower (4 weeks) through the release of butadiene. We propose this attenuated reactivity is due—in large part—to the relatively strongly bound butadiene ligand, as previously noted.^39^ We propose that the initially formed butadiene complex **8** is formed from **1** by the selective isomerization of butyne. While rare, such isomerization is reported over metal oxides, such as CaO, at relatively low temperatures.^57^

Collectively these observations demonstrate that complex **1** undergoes a rich reaction chemistry in the solid-state. With these results in hand experiments using *para*-H_2_ as probe for catalyst identity and restructuring are now described and interpreted.

### 2.3 Assessing Catalyst Reconstruction through *para*-H_2_ to *ortho*-H_2_ Conversion

**Figure 2:**
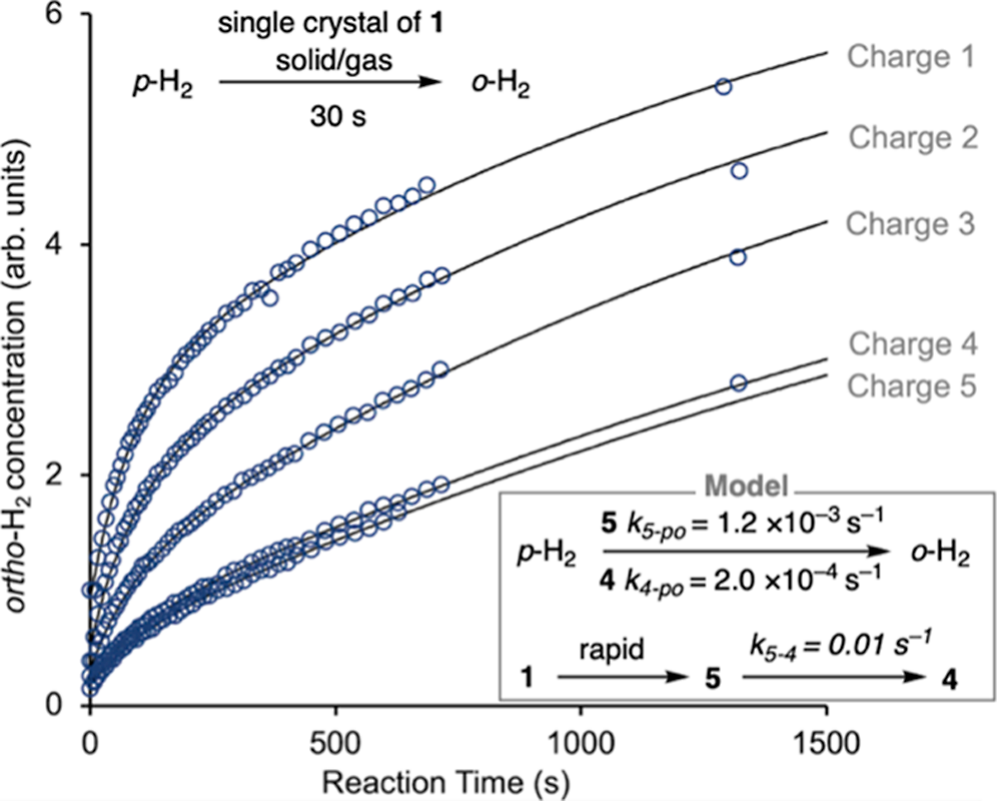
Growth in ^1^H NMR signal for *ortho*-H_2_ as a function of reaction time starting with **1**. Experimental data (circles), simulated result (line) as a function of recharge. Inset shows the model used and derived rate constants.

We started our catalytic investigations by looking for a reversible interaction between crystalline **1** and NMR silent *para*-H_2_, which would be expected to produce NMR-visible *ortho*-H_2_. Informed by our previous experiments, this would occur via rapidly formed **5** or **4**, which we hypothesized may convert *para*-H_2_ into *ortho*-H_2_ at different rates. A 5 mm NMR tube containing a single crystal of **1** (∼0.05 mg) was charged with 3.5 bar of pure *para*-H_2_ at low field prior to immediately recording a gas phase ^1^H NMR spectrum at high field. The growth of *ortho*-H_2_ in the head space of the NMR tube was monitored as a function of time over a total of five reaction re-charges. Analysis of the resulting signal intensity data showed that the growth curve did not fit to a simple exponential, while the absolute growth rate fell with each subsequent recharge. Informed by the earlier speciation observations, a simple model was developed where **1** converts rapidly into **5**, which catalyzes *para*–*ortho* H_2_ exchange (*k*_5-po_ = 1.2 × 10^–3^ s^–1^) while also converting into **4** (*k*_5-4_ = of 0.01 s^–1^), which itself is a *para*–*ortho* H_2_ exchange catalyst (*k*_4-po_ = 2 × 10^–4^ s^–1^), Figure 2. Support for this model comes from using similarly sized crystals of isolated **4**, from which the corresponding *ortho*-H_2_ signal changes fit to the same *k*_**4**-po_ value as determined from the ensemble.^58^ These data show that dihydride **4** is a slower *para*-H_2_ conversion catalyst than σ-propane complex **5**, and, importantly, demonstrate that the catalyst reconstruction in the solid-state can be reported upon through *para*–*ortho* H_2_ conversion dynamics. Furthermore, such speciation changes can also be evidenced through studies on the hydrogenation of simple alkenes and alkynes using *para*-H_2_, as discussed next.^59^

### 2.4 PHIP of C_3_ and C_4_ Substrates: Enhancements through Strong Catalytic Flux

**Scheme 4:**
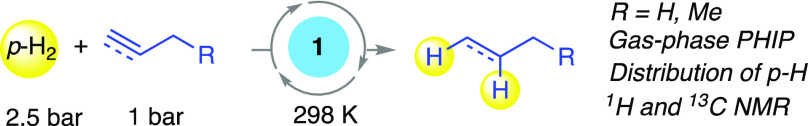
Gas-Phase PHIP with C_3_ and C_4_ Substrates

With the speciation changes in the solid-state demonstrated under conditions of either H_2_ or alkene/alkyne addition, the catalytic hydrogenation of these substrates by *para*-H_2_ was next investigated, Scheme 4. To do this, 2 mg samples of catalyst **1** were examined over up to five hydrogenation cycles. Our standard procedure was to sequentially add the appropriate substrate followed by *para*-H_2_, in a 1:2.5 ratio (3.5 bar total, ∼30 s between additions), external to the magnet in low-field, before quickly (∼10 s) transferring to high-field for measurement. This gives the opportunity for hyperpolarization transfer by *para*-H_2_ addition in both low-field in the first scan (ALTADENA conditions) and high field in subsequent scans (PASADENA).^60,61^ These situations are readily distinguished by the appearance of the enhanced NMR signals which vary according to the contribution level of pairs of doublets of opposite-phase to pairs of antiphase doublets, respectively.^62^

#### 2.4.1 Propene: 85% Polarization Transfer and Single-Scan Gas Phase ^13^C NMR

**Figure 3:**
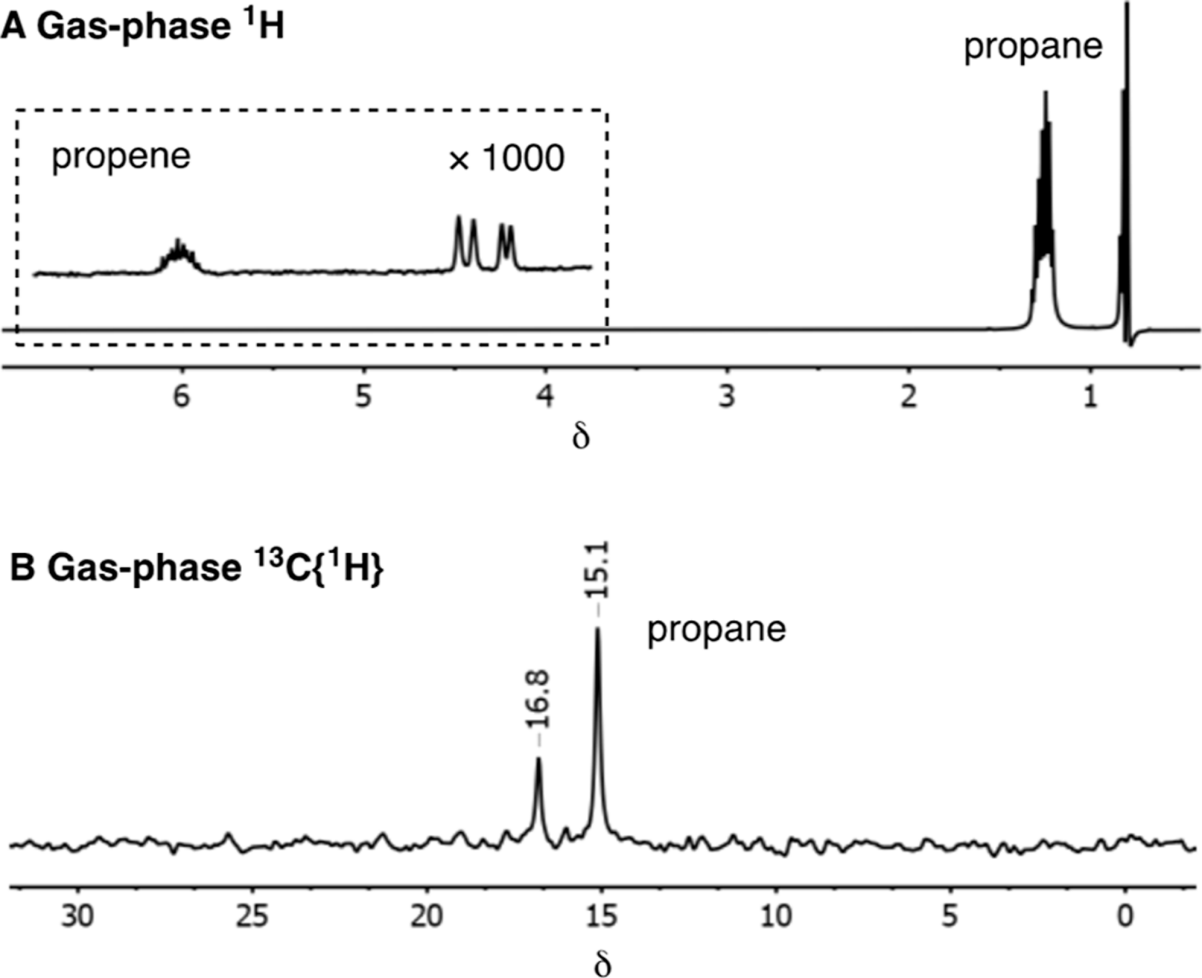
Single-scan (A) ^1^H NMR spectrum of propene hydrogenation showing the scale of PHIP signal enhancement in propane observed when **1** reacts a 3.5 bar of a 1:2.5 mixture of propene and *para*-H_2_, inset shows propene signal relatively scaled × 1000. This initial spectrum shows the effects of radiation damping due to the exceptionally large signal gains, which results in the distortion of the normal ALTADENA type behavior. (B) Equivalent single-scan ^13^C-INEPT spectrum of the propane formed under these conditions.

Using propene as the substrate, ALTADENA-type signals are observed for propane at 1.37 (CH_2_) and 0.90 (CH_3_) ppm at the start of each charging cycle due to hydrogenation occurring at low field, alongside little, if any, free *ortho*-H_2_ signal. The latter observation suggests that alkene coordination attenuates reversible H_2_ addition. Remarkably, the propane’s CH_3_ signal in the gas-phase NMR spectrum was ∼1750 times more intense than that of the CH for unreacted propene (Figure 3A) on the first measurement. This correlates to a very high polarization efficiency of ∼85%. This ratio reduced to 1:650 for the first measurement of the second recharge cycle, and to 1:85 for the fifth cycle. While this progressively slower turnover is also reflected under PASADENA conditions by longer conversion times for 50% conversion in the first three cycles (e.g., 15, 54, and 100 s, respectively), PHIP is also observed over this extended timescale. These data collectively support a slowing of turnover frequency due to the formation of **4**, but that complex **4** remains a competent but slower hydrogenation catalyst leading to appreciable catalytic PHIP flux. Furthermore, these data are consistent with the retention of a high product hyperpolarization level despite the slowing reaction. Complex **4** is observed as the only organometallic species after multiple re-charges.

A series of sequential NMR observations were also made using the OPSY protocol,^63^ which selects signals for protons originating from *para*-H_2_ before they relax and are not detected. These thus report precisely on the lifetime over which PHIP polarization is visible for each cycle of measurements and thus reflects how catalytic flux changes with reaction progress. The initially strong propane signals of the first measurement fell to zero over ∼50 s, at which point the propene was all consumed. While subsequent recharge cycles—where complex **4** is now the active catalyst (Figure 1)—are slower, they returned OPSY signals from propane for longer time-periods, confirming a longer-lived catalyst and a more consistent catalytic flux.

One significant benefit of the strongly enhanced ^1^H NMR signals created for propane using SMOM catalyst **1** is that relatively high-quality single-scan gas-phase ^13^C INEPT spectra can be recorded in less than a second, as shown in Figure 3B. The ^13^CH_3_ and ^13^CH_2_ signals are visible at 15.1 and 16.8 ppm, with the former having a *S*/*N* value of 41:1 initially. These observations further confirm that the initial propane polarization level is very high. Recent elegant reports exploiting gas-phase PHIP in a heterogeneous hydrogenation of propene using Rh/TiO_2_ at 130 °C have also demonstrated such ^13^C detection, albeit after multiple (256) scans.^33^

#### 2.4.2 Butene

Analogous studies on the hydrogenation of 1-butene show a very similar behavior. However, while the initial measurements showed a strong ALTADENA type PHIP, the expected products (butane, and propane from hydrogenation of **1**) could not be differentiated in the ^1^H NMR spectrum due to peak overlap in the aliphatic region (vide infra). A separate initial single-scan gas-phase ^13^C-INEPT measurement detects just butane, by the observation of signals at δ 26.5 and 13.0 (Figure S75). This suggests that the amount of propane formed is relatively small, consistent with a higher catalytic flux for butene hydrogenation at, or near, the surface of the crystals compared with the stoichiometric propene hydrogenation of **1** in the bulk.

The initial intensity of the CH_3_ group on butane is ∼380 times more intense than that for the C–H group of unreacted 1-butene, indicating a lower catalytic flux for butene hydrogenation compared with propene (cf. 1750). In support of this, the corresponding PASADENA experiments show a time for 50% conversion of 1-butene of 50 s, which is longer than the corresponding 15 s for propene. This extends to 350 s in the second recharge cycle and over 650 s in the third, and is accompanied by a significant reduction in signal enhancement, consistent with a slower acting catalyst being formed, that is, **4**.^64^ However, just as for propene hydrogenation, the corresponding OPSY experiments (second recharge cycle) show that gas-phase PHIP is still visible for 37 min, supporting longer-lived, albeit slower, catalysis that sustains appreciable catalytic flux for butene hydrogenation. After multiple recharge cycles, complex **4** is the only species observed when the catalyst is dissolved in CD_2_Cl_2_. We have previously shown that butene complexes **6** react in the solid-state with H_2_ to form **4**.^39^

#### 2.4.3 Propene and Butene

Resolving mixtures of hydrogenation products using rapid PHIP-enhanced gas-phase ^13^C-INEPT and 2D ^1^H–^13^C HMQC experiments. A 1:1 mixture of propene and 1-butene hydrogenated under our standard conditions using *para*-H_2_ and catalyst **1** was used to explore if the PHIP enhancements observed individually could be harnessed to resolve the behavior of mixtures. The resulting PHIP enhanced single-scan gas-phase ^13^C-INEPT spectrum now contained strong signals for both butane (δ 26.2 and 12.8) and propane (δ 17.1 and 15.5), Figure S100. Accordingly, the overlap of the aliphatic signals observed in the ^1^H NMR spectrum of propane and butane, could now be resolved using a gas-phase 2D ^1^H–^13^C HMQC measurement, which harnesses the ^13^C signal separation (Figure S101). The total acquisition time of this experiment was less than 1 min. These observations demonstrate the suitability of this methodology for the rapid detection of individual components of mixtures in the gas-phase in addition to the more common gas-phase ^1^H NMR PHIP.^30^ It also provides mechanistic insights in that both alkenes must compete effectively for metal center coordination during hydrogenation.

#### 2.4.4 Propyne: Insights into Reaction Mechanism Using Gas-Phase PHIP and the Cyclotrimerization Deactivation Product

**Scheme 5:**
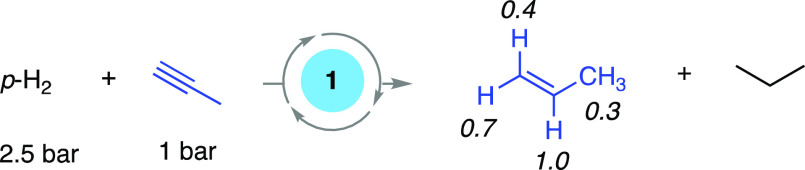
Gas-Phase PHIP with Propyne Numbers in parenthesis represent relative intensities of hyperpolarized signals in the OPSY experiments.

*Para*-hydrogenation experiments with propyne under our standard conditions revealed the formation of both propene and propane in the gas-phase, as expected for a sequential hydrogenation. In the first measurement at 25 °C, after transfer from low-field, the signals for all four of propene’s proton sites (alkene and methyl) exhibited enhancement, but that for the methyl dominated. Signals were also seen for propane, with the CH_3_ signal integral only ∼25% in intensity compared with propene methyl signal. The next measurement was associated with the detection of reaction products formed in high-field, and yielded propene signals with relative intensities of 1.0 (CHMe)/0.4 (CH_cis to Me_)/1.2 (CH_cis to H_)/0.4 (Me). The corresponding OPSY data support this ratio, with signals of 1:0.4:0.7:0.3 observed, respectively, Scheme 5. These OPSY experiments also revealed an increase in the flux of *para*-H_2_ transfer into the Me group of propene over the course of the reaction, with the CHMe/Me signal ratio reaching a maximum of 1:0.8.

**Scheme 6:**
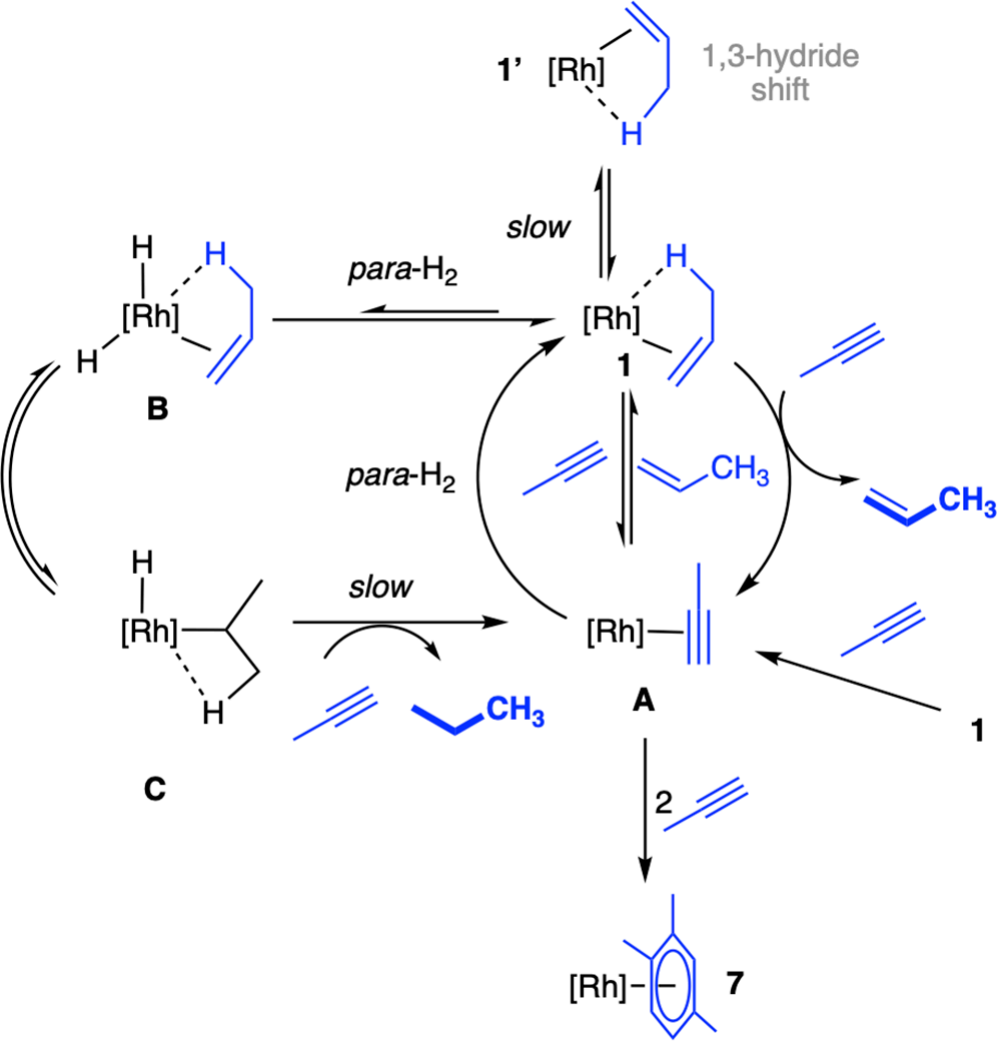
Suggested Catalytic Cycle for Propyne to Propane Hydrogenation Using *para*-H_2_ [Rh] = [Rh(^*t*^Bu_2_PCH_2_CH_2_P^*t*^Bu_2_)][BAr^F^_4_].

These observations report directly on a number of mechanistic details for propyne hydrogenation by **1** in the solid-state, but without the requirement for (time) expensive isotopic labeling in the substrates (i.e., D for H), or perturbation of reaction kinetics that comes from the installation of such labels.^65^Scheme 6 articulates these details in a proposed catalytic cycle. First, the polarization enrichment of the *cis* C–H sites in propene supports a dominant syn-addition of H_2_ via propyne and propene intermediates complexes **A** and **1**, respectively. Second, that all sites in the resulting free propene show polarization enhancement from *para*-H_2_ incorporation necessitates a mechanism in which both a slower isomerization, via a 1,3-hydride shift (complex **1′**),^15,66^ and rate-determining reductive elimination from a propyl hydride intermediate (**C**) operates for propyne hydrogenation. Finally, the enhancement of the methyl signal in the first measurement indicates that processes akin to level anti-crossing effects also operate under these ALTADENA condition,^67^ as this behavior cannot be accounted for by an isomerization process.^68^ While using hyperpolarization to report on mechanism in solution-based catalysis has been established for over 30 years,^69^ our observations here open up further the possibility for the informative interrogation of mechanism in molecular solid-state catalysis through a simple approach that does not perturb the thermodynamic or kinetic profile by labeling. PHIP has also been used to report on mechanism in classical heterogeneous hydrogenation catalysis,^30^ for example, in the semi-hydrogenation of propyne using CeO_2_ that shows a dependence of the arrangement of the surface atoms.^70^

^1^H NMR signals for propane also appeared in OPSY measurements, but their intensity initially fell to zero over ∼130 s with signals for propene only then remaining for the next ∼300 s. After the alkyne concentration had fallen to the point that propene hydrogenation is now competitive, propane was again detected as the major signal until all the propene was consumed, but now very slowly (∼30 min total) compared with 2 min for **1** mediating the hydrogenation of propene, for example, Figure 1A. We interpret this latter behavior as being due to the formation of cyclotrimerization product **7**, which is indeed observed as the only organometallic species after one charge. Consistent with this change in speciation is the significantly reduced catalytic activity seen when a second reaction cycle is performed. Taking independently prepared crystalline **7** and examining its activity in propyne hydrogenation using *para*-H_2_ also resulted in a comparative ∼130-fold drop in propene signal intensity (flux), consistent with the observation that **7** is a poor, at best, solid–gas hydrogenation catalyst. The change in catalytic flux of gas-phase PHIP again reports on changes in catalyst speciation at the molecular level.

#### 2.4.5 Butyne: Complex Gas-Phase Mixtures, Induction Periods, and Direct Observation of Catalyst Speciation

**Scheme 7:**
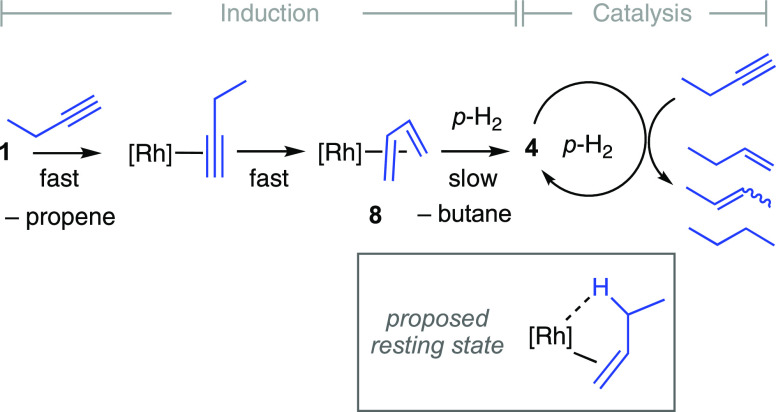
Proposed Reactivity of Complex **1** with 1-Butyne/*para*-H_2_ [Rh] = [Rh(^*t*^Bu_2_PCH_2_CH_2_P^*t*^Bu_2_)][BAr^F^_4_].

The reaction with 1-butyne and catalyst **1** at 25 °C showed a similar behavior to the hydrogenation of propyne, but now a more complex product mixture is formed through sequential alkyne then alkene hydrogenation that is coupled with double bond isomerization^39^ (Scheme 7). Heterogeneous catalysts have previously been reported for 1-butyne solid–gas PHIP.^29^ For example, the supported Cu/Si_2-700_ catalyst mediates the semi-hydrogenation of 1-butyne at temperatures in excess of 300 °C.^71^

**Figure 4:**
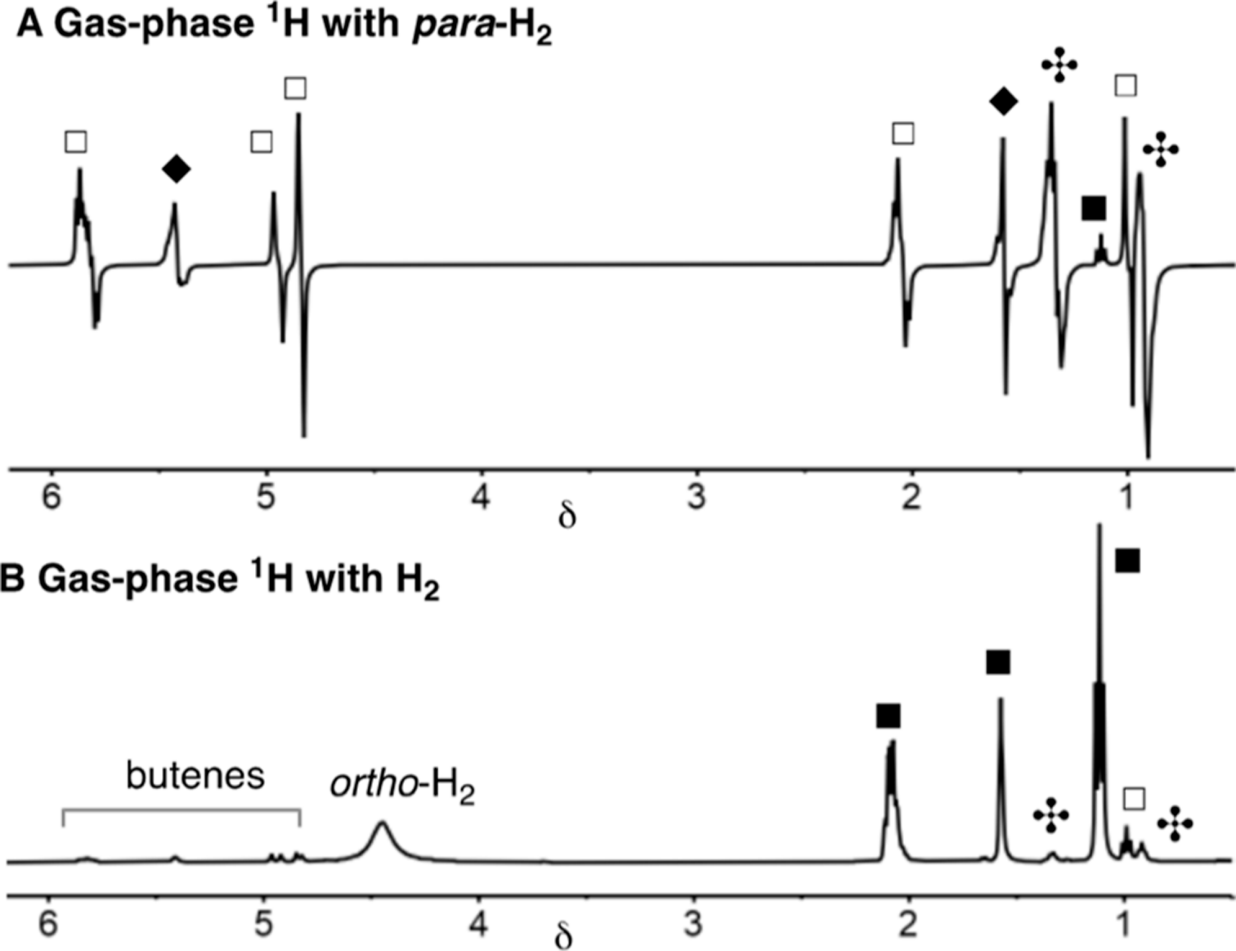
(A) Single-scan gas-phase ^1^H NMR spectrum from the hydrogenation of 1-butyne using **1** and *para*-H_2_ at 298 K: □ 1-butene; ◆ 2-butene; 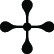 butane; and ■ 1-butyne (B) as above using thermally polarized cylinder H_2_.

The resulting PHIP enhanced signals, under PASADENA conditions at the early stages of catalysis, allow for all the signals of the hydrogenated products to be clearly observed and differentiated after a single scan, that is, 1-butene, 2-butenes (from isomerization^15^), and butane. Figure 4 contrasts the ^1^H NMR spectra where gas-phase PHIP is observed using *para*-H_2_ with catalysis using normal cylinder H_2_ at the same time point and demonstrates the levels of enhancement observed in the hydrogenated products using pre-catalyst **1** and *para*-H_2_. The analogous OPSY data confirmed dominant *cis*-H_2_ transfer to form 1-butene, and butane, with a small amount of 2-butenes also observed. An induction period is observed before productive catalysis starts, and that is followed by a significant increase in signal intensity that lasts for ∼60 s. We interpret this to indicate the rapid formation of butadiene complex **8** from isomerization of 1-butyne, as observed in the stoichiometric studies (Scheme 3) which then undergoes a relatively slower reaction with *para*-H_2_ to form active catalyst **4** (Scheme 7). After one hydrogenation cycle, complex **4** is observed as the only organometallic product. No cyclotrimerization product, **9**, is formed consistent with its very slow formation under stoichiometric conditions from the butadiene complex **8**. Further support for the initial formation of complex **8**, and the resulting induction period, comes from that independently prepared **4** mediates the hydrogenation of 1-butyne without an induction period, and that no induction period is observed for the hydrogenation of propyne.

Surprisingly, during the early stages of catalysis, very broad PHIP signals appear at ∼δ −0.78 in the region associated with agostic Rh···H–C interactions, with an apparent antiphase peak separation of ∼120 Hz alongside, peaks at δ 3.93 and 3.52 in the region associated with bound alkene ligands.^72^ As the intensity of these signals increases dramatically if the sample is raised toward the detection coils, we conclude that they arise from species located within the solid-catalyst matrix. Given the chemical shifts observed, we tentatively suggest this to be a butene coordinated complex, such as complex **6**,^39^ but note the potential for the existence of dihydrogen complexes that would also present broad high-field signals.^73^ Inspection of the sample, post-catalysis, reveals that a glassy-solid had formed. We suggest that mobility is thus created within the solid, which enables the detection of the resting state in solid/gas catalysis using PHIP without recourse to solid-state NMR methods.

### 2.5 PHIP Using a Normal Cylinder of H_2_ at Room Temperature

**Figure 5:**
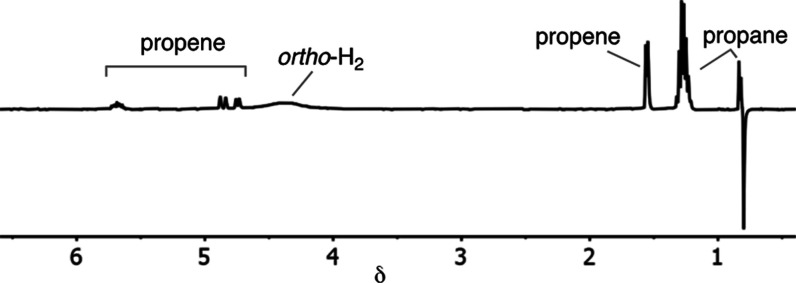
Single-scan gas-phase ^1^H NMR spectrum from the hydrogenation of propene using **1** and normal H_2_ at 25 °C after 10 s of catalysis.

As a further demonstration of the high levels of polarization transfer efficiency in propene hydrogenation using SMOM catalyst **1**, remarkably PHIP is observed in the product propane using H_2_ taken directly from a standard high-purity cylinder stored at room temperature (Figure 5). This represents an ∼19 fold enhancement.^74^ These data were collected using a standard 45° excitation-pulse as required for the detection of PHIP,^60^ under our standard conditions after 10 s of catalysis. Similar spectra are obtained using 1-butene at 25 °C (Figure S75). While in principle, all pairwise H_2_ additions should show PHIP under these conditions, such examples are rare^22,23,75,76^ due to a combination of slower turnover and/or lower polarization efficiency than found for **1**, which results in relaxation of hyperpolarized nuclear spin isomers before measurement. The ability to use normal H_2_ at 25 °C demonstrates that SMOM catalyst **1** removes the requirement for specialist equipment for *para*-H_2_ generation for the observation of PHIP in alkene hydrogenation as the retention of hyperpolarization is so efficient at this early stage of reaction.

### 2.6 Changing the SMOM Catalyst to [Rh(Cy_2_PCH_2_CH_2_PCy_2_)(C_3_H_6_)][BAr^F^_4_], **10**: Anion Coordination Quenches Catalytic Activity

**Scheme 8:**
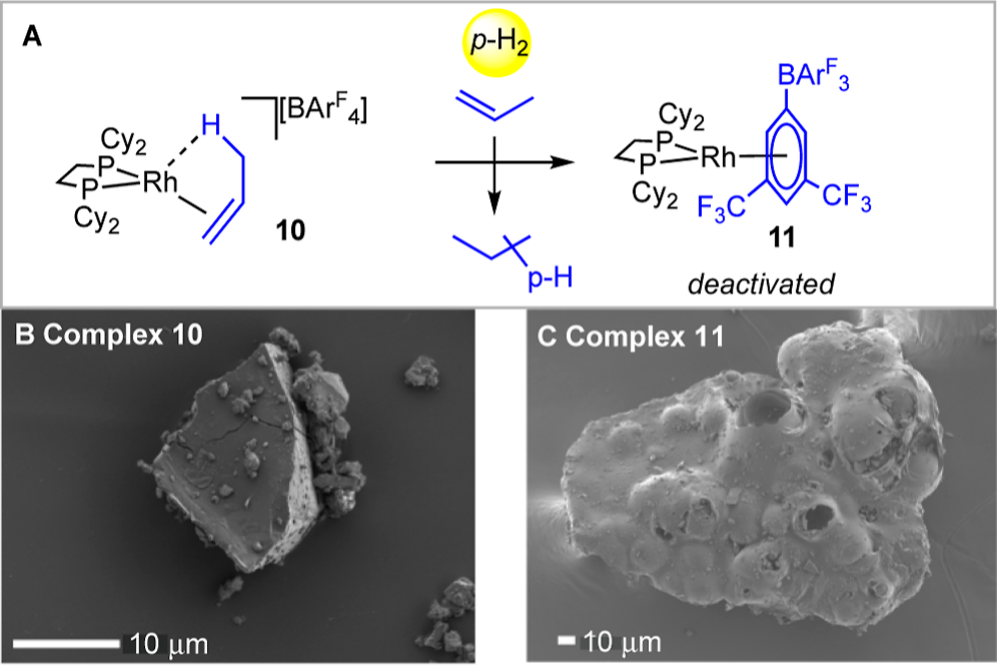
(A) Reaction of Complex **10** with *para*-H_2_ and the Formation of Deactivation Product **11**; (B) SEM Image of **10**; (C) SEM Image of **11** Post-Catalysis

The preceding observations were not unique to the ^*t*^Bu system, **1**. To demonstrate broader utility, we have also briefly investigated the effect of changing the substituents on the chelating phosphine ligand, by swapping ^*t*^Bu groups for less bulky Cy. Under our standard conditions (2 mg catalyst, 1:2 *para*-H_2_:propene) single-crystalline [Rh(Cy_2_PCH_2_CH_2_PCy_2_)(C_3_H_6_)][BAr^F^_4_], **10**,^15^ is initially shown to be an excellent catalyst for solid–gas polarization transfer to propene to form hyperpolarized propane, with the first measurement in high field conditions showing ∼85% transfer levels. However, subsequent measurements show a large attenuation in both PHIP and catalytic turnover, while a recharge showed significantly reduced follow-on activity. Analysis of the solid post-catalysis by both ^31^P{^1^H} SSNMR and solution NMR (CD_2_Cl_2_, −70 °C) showed exclusive formation of the [BAr^F^_4_]^−^ coordinated zwitterion with a η^6^-bound arene group, [Rh(Cy_2_PCH_2_CH_2_PCy_2_){η^6^-(CF_3_)_2_(C_6_H_4_)}BAr^F^_3_], **11**,^40^ which must be a very poor, but still active, hydrogenation catalyst, Scheme 8. This is the ultimate decomposition product of hydrogenation of propene complex [Rh(Cy_2_PCH_2_CH_2_PCy_2_)(C_3_H_6_)][BAr^F^_4_] in the solid-state.^44^ Thus, under conditions of excess H_2_, rapid hydrogenation of propene occurs, but competitive anion coordination quickly quenches activity. This is similar to the formation of the cyclotrimerization product **7** in propyne hydrogenation using **1**. The ^31^P{^1^H} SSNMR of **11** shows very broad signals at δ 91.4 and ∼δ 71^77^ (fwhm ∼2100 Hz, compared with **10** fwhm = 500 Hz) that indicate the formation of an amorphous phase, and consistent with this, SEM analysis of material pre- and post-catalysis shows that significant degradation of the crystal has occurred. Collectively, these data confirm how careful choice of the phosphine substituents is necessary if long catalyst lifetimes are to be maintained,^39^ as evident by the fact the ^*t*^Bu groups in **1** promote the formation of Rh(III) hydride **4** in solid-state. Thus, catalytic flux from gas-phase PHIP again directly reports on speciation changes in the solid-state.

## 3 Conclusions

PHIP has been widely used to study mechanism in molecular homogeneous catalysis in solution,^21,69,78^ and there is now a significant body of work dedicated to gas-phase PHIP using classical heterogeneous catalysts.^29,30^ Our crystalline SMOM systems clearly establish a connection between molecular catalysis (rate, selectivity through precise ligand design, and atomic-level resolution of molecular structure) with heterogeneous systems (product/catalyst separation, stability, and recyclability). The highly active “single-site” nature of our SMOM system, and attendant high catalytic flux with retention of polarization in the products, also allows for the real-time interrogation of an evolving molecular heterogeneous catalyst system. These results point to future opportunities for the straightforward creation of high polarization levels in simple hydrocarbons at ambient temperatures using solid-state organometallics. Such applications may well useful be in scenarios where hyperpolarized products are required for imaging applications in catalysis or medical diagnostics,^79,80^ to directly report of mechanism without recourse to isotopic labeling, or to indirectly report on catalyst structure in real-time.

## Supporting Information Available

Key NMR spectra, experimental and characterization data for the compounds reported including single-crystal X-ray diffraction studies, and experimental and fitting details of the XAS experiments (PDF)

## Abbreviations

*para*-H_2_: *para*-hydrogen
ALTADENA: adiabatic longitudinal transport after dissociation engenders net alignment
PASADENA: *para*-hydrogen and synthesis allow dramatically enhanced nuclear alignment
