# Supplementary material for: Mechanistic Insights into Molecular Crystalline Organometallic Heterogeneous Catalysis through Parahydrogen-Based Nuclear Magnetic Resonance Studies
Source: J Am Chem Soc. 2023 Jan 23;145(4):2619–29. doi: 10.1021/jacs.2c12642 (PMC9896567; doi:10.1021/jacs.2c12642)
Supplement: Supplementary file 1 — ja2c12642_si_001.pdf [file ja2c12642_si_001.pdf]

Supporting Information for
Mechanistic Insight into Molecular Crystalline Organometallic Heterogeneous Catalysis Through Parahydrogen Based Nuclear Magnetic Resonance Studies

Matthew R. Gyton,[a,b] Cameron G. Royle,[a,c] Simon K. Beaumont,[d] Simon B. Duckett[a,b*], Andrew S. Weller[a*]

[a] Department of Chemistry, University of York, York, YO10 5DD (U. K.)

[b] Centre for Hyperpolarisation in Magnetic Resonance, Department of Chemistry, University of York, Heslington, York, YO10 5DD (U. K.)

[c] Department of Chemistry, University of Oxford, Mansfield Road, Oxford, OX1 3TA (U.K.)

[d] Department of Chemistry, Durham University, South Road, Durham DH1 3LE (U. K.)

Table of Contents

General Procedures 3

Synthesis 5

Preparation of [Rh(^{t}Bu$_2$PCH$_2$CH$_2$P^{t}Bu$_2$)(nbd)][BAr$^F_4$] 5

Spectroscopic Data for [Rh(^{t}Bu$_2$PCH$_2$CH$_2$P^{t}Bu$_2$)(nbd)][BAr$^F_4$] 5

Preparation of [Rh(^{t}Bu$_2$PCH$_2$CH$_2$P^{t}Bu$_2$)(propene)][BAr$^F_4$] 6

Spectroscopic Data for [Rh(^{t}Bu$_2$PCH$_2$CH$_2$P^{t}Bu$_2$)(propene)][BAr$^F_4$] 6

Preparation of [Rh(^{t}Bu$_2$PCH$_2$CH$_2$P^{t}Bu$_2$)($C_6H_3Me_3$)][BAr$^F_4$] 11

Spectroscopic Data for [Rh(^{t}Bu$_2$PCH$_2$CH$_2$P^{t}Bu$_2$)($C_6H_3Me_3$)][BAr$^F_4$] 12

Speciation and Post-Catalysis Experiments 14

Catalytic Scale Speciation 14

Bulk Scale Analysis 24

Scanning Electron Microscopy 31

X-Ray Absorption Spectroscopy 36

Gas-phase NMR of 1-Butyne to 1,3-Butadiene Isomerisation 42

Solid/Gas Alkyne Cyclotrimerisation 45

Solution Alkyne Cyclotrimerisation 51

Gas Phase Catalysis 52

General Procedure 52

Representative Spectra: Propene 52

Representative Spectra: 1-Butene 59

Representative Spectra: Propyne 63

Representative Spectra: 1-Butyne 68

Representative Spectra: 1-Butene/Propene Mixtures 73

Single Crystal X-Ray Diffraction 76

References 78

General Procedures

All manipulations, unless otherwise stated, were performed under an inert (argon or nitrogen, BOC, N4.8 purity) or dihydrogen (BOC, N4.0) atmosphere using standard Schlenk line and glovebox (<0.1 ppm H_2O/O_2) techniques. Glassware was oven-dried at 140 °C overnight and flame dried under vacuum prior to use. All solvents were degassed by three successive freeze-pump-thaw cycles and stored over activated 3 Å molecular sieves under inert gas in resealable glass ampoules fitted with PTFE high vacuum stopcocks (Kontes Hi-Vac, J. Young or Rotaflo HP). CH_2Cl_2, pentane and hexane were dried using a commercially available solvent system (Innovative Technologies or MBraun) by passage through stainless steel columns containing activated alumina.[1] Heptane was purchased anhydrous from Sigma-Aldrich and decanted by cannula into resealable glass ampoules and stored as above. CD_2Cl_2 and 1,2-$C_6H_4F_2$ (pre-dried by stirring over activated alumina) were dried over CaH_2, before vacuum transfer and storage as above. All solution phase NMR were prepared on a greaseless high vacuum line (<1 x 10^{-5} mbar) by condensation of the solvent under static vacuum onto solid samples in 5 mm thin wall NMR tubes fitted with high vacuum PTFE (J. Young) valves. $[Rh(nbd)_2][BAr^F_4]$,[2] $^tBu_2PCH_2CH_2P^tBu_2$[3] $[\{RhH(\mu\text{-}H)(^tBu_2PCH_2CH_2P^tBu_2)\}_2][BAr^F_4]$, **4**[3] and $[Rh(Cy_2PCH_2CH_2PCy_2)(C_3H_6)][BAr^F_4]$, **10**[4] were prepared by literature methods.

Solution phase NMR spectra were recorded on a Bruker Avance III HD 500 MHz spectrometer using a TBO probe (^1H = 500.22 MHz, ^{13}C = 125.80 MHz, ^{31}P = 202.50 MHz) or Bruker Avance III HD 600 MHz spectrometer using a BBO probe (^1H = 600.09 MHz, ^{13}C = 150.91 MHz, ^{31}P = 242.95 MHz) or a Bruker Avance III 400 MHz spectrometer using a BBI probe (^1H = 400.12 MHz, ^{13}C = 100.61 MHz) at 298 K unless otherwise specified. Residual proteo solvent was used as reference for ^1H and $^{13}C\{^1H\}$ spectra in deuterated solvent samples.[5] $^{31}P\{^1H\}$ NMR spectra were externally referenced to 85% H_3PO_4. ^1H assignments were aided by $^1H\{^{31}P\}$ experiments. All chemical shifts (δ) are quoted in ppm and coupling constants (*J*) in Hz. NMR assignments were aided by 2D experiments (^1H-^1H-COSY, ^1H-^{13}C-HSQC, ^1H-^{13}C-HMBC) where required.

Gas phase NMR spectra were recorded on a Bruker Avance III 400 MHz spectrometer using a BBI probe (^1H = 400.12 MHz, ^{13}C = 100.61 MHz), operating Topspin 3.6.2 at 298 K in 5 mm thin wall NMR tubes fitted with high vacuum PTFE (J. Young) valves and are referenced arbitrarily to the following values: propane (δ = 0.90, 1.37), propene (δ = 1.65, 4.85, 4.96, 5.78), butane (δ = 0.90, 1.33), 1-butene (δ = 0.99, 2.05, 4.89, 4.95, 5.83), 2-butene (δ = 1.57, 5.41), propyne (δ = 1.40, 1.63), 1-butyne (δ = 1.11, 1.58, 2.09) and 1,3-butadiene. (4.95, 5.08, 6.31).^1H NMR spectra are recorded with a single scan, 45° pulse, 1 s acquisition time (aq) and 1 s recycle delay (d1) with 48074 datapoints (td) over a 60.08 ppm spectroscopic window (sw). $^{13}C\{^1H\}$ INEPT spectra are recorded in the gas phase with a single scan with a 0.6 s acquisition time (aq) and 0.01 s recycle delay (d1) with 28842 datapoints (td) over a 238.91 ppm spectroscopic window (sw). ^1H-^1H COSY were recorded in the gas phase with two scans per increment (ns) over a spectroscopic window of 15.02 ppm (sw F1, sw F2) with 128 datapoints in the indirect dimension (td F1) and 2402 in the direct (td F2) with 0.2 s (aq F2) and 0.01 s (aq F1) acquisition times and 0.01 s recycle delay (d1). ^1H-^{13}C-HMQC spectra were recorded in the gas phase with two scans per increment (ns) over a spectroscopic window of 120 ppm (sw F1) and 10 ppm (sw F2) with 128 datapoints in the indirect dimension (td F1) and 1198 in the direct (td F2) with 0.15 s (aq F2) and 0.005 s (aq F1) acquisition times and 0.01 s recycle delay (d1). OPSY spectra in the gas phase were recorded using the OPSYdq pulse sequence with a 1 μsec recycle delay (d1),

16 µsec gradient recovery delay (d16), 30% gradient strength (gpz1) and a 500 µsec gradient pulse length (p16).[6]

Solid state NMR spectra were recorded on a Bruker wide bore Avance III HD spectrometer (^{13}C = 100.66 MHz; ^{31}P = 162.03 MHz) in 4.0 mm zirconia rotors with a MAS rate of 10 kHz unless otherwise specified. Post-catalysis samples were prepared in 10 mm OD NMR tubes fitted with coaxial high vacuum PTFE (J. Young) valves. Rotors were packed and sealed with Kel-F caps or zirconia caps for variable temperature experiments in an argon filled glovebox. Spectra are referenced externally to $Si(CH_3)_4$ or H_3PO_4 using the secondary references adamantane (^{13}C δ = 29.5 for the shielded methylene resonance)[7] or triphenylphosphine (^{31}P, δ = -9.3).[8]

Elemental microanalyses were performed by Dr Graeme McAllister on an Exeter Analytical Inc. CE-440 at the University of York. Electrospray Ionisation Mass Spectrometry (ESI-MS) was carried out using a Bruker compact Time of Flight mass spectrometer by Mr Karl Heaton at the University of York. Gas Chromatography was performed by Dr Scott Hicks on a Thermo Scientific Trace 1300 at the University of York.

Synthesis

Preparation of [Rh(^{t}Bu$_2$PCH$_2$CH$_2$P^{t}Bu$_2$)(nbd)][BAr$^F_4$]

The title compound has been prepared previously through a more circuitous procedure;[9] a more convenient preparation is outlined below:

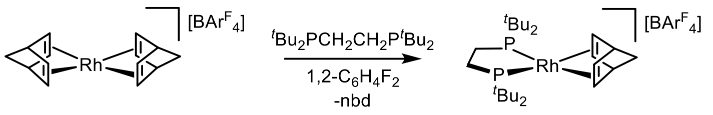

A solution of ^{t}Bu$_2$PCH$_2$CH$_2$P^{t}Bu$_2$ (317mg, 994 µmol) in 1,2-$C_6H_4F_2$ (*ca.* 10 mL) was added dropwise via cannula over 5 minutes to a solution of [Rh(nbd)$_2$][BAr$^F_4$] (1.14 g, 994 µmol) also in 1,2-$C_6H_4F_2$ (*ca.* 30 mL). The red solution was stirred for 1 hour at ambient temperature before the crude product was precipitated by the addition of excess hexane (*ca.* 300 mL); the suspension was filtered and washed with further hexane (2 x 5 mL). Slow diffusion of excess heptane (*ca.* 300 mL) at room temperature into a solution of the crude compound in 1,2-$C_6H_4F_2$ (*ca.* 50 mL), decantation of the supernatant and drying of the crystals under vacuum overnight afforded the title compound as deep red blocks (1.30 g, 944 µmol, 95%).
Data matches previously published values.

Spectroscopic Data for [Rh(^{t}Bu$_2$PCH$_2$CH$_2$P^{t}Bu$_2$)(nbd)][BAr$^F_4$]

^{1}H NMR (400.12 MHz, CD_2Cl_2, 298 K) δ 7.72 (br. m, 8H; Ar$^F_4$), 7.56 (s, 4H, Ar$^F_4$), 5.57-5.55 (m, 4H, nbd alkene C*H*), 4.13-4.10 (m, 2H, nbd CH), 1.90-1.78 (m, 4H, PC*H*$_2$), 1.78 (s br., 2H, nbd C*H*$_2$), 1.31 (d, $^3J_{HP}$ = 13.1, PC*H*$_3$).
^{31}P{^{1}H} NMR (161.98 MHz, CD_2Cl_2, 298 K) δ 84.0 (d, $^1J_{PRh}$ = 150).

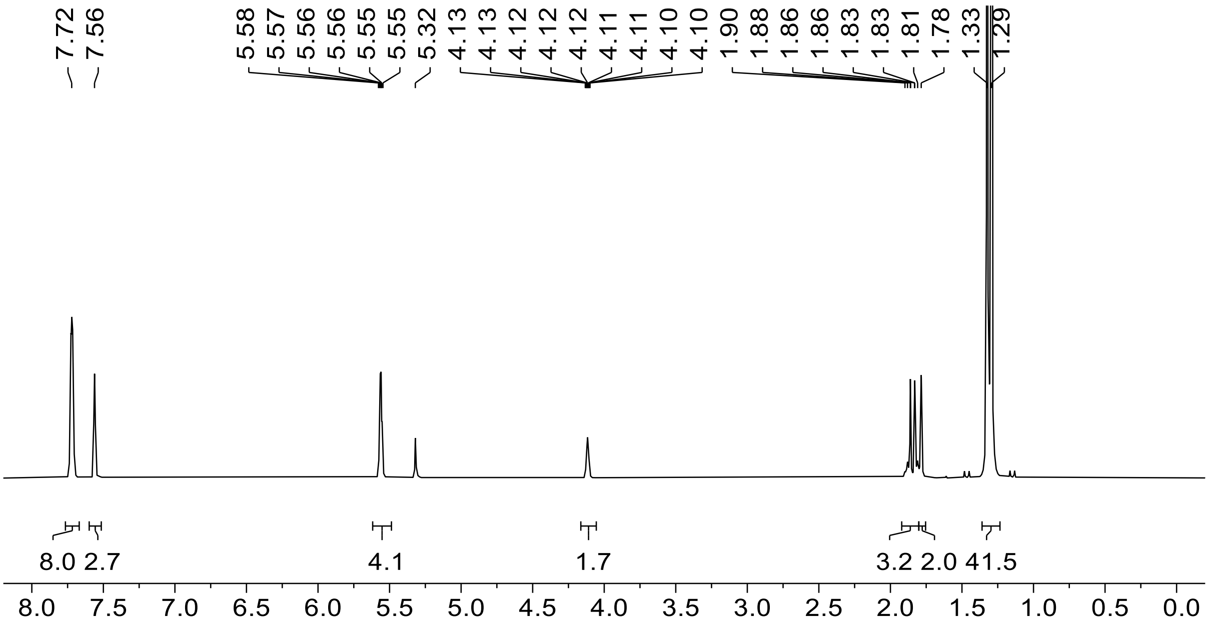

Figure S1. *^{1}H NMR spectrum of [Rh(^{t}Bu$_2$PCH$_2$CH$_2$P^{t}Bu$_2$)(nbd)][BAr$^F_4$] (400.12 MHz, CD_2Cl_2, 298 K).*

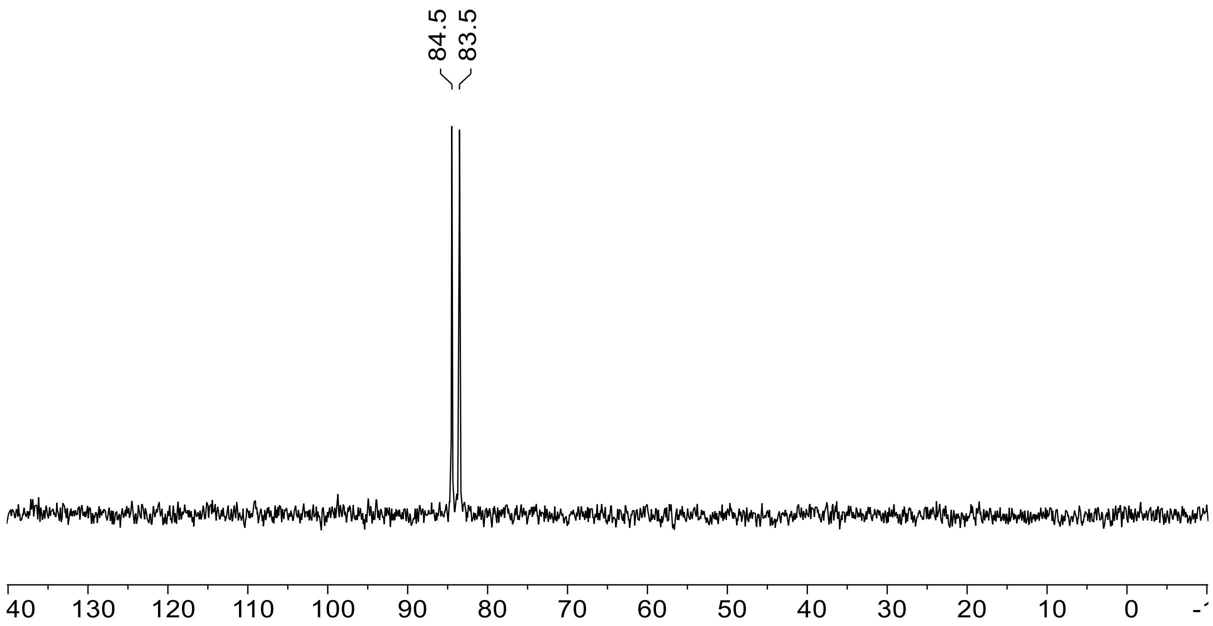

Figure S2. *$^{31}P\{^1H\}$ NMR spectrum of [Rh(^{t}Bu$_2$PCH$_2$CH$_2$P^{t}Bu$_2$)(nbd)][BAr^F$_4$] (161.98 MHz, CD$_2$Cl$_2$, 298 K).*

Preparation of [Rh(^{t}Bu$_2$PCH$_2$CH$_2$P^{t}Bu$_2$)(propene)][BAr^F$_4$]

Solution phase method:

A solution of [Rh(^{t}Bu$_2$PCH$_2$CH$_2$P^{t}Bu$_2$)(nbd)][BAr^F$_4$] (25 mg, 18 µmol) in 1,2-difluorobenzene (2 mL) was freeze-pump-thaw degassed and placed under an atmosphere of hydrogen (1 bar gauge) and stirred for 30 minutes at room temperature. Volatiles were removed *in vacuo* and the resulting orange solid re-dissolved in CH_2Cl_2 (2 mL), freeze-pump-thaw degassed and placed under an atmosphere of propene (1 bar gauge) and stirred at room temperature for 30 minutes before concentration *in vacuo* to approximately half the original volume and then layered with pentane. Storage at room temperature afforded [Rh(^{t}Bu$_2$PCH$_2$CH$_2$P^{t}Bu$_2$)(propene)][BAr^F$_4$] **1** as red-orange plates (23 mg, 17 µmol, 95%).

Solid/gas method:

Crystalline [Rh(^{t}Bu$_2$PCH$_2$CH$_2$P^{t}Bu$_2$)(nbd)][BAr^F$_4$] (150.4 mg, 109.2 µmol, sieved to below 150 µm crystal size) was placed under an atmosphere of hydrogen (1 bar gauge) for 90 minutes. The flask was evacuated and refilled with argon thrice before re-evacuation and placed under an atmosphere of propene (1 bar gauge). Storage at room temperature for 2 hours followed by removal of norbornane under vacuum afforded the title compound as a dark orange microcrystalline powder (118.6 mg, 89.40 µmol, 82%)

Spectroscopic Data for [Rh(^{t}Bu$_2$PCH$_2$CH$_2$P^{t}Bu$_2$)(propene)][BAr^F$_4$]

^{1}H NMR (500.23 MHz, CD_2Cl_2, 193 K) δ 7.73 (s, 8H, Ar^F$_4$), 7.54 (s, 4H, Ar^F$_4$), 5.00-4.91 (m, 1H, propene H$_2$C*H*CH$_3$) 4.87 (d br, *J* = 7, propene *H*$_2$CHCH$_3$), 3.36 (d br, *J* = 14, propene *H*$_2$CHCH$_3$), 1.98-1.84 (m, 2H, PC*H*$_2$), 1.73-1.59 (m, 2H, PC*H*$_2$), 1.26 (d, $^3J_{HP}$ = 14, 9H, PC(C*H*$_3$)$_3$), 1.78 (d, $^3J_{HP}$ = 14, 18H, PC(C*H*$_3$)$_3$), 1.05 (d, $^3J_{HP}$ = 14, 9H, PC(C*H*$_3$)$_3$), -0.64 (s br., 3H, propene H$_2$CHC*H*$_3$).

$^{13}C\{^1H\}$ NMR (125.98 MHz, CD_2Cl_2, 185 K) δ 161.4 (q, $^1J_{CB}$ = 50 Hz, Ar^F), 134.1 (s br., Ar^F), 128.1 (qq, $^2J_{CF}$ = 32 Hz, $^4J_{CF}$ = 3), 124.0 (q, $^1J_{CF}$ = 272, CF_3), 117.1 (sept., $^4J_{CF}$ = 4 Hz, Ar^F), 96.2 (m, propene $H_2\mathit{C}HCH_3$), 74.8 (m, propene $H_2\mathit{C}HCH_3$), 37.9 (d, $^1J_{CP}$ = 22, P*C*$(CH_3)_3$), 37.4 (d, $^1J_{CP}$ = 21, P*C*$(CH_3)_3$), 36.7 (d, $^1J_{CP}$ = 20, P*C*$(CH_3)_3$), 36.1 (d, $^1J_{CP}$ = 20, P*C*$(CH_3)_3$), 29.9 (br., PC($\mathit{C}H_3)_3$), 29.6 (br., PC($\mathit{C}H_3)_3$), 29.3 (br., PC($\mathit{C}H_3)_3$), 29.0 (br., PC($\mathit{C}H_3)_3$), 25.5 (m, P$\mathit{C}H_2$), 20.2 (m, P$\mathit{C}H_2$), 10.4 (m, propene $H_2CH\mathit{C}H_3$).

$^{31}P\{^1H\}$ NMR (202.53 MHz, CD_2Cl_2, 193 K) δ 116.4 (dd, $^2J_{PRh}$ = 211, $^3J_{PP}$ = 16), 112.3 (d, $^2J_{PRh}$ = 162, $^3J_{PP}$ = 16).

$^{13}C\{^1H\}$ CP MAS NMR (10 kHz spin rate, 162.04 MHz, 175 K) δ 168-160 (Ar^F), 140-115 (Ar^F), 96.7 (propene CH), 95.3 (propene C-H), 78.0-71.5 (propene CH_2), 40-20 ($^tBu_2PCH_2CH_2P^tBu_2$).

$^{31}P\{^1H\}$ CP MAS NMR (10 kHz spin rate, 162.04 MHz, 298 K) δ 115.4 (s br., fwhm = ~750 Hz).

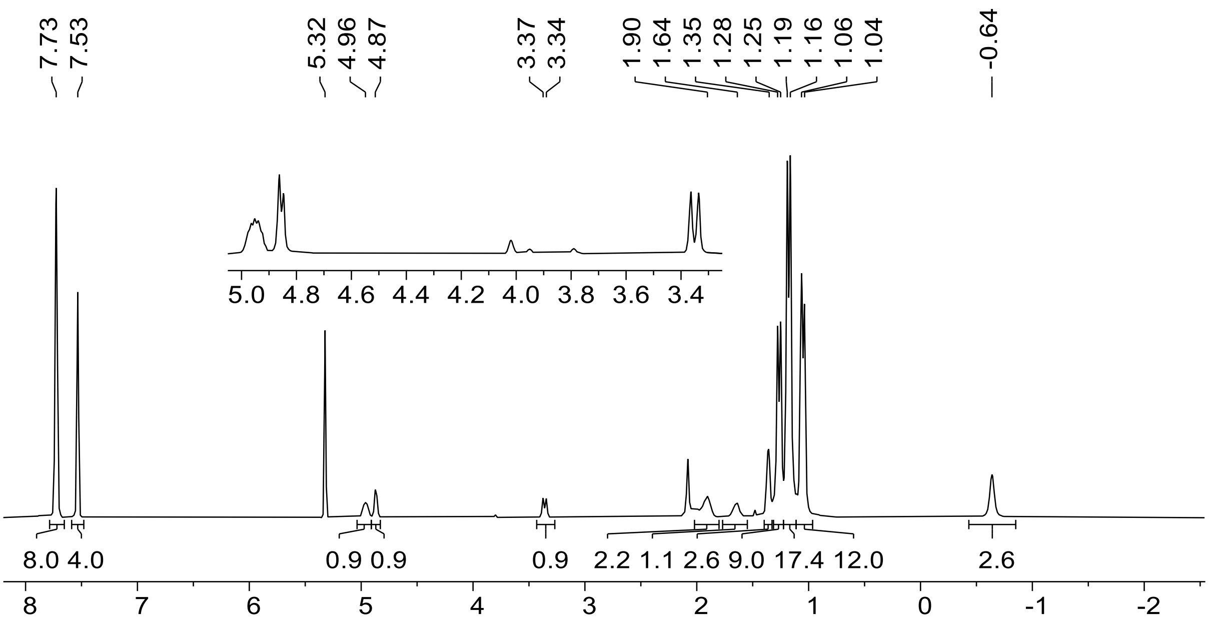

Figure S3. *^1H NMR Spectrum of [Rh($^tBu_2PCH_2CH_2P^tBu_2$)(propene)][BAr$^F_4$]* ***1****, (500.23 MHz, CD_2Cl_2, 193 K). Inset: $^1H\{^{31}P\}$ spectrum over the region 3.2-5.0 ppm.*

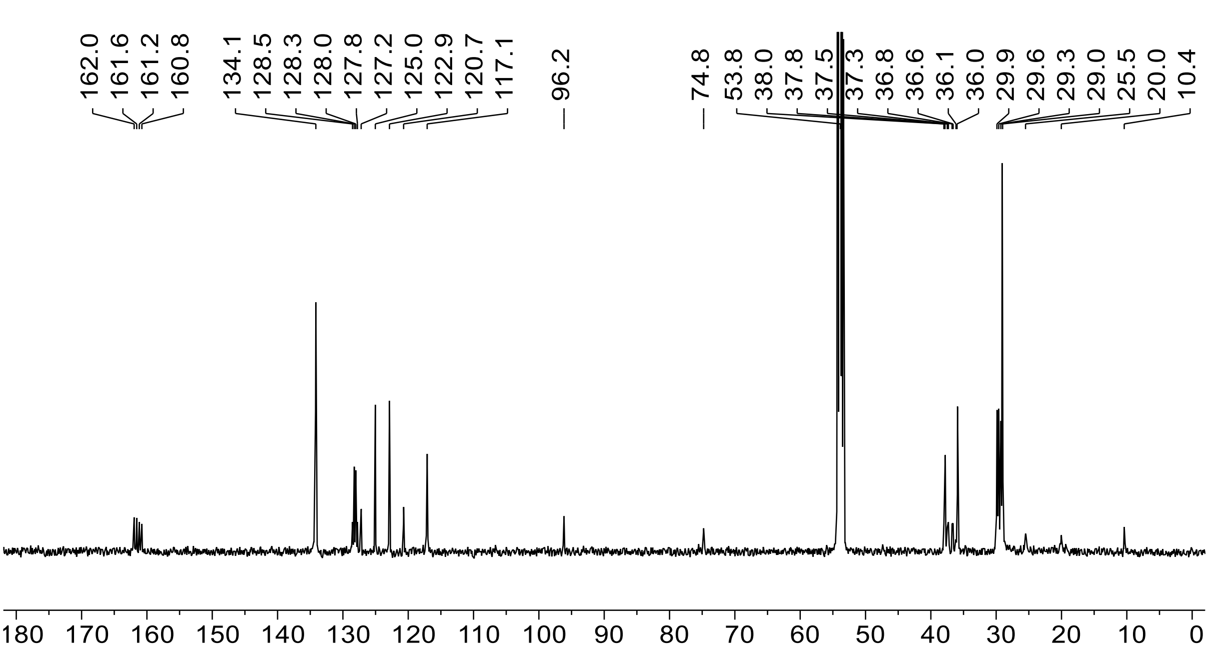

Figure S4. *$^{13}C\{^1H\}$ NMR Spectrum of [Rh($^tBu_2PCH_2CH_2P^tBu_2$)(propene)][BAr$^F{}_4$]* ***1****, (125.80 MHz, CD_2Cl_2, 185 K).*

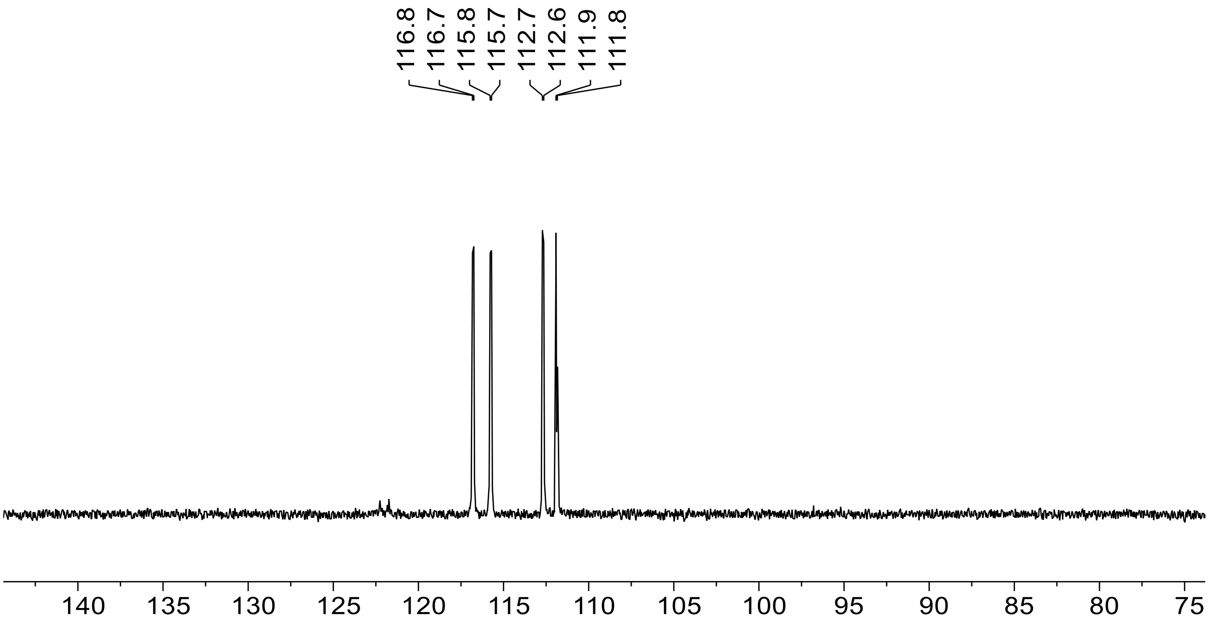

Figure S5. *$^{31}P\{^1H\}$ NMR Spectrum of [Rh($^tBu_2PCH_2CH_2P^tBu_2$)(propene)][BAr$^F{}_4$]* ***1****, (202.53 MHz, CD_2Cl_2, 193 K).*

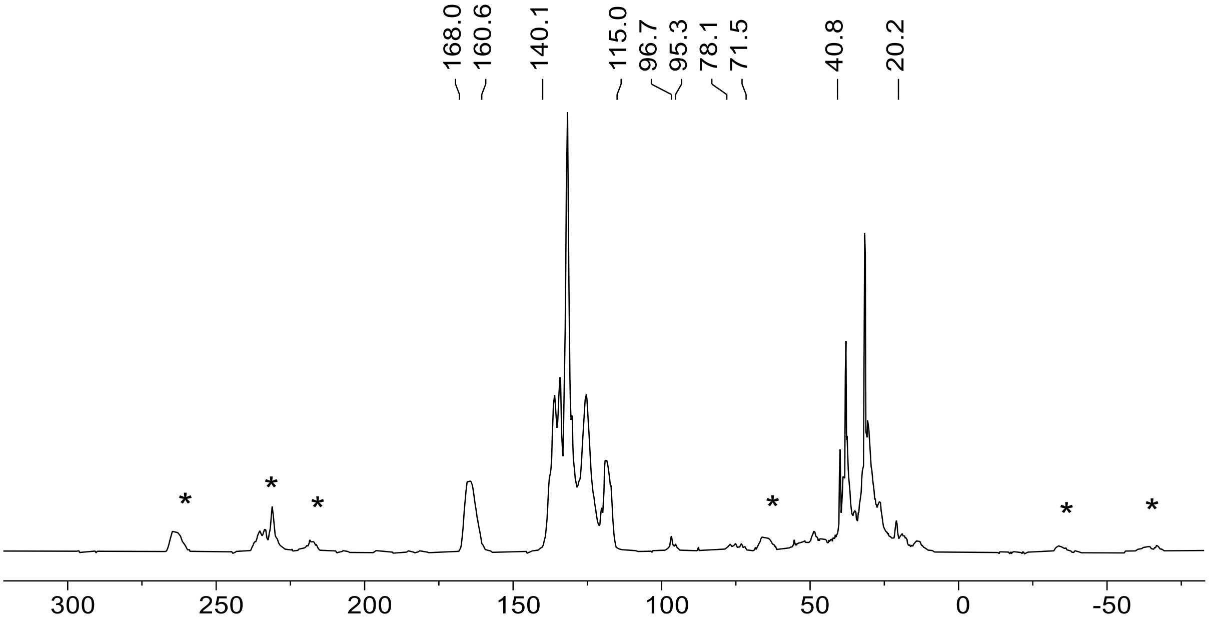

Figure S6. *$^{13}C\{^1H\}$ CP MAS NMR spectrum of [Rh($^tBu_2PCH_2CH_2P^tBu_2$)(propene)][BAr$^F{}_4$]* ***1****, (10 kHz spin rate, 100.66 MHz, 175 K). Spinning sidebands are indicated with *.*

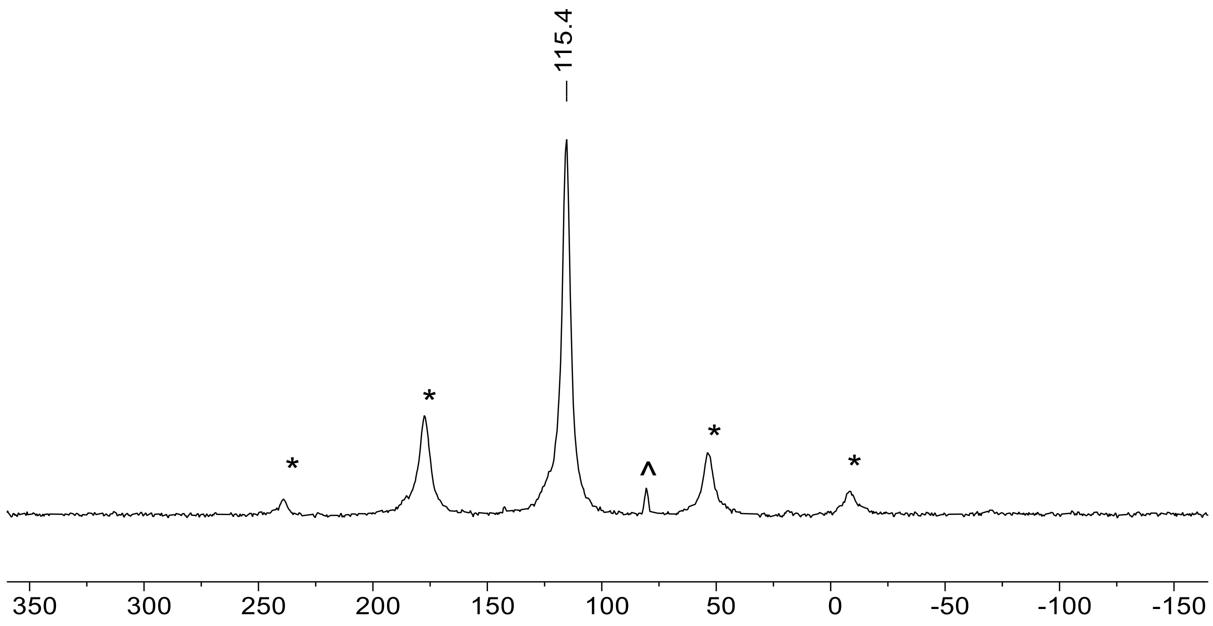

Figure S7. *$^{31}P\{^{1}H\}$ CP MAS NMR spectrum of [Rh($^{t}Bu_2PCH_2CH_2P^{t}Bu_2$)(propene)][$BAr^{F}_4$]* ***1****, (10 kHz spin rate, 162.04 MHz, 294 K). Residual [Rh($^{t}Bu_2PCH_2CH_2P^{t}Bu_2$)(nbd)][$BAr^{F}_4$] is indicated by ^ and spinning sidebands by *.*

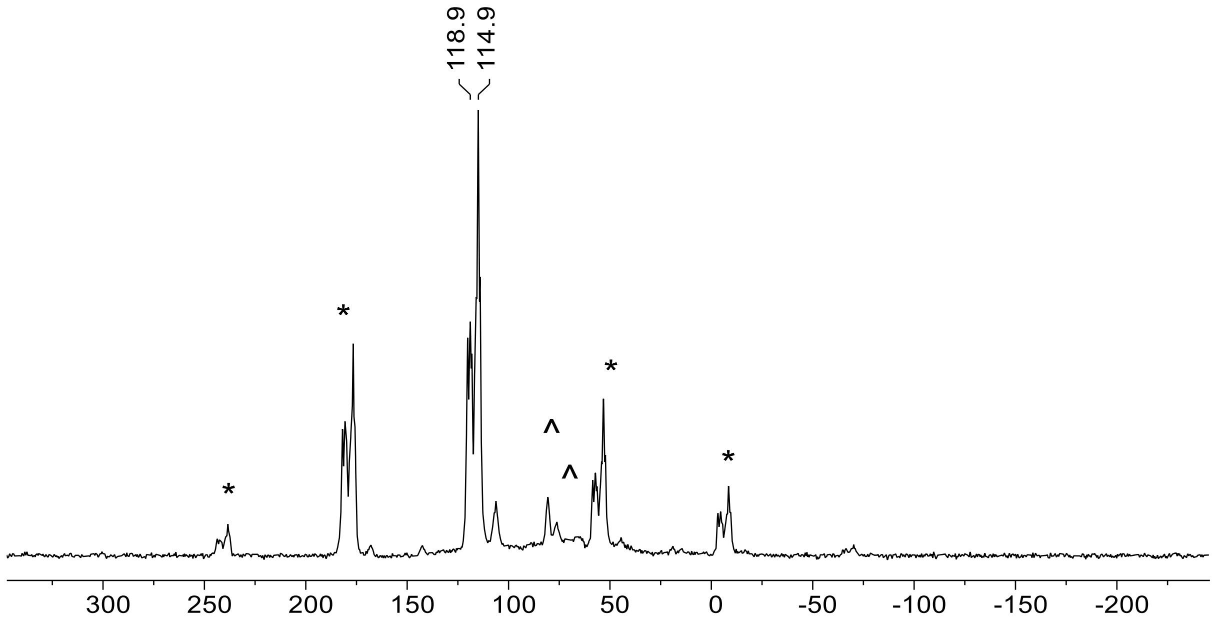

Figure S8. *$^{31}P\{^{1}H\}$ CP MAS NMR spectrum of [Rh($^{t}Bu_2PCH_2CH_2P^{t}Bu_2$)(propene)][$BAr^{F}_4$]* ***1****, (10 kHz spin rate, 162.04 MHz, 173 K). Residual [Rh($^{t}Bu_2PCH_2CH_2P^{t}Bu_2$)(nbd)][$BAr^{F}_4$] is indicated by ^ and spinning sidebands by *.*

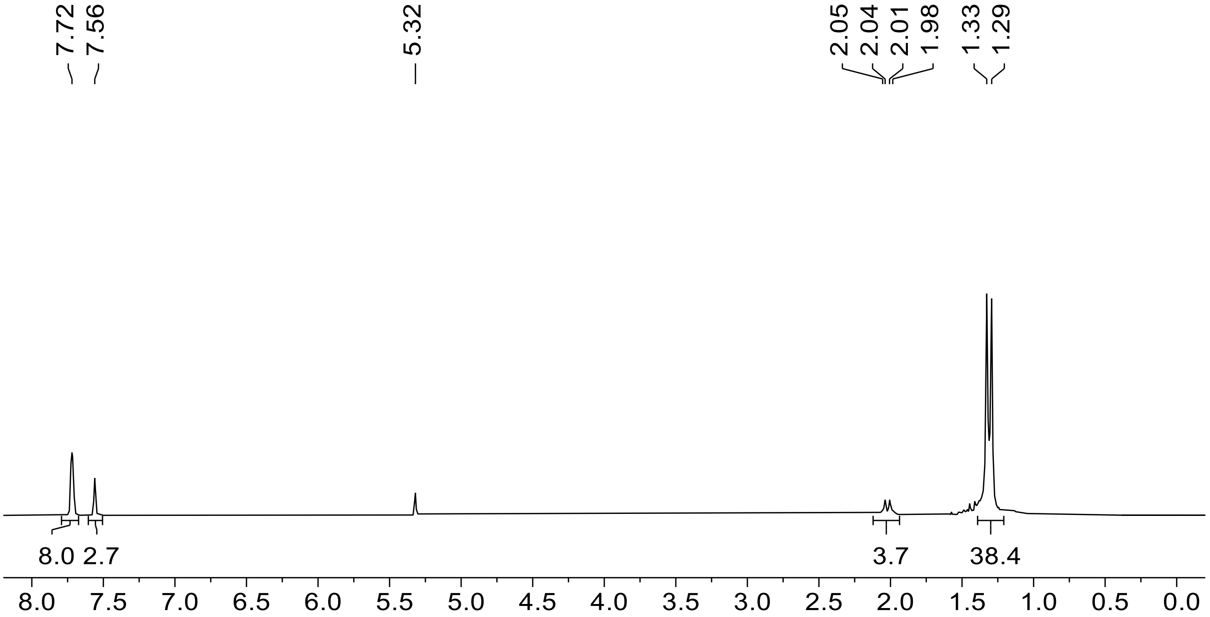

Figure S9. *^{1}H NMR Spectrum of [Rh(^{t}Bu$_2$PCH$_2$CH$_2$P^{t}Bu$_2$)(propene)][BAr$^{F}_4$]* ***1****, (400.11 MHz, CD_2Cl_2, 298 K).*

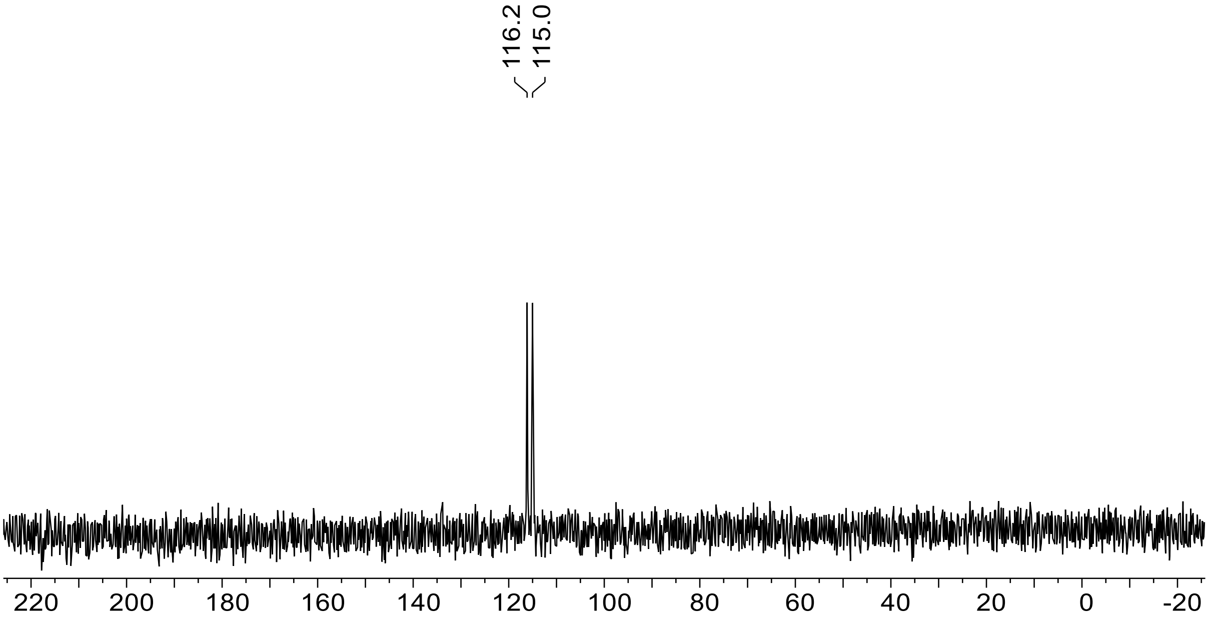

Figure S10. *^{31}P{^{1}H} NMR Spectrum of [Rh(^{t}Bu$_2$PCH$_2$CH$_2$P^{t}Bu$_2$)(propene)][BAr$^{F}_4$]* ***1****, (161.99 MHz, CD_2Cl_2, 298 K).*

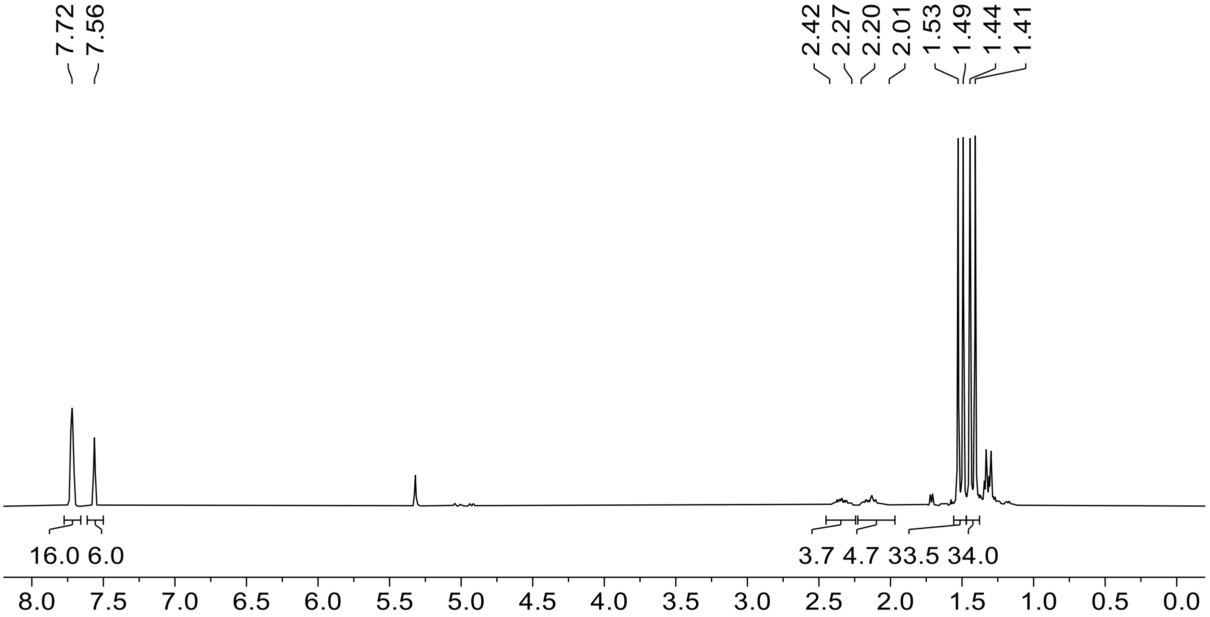

Figure S11. *^{1}H NMR Spectrum showing the formation of [Rh_2(^{t}$Bu_2PCH_2CH_2P^tBu_2$)$_2$(μ-CD_2)(μ-Cl)$_2$][BAr^F_4]$_2$* ***3*** *from storage of a solution of [Rh(^{t}$Bu_2PCH_2CH_2P^tBu_2$)(propene)][BAr^F_4]* ***1*** *in CD_2Cl_2 at room temperature for 24 hours (400.11 MHz, CD_2Cl_2, 298 K).*

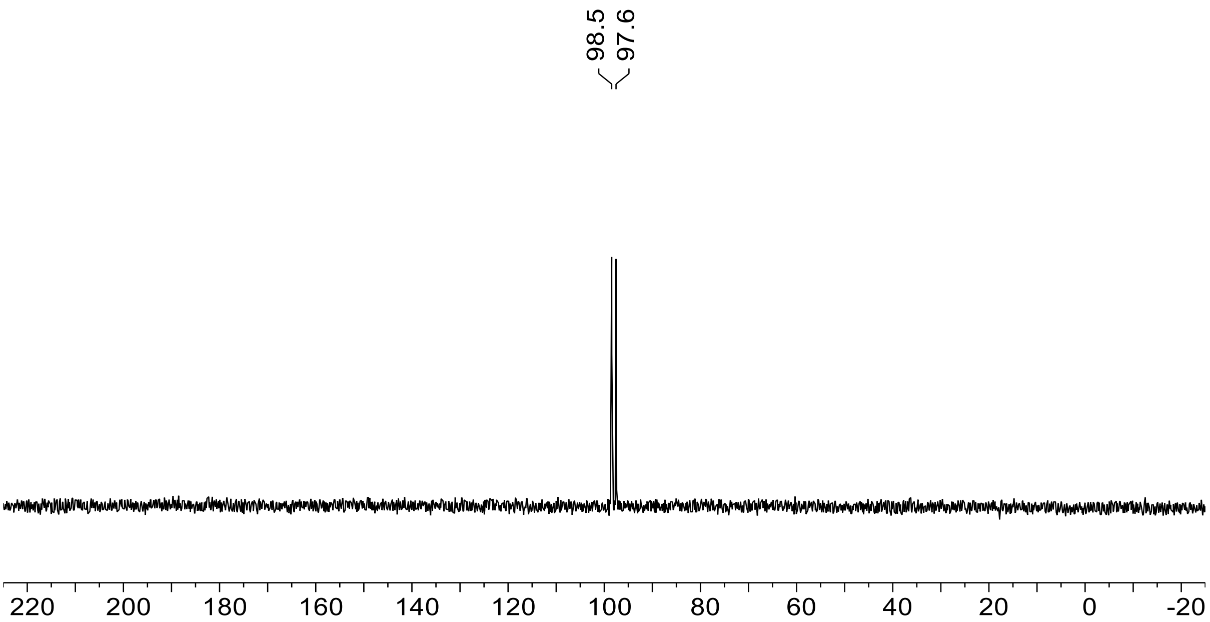

Figure S12. *^{31}P{^{1}H }NMR spectrum showing the formation of [Rh_2(^{t}$Bu_2PCH_2CH_2P^tBu_2$)$_2$(μ-CD_2)(μ-Cl)$_2$][BAr^F_4]$_2$* ***3*** *from storage of a solution of [Rh(^{t}$Bu_2PCH_2CH_2P^tBu_2$)(propene)][BAr^F_4]* ***1*** *in CD_2Cl_2 at room temperature for 24 hours (161.99 MHz, CD_2Cl_2, 298 K).*

Preparation of [Rh(^{t}$Bu_2PCH_2CH_2P^tBu_2$)($C_6H_3Me_3$)][BAr^F_4]

A solution of [Rh(^{t}$Bu_2PCH_2CH_2P^tBu_2$)(nbd)][BAr^F_4] (50 mg, 36 μmol) and 1,2,4-$CH_3C_6H_3$ (0.1 mL, 0.7 mmol, ~20 eq.) in CH_2Cl_2 (5 mL) was freeze-pump-thaw degassed and placed under an atmosphere of hydrogen (1 bar gauge) and stirred at room temperature for 5 minutes, over which time the initially dark orange solution lightened. The solution was freeze-pump-thaw degassed and placed under an atmosphere of

nitrogen and layered with excess hexane to yield the title compound as pale orange plates (44 mg, 31 μmol, 87%) after 16 hours.

Spectroscopic Data for [Rh(^{t}Bu$_2$PCH$_2$CH$_2$P^{t}Bu$_2$)(C$_6$H$_3$Me$_3$)][BAr$^F{}_4$]

^{1}H NMR (500.23 MHz, CD_2Cl_2, 298 K) δ 7.72 (s, 8H, Ar$^F{}_4$), 7.56 (s, 4H, Ar$^F{}_4$), 6.31 (d, $^3J_{HH}$ = 6.5 Hz, 1H, arene CH), 5.99 (d, $^3J_{HH}$ = 6.5 Hz, 1H, arene CH), 5.99 (s, 1H, arene CH), 2.41 (s, 3H, arene CH_3), 2.24 (s, 3H, arene CH_3), 2.19 (s, 3 H, arene CH_3), 1.98-1.80 (m, 2H, PC*H*$_2$), 1.54-1.42 (m, 2H, PC*H*$_2$), 1.22 (d, $^3J_{HP}$ = 13 Hz, 18H, PC(C*H*$_3$)$_3$), 1.21 (d, $^3J_{HP}$ = 13 Hz, 18H, PC(C*H*$_3$)$_3$).

^{13}C{^{1}H} NMR (150.91 MHz, CD_2Cl_2, 298 K) δ 162.2 (q, $^1J_{CB}$ = 50 Hz, Ar^F), 135.2 (s br., Ar^F), 129.3 (qq, $^2J_{CF}$ = 32 Hz, $^4J_{CF}$ = 3), 124.9 (q, $^1J_{CF}$ = 272, CF_3), 123.8 (m, arene C), 121.9 (m, arene C), 120.1 (m, arene C), 117.9 (sept., $^4J_{CF}$ = 4 Hz, Ar^F), 102.2 (m, arene CH), 98.2 (m, arene CH), 92.5 (m, arene CH), 38.6 (m, P*C*(CH$_3$)$_3$), 38.2 (m, P*C*(CH$_3$)$_3$), 30.5 (m, PC(*C*H$_3$)$_3$), 30.4 (m, PC(*C*H$_3$)$_3$), 23.9 (m, P*C*H$_2$), 20.7 (s, arene CH_3), 19.9 (s, arene CH_3), 18.7 (s, arene CH_3).

^{31}P{^{1}H} NMR (242.95 MHz, CD_2Cl_2, 298 K) δ 116.4 (d, $^1J_{PRh}$ = 208).

ESI-MS *m/z* found (calc.) for $C_{27}H_{52}P_2Rh$ $[M]^+$: 541.2598 (541.2594).

Elemental analysis found (calc.) for $C_{59}H_{64}BF_{24}P_2Rh$: C 50.40 (50.45); H 4.51 (4.59).

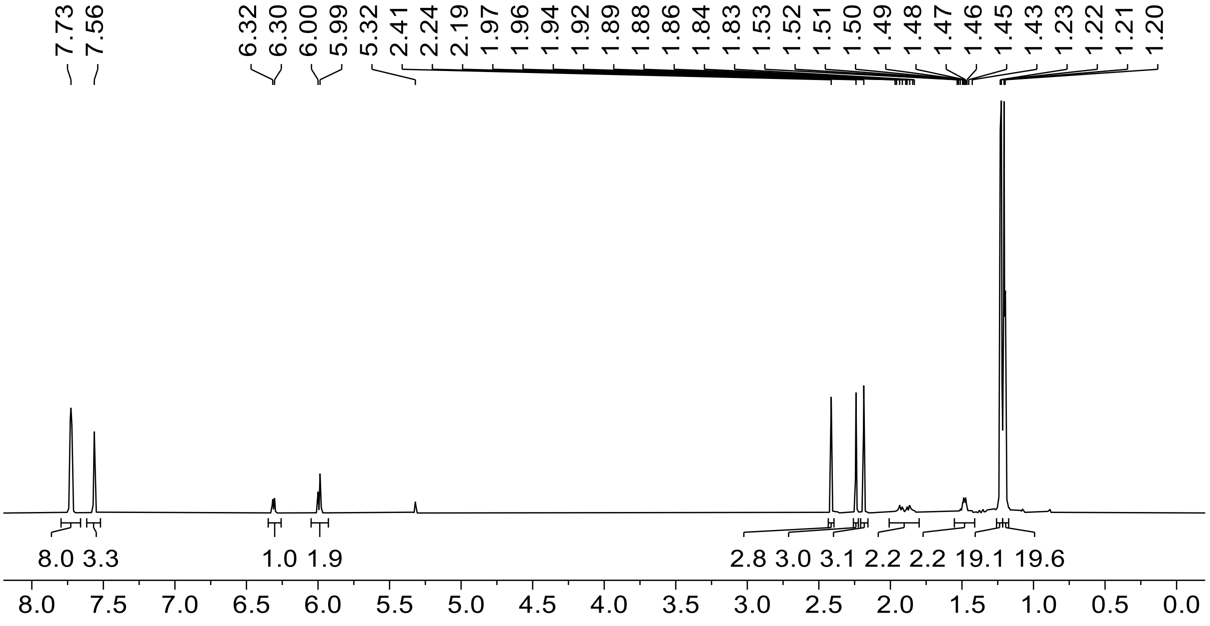

Figure S13. *^{1}H NMR spectrum of [Rh(^{t}Bu$_2$PCH$_2$CH$_2$P^{t}Bu$_2$)(C$_6$H$_3$Me$_3$)][BAr$^F{}_4$]* ***7****, (500.23 MHz, CD_2Cl_2, 298 K).*

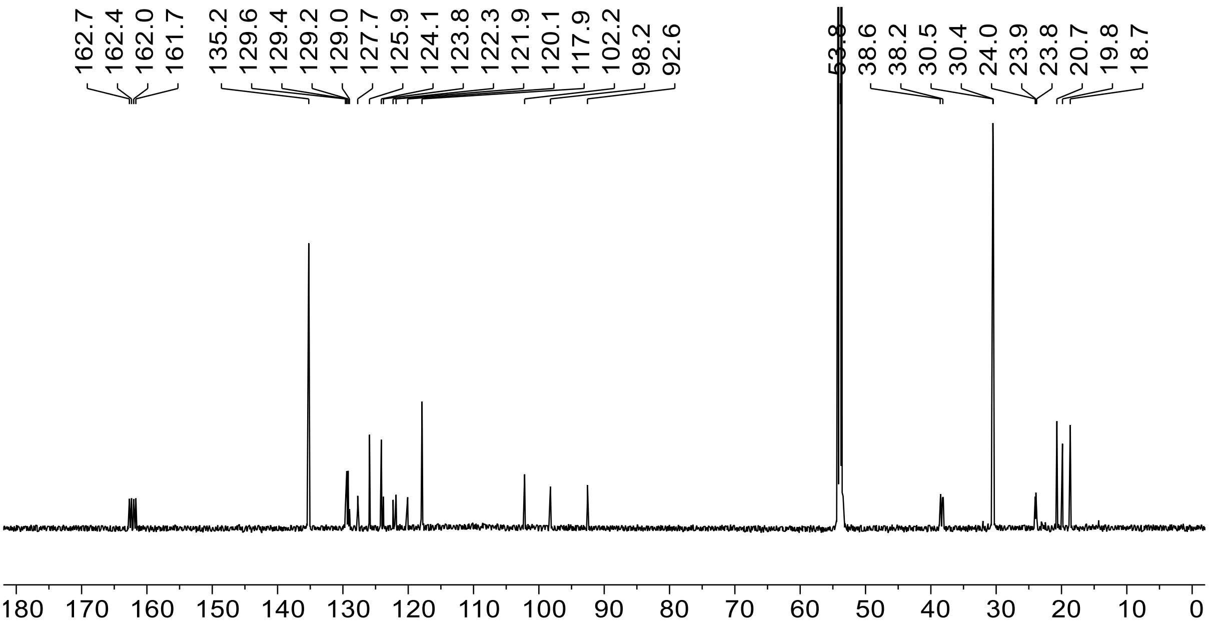

Figure S14. *$^{13}C\{^1H\}$ NMR spectrum of [Rh($^tBu_2PCH_2CH_2P^tBu_2$)($C_6H_3Me_3$)][BAr$^F{}_4$]* **7**, *(150.91 MHz, CD_2Cl_2, 298 K).*

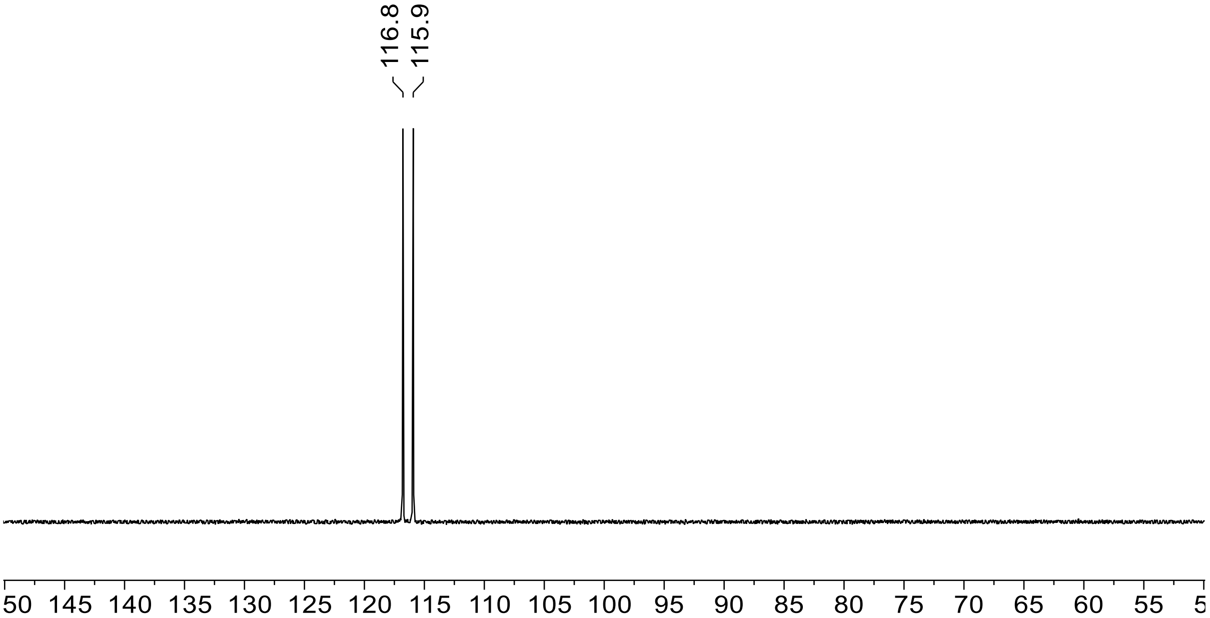

Figure S15. *$^{31}P\{^1H\}$ NMR spectrum of [Rh($^tBu_2PCH_2CH_2P^tBu_2$)($C_6H_3Me_3$)][BAr$^F{}_4$]* **7**, *(242.95 MHz, CD_2Cl_2, 298 K).*

Speciation and Post-Catalysis Experiments

Catalytic Scale Speciation

General Procedure

A 5 mm thin wall NMR tube fitted with a high vacuum PTFE (J. Young) valve containing the appropriate quantity of sieved catalyst was evacuated and backfilled with the target substrate gas (1.05-1.08 bar absolute) on a specially constructed stainless-steel vacuum/*para*-hydrogen/substrate gas NMR tube triple manifold as has been described previously.[10,11] The tube was sealed at this pressure and the headspace above the sealed tube evacuated and re-filled (4 bar absolute) with *para*-hydrogen five times on the stainless steel manifold. The manifold was isolated from the *para*-hydrogen source after a final refill to a static pressure (4 bar absolute). The valve was then opened at the NMR tube to equilibrate substrate gas and *para*-hydrogen pressures (final system pressure ~3.5 bar absolute) in the NMR tube, which was then rapidly sealed and vigorously shaken. For experiments requiring repeat cycles, the tube was returned to the gas manifold, evacuated and recharged with the same substrate gas and re-pressurised as above. When required the tube was evacuated on the high vacuum line and CD_2Cl_2 (*ca.* 0.6 mL) condensed into the tube and the tube left frozen until immediately before analysis.

Propene

Using the general procedure and $[Rh(^tBu_2PCH_2CH_2P^tBu_2)(propene)][BAr^F_4]$ **1** (2.2 mg, 1.7 µmol; 2.1 mg, 1.6 µmol; 1.8 mg, 1.4 µmol), one, three and five cycles of propene hydrogenation were conducted.

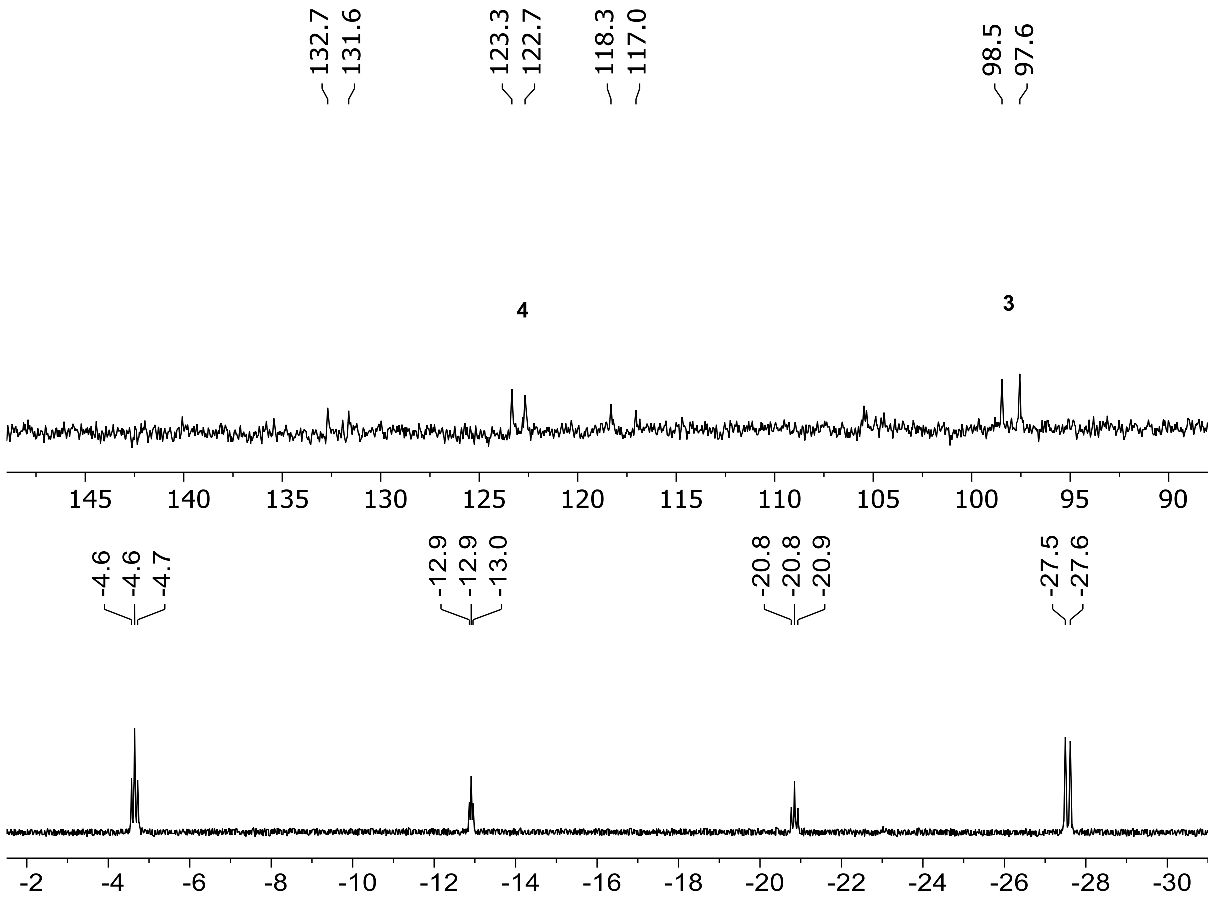

Figure S16. *$^{31}P\{^1H\}$ NMR and $^1H\{^{31}P\}$ spectra obtained after dissolution of solid materials retained after one cycle of propene hydrogenation with $[Rh(^tBu_2PCH_2CH_2P^tBu_2)(propene)][BAr^F{}_4]$* ***1*** *(161.99 MHz, CD_2Cl_2, 298 K and 400.11 MHz, CD_2Cl_2, 183 K,).*

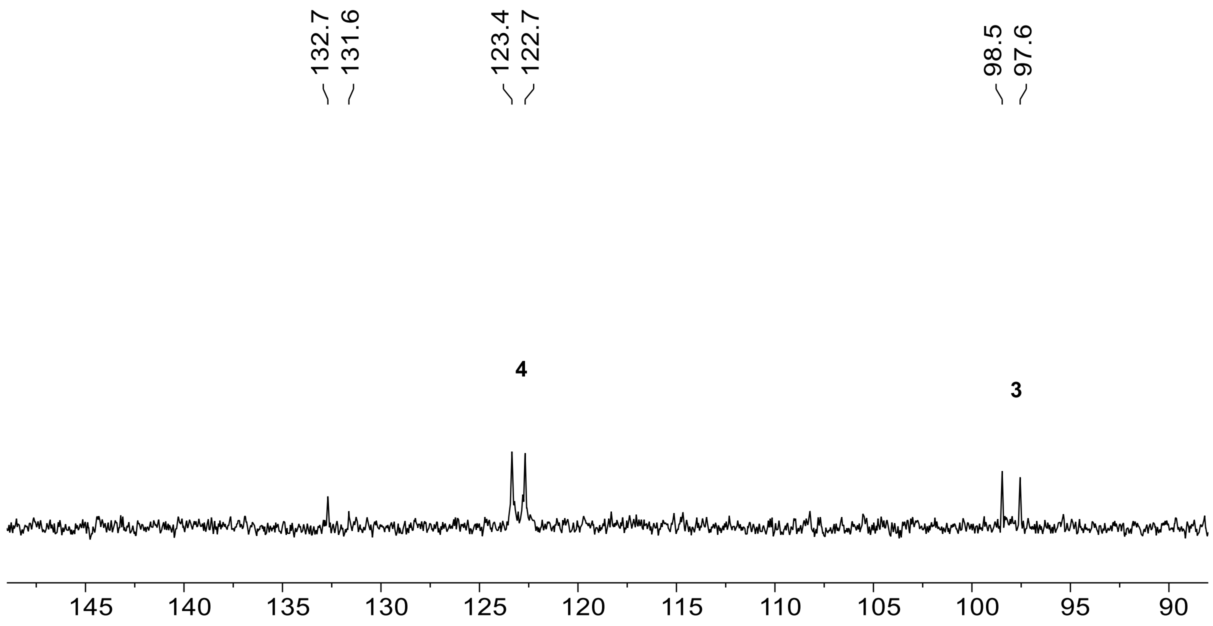

Figure S17. *$^{31}P\{^1H\}$ NMR spectrum obtained after dissolution of solid materials retained after three cycles of propene hydrogenation with $[Rh(^tBu_2PCH_2CH_2P^tBu_2)(propene)][BAr^F{}_4]$* ***1*** *(161.99 MHz, CD_2Cl_2, 298 K).*

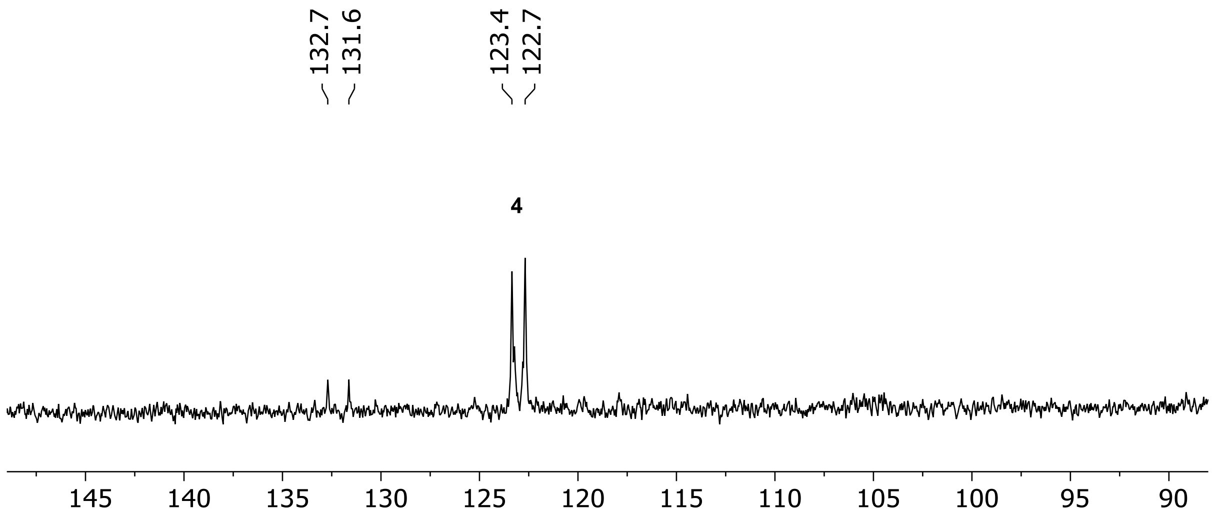

Figure S18. *$^{31}P\{^1H\}$ NMR spectrum obtained after dissolution of solid materials retained after five cycles of propene hydrogenation with [Rh($^tBu_2PCH_2CH_2P^tBu_2$)(propene)][$BAr^F{}_4$]* ***1*** *(161.99 MHz, CD_2Cl_2, 298 K).*

1-Butene

Using the general procedure and [Rh($^tBu_2PCH_2CH_2P^tBu_2$)(propene)][$BAr^F{}_4$] **1** (1.8 mg, 1.4 µmol; 1.9 mg, 1.4 µmol), one and three cycles of 1-butene hydrogenation were conducted.

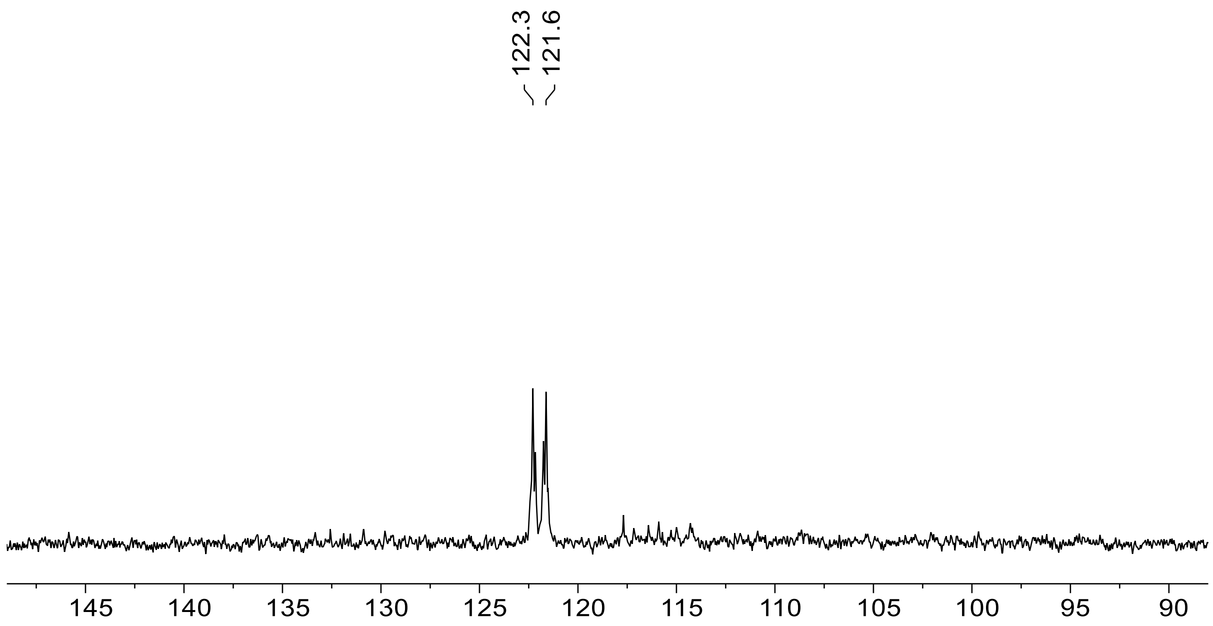

Figure S19. *$^{31}P\{^1H\}$ NMR spectrum obtained after dissolution of solid materials retained after one cycle of 1-butene hydrogenation with [Rh($^tBu_2PCH_2CH_2P^tBu_2$)(propene)][$BAr^F{}_4$]* ***1*** *(161.99 MHz, CD_2Cl_2, 183 K).*

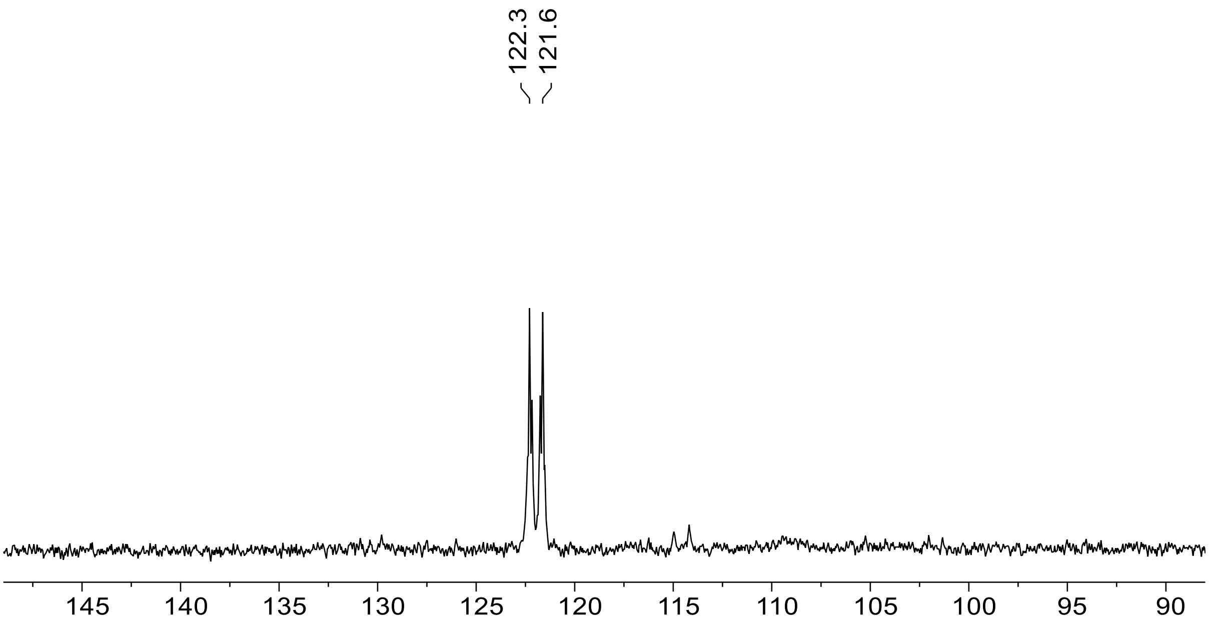

Figure S20. *$^{31}P\{^1H\}$ NMR spectrum obtained after dissolution of solid materials retained after three cycles of 1-butene hydrogenation with $[Rh(^tBu_2PCH_2CH_2P^tBu_2)(propene)][BAr^F_4]$* ***1*** *(161.99 MHz, CD_2Cl_2, 183 K).*

Propyne

Using the general procedure and $[Rh(^tBu_2PCH_2CH_2P^tBu_2)(propene)][BAr^F_4]$ **1** (2.6 mg, 2.0 µmol) one cycle of propyne hydrogenation was conducted.

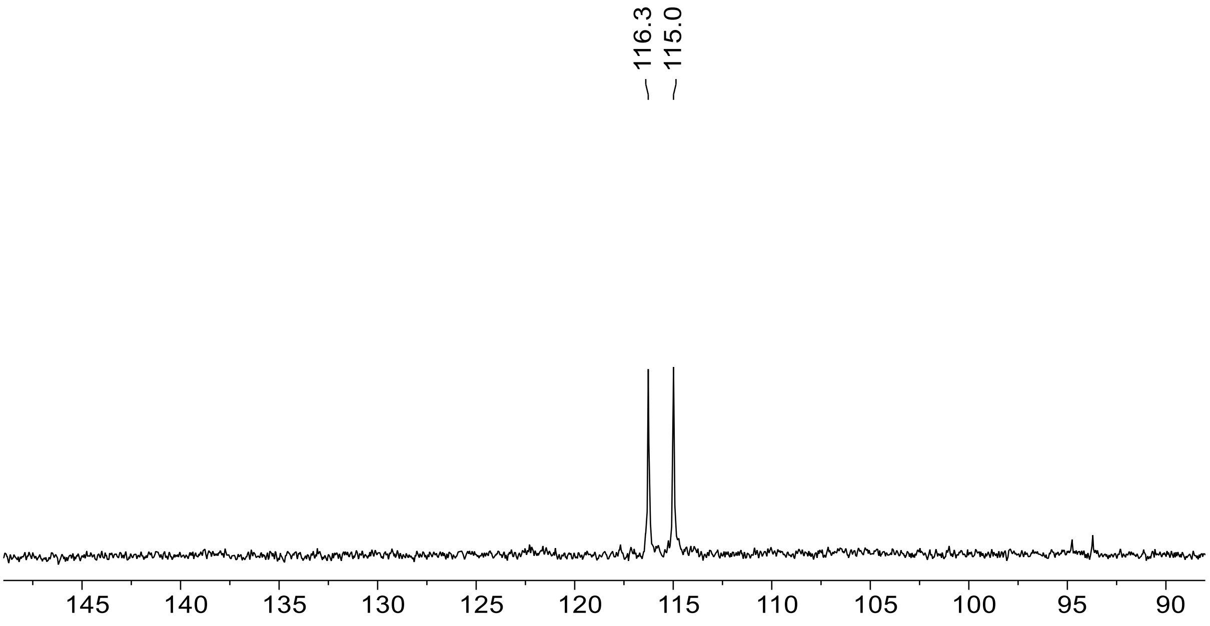

Figure S21. *$^{31}P\{^1H\}$ NMR spectrum obtained after dissolution of solid materials retained after one cycle of propyne hydrogenation with $[Rh(^tBu_2PCH_2CH_2P^tBu_2)(propene)][BAr^F_4]$* ***1*** *(161.99 MHz, CD_2Cl_2, 183 K).*

1-Butyne

Using the general procedure and $[Rh(^tBu_2PCH_2CH_2P^tBu_2)(propene)][BAr^F_4]$ **1** (2.5 mg, 1.9 µmol) one cycle of 1-butyne hydrogenation was conducted.

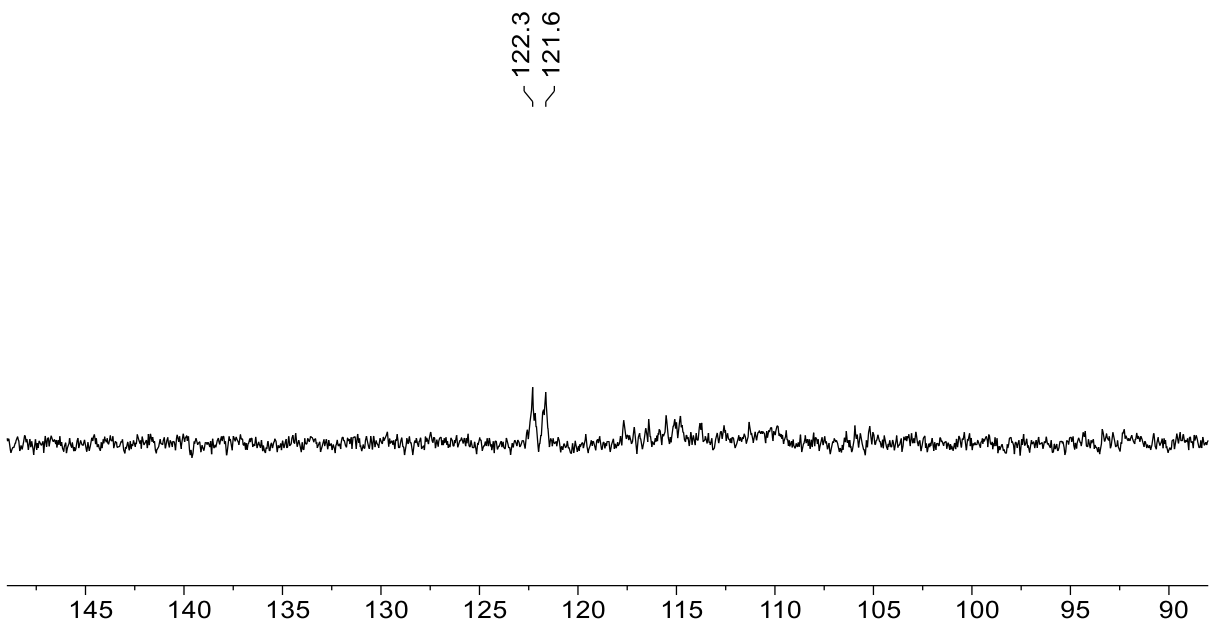

Figure S22. *$^{31}P\{^1H\}$ NMR spectrum obtained after dissolution of solid materials retained after one cycle of butyne hydrogenation with [Rh(^{t}Bu$_2$PCH$_2$CH$_2$P^{t}Bu$_2$)(propene)][BAr^F$_4$]* ***1*** *(161.99 MHz, CD_2Cl_2, 183 K).*

Para-hydrogen Addition Only

Using the general procedure without any substrate gas and [Rh(^{t}Bu$_2$PCH$_2$CH$_2$P^{t}Bu$_2$)(propene)][BAr^F$_4$] **1** (2.2 mg, 1.7 μmol) one cycle of hydrogenation was conducted for 120 seconds.

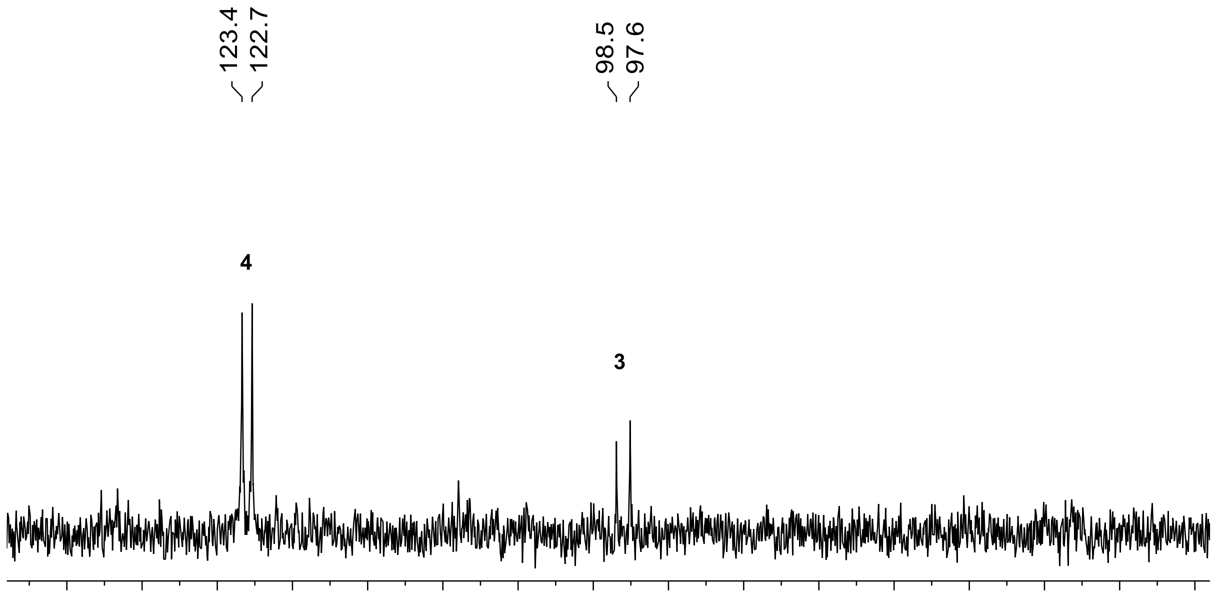

Figure S23. *$^{31}P\{^1H\}$ NMR spectrum obtained after dissolution of solid materials retained after one cycle of hydrogenation for 120 seconds with [Rh(^{t}Bu$_2$PCH$_2$CH$_2$P^{t}Bu$_2$)(propene)][BAr^F$_4$]* ***1*** *(161.99 MHz, CD_2Cl_2, 298 K).*

Using the general procedure without any substrate gas and [Rh(^{t}Bu$_2$PCH$_2$CH$_2$P^{t}Bu$_2$)(propene)][BAr^F$_4$] **1** (2.4 mg, 1.8 μmol) one cycle of hydrogenation was conducted for 10 seconds.

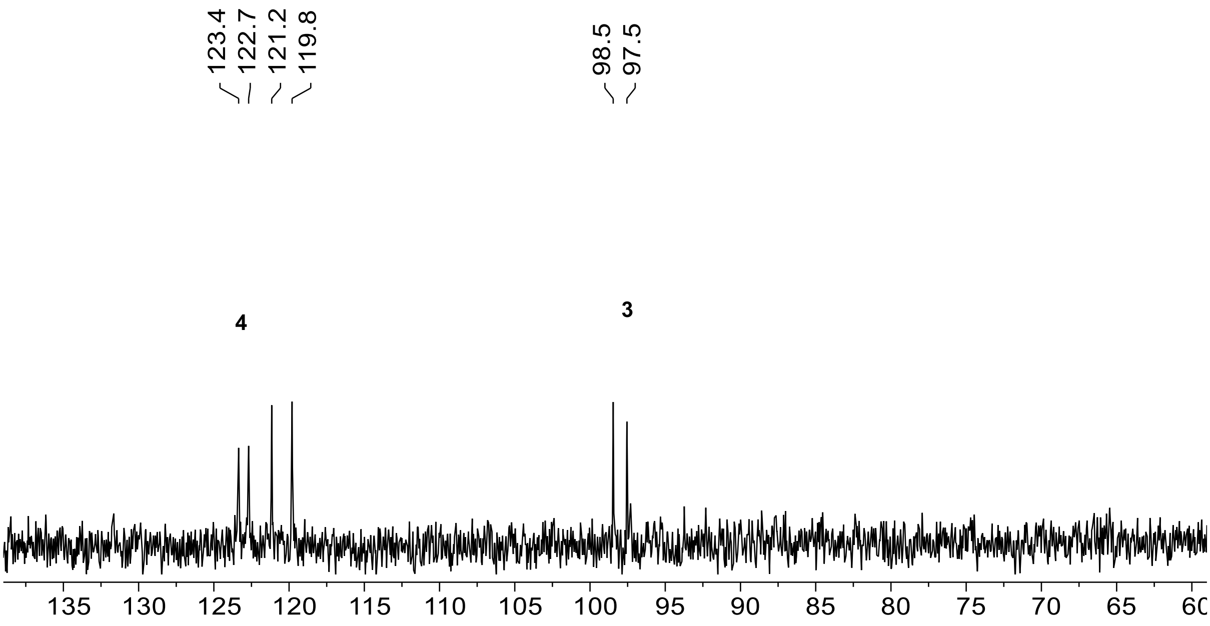

Figure S24. *$^{31}P\{^1H\}$ NMR spectrum obtained after dissolution of solid materials retained after one cycle of hydrogenation for 10 seconds with [Rh(^{t}Bu$_2$PCH$_2$CH$_2$P^{t}Bu$_2$)(propene)][BAr$^F{}_4$]* ***1*** *(161.99 MHz, CD_2Cl_2, 298 K).*

Para-Hydrogen with a Single Crystal

Using the general procedure without any substrate gas and a single crystal of [Rh(^{t}Bu$_2$PCH$_2$CH$_2$P^{t}Bu$_2$)(propene)][BAr$^F{}_4$] **1** (dimensions 0.4 mm x 0.4 mm x 0.4 mm) one cycle of hydrogenation was conducted.

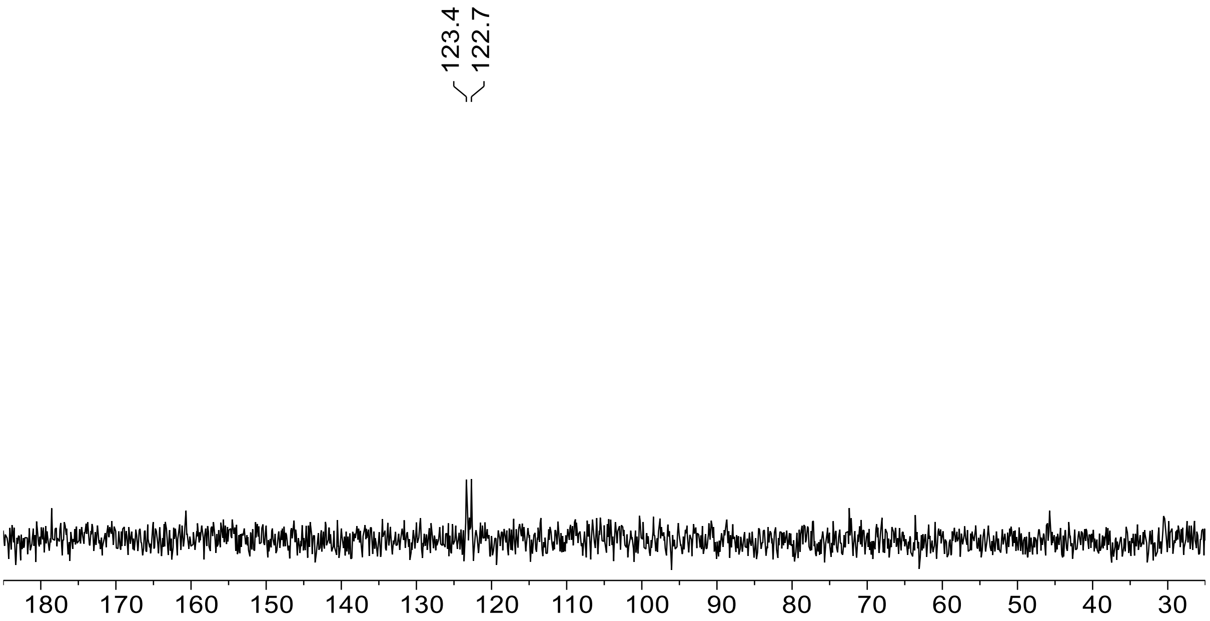

Figure S25. *$^{31}P\{^1H\}$ NMR spectrum obtained after dissolution of solid materials retained after one cycle of hydrogenation with [Rh(^{t}Bu$_2$PCH$_2$CH$_2$P^{t}Bu$_2$)(propene)][BAr$^F{}_4$]* ***1*** *(161.99 MHz, CD_2Cl_2, 298 K).*

1-Butene Addition Only

A 5 mm thin wall NMR tube fitted with a high vacuum PTFE (J. Young) valve containing [Rh(^{t}Bu$_2$PCH$_2$CH$_2$P^{t}Bu$_2$)(propene)][BAr$^F{}_4$] **1** (2.4 mg, 1.8 μmol) was evacuated and refilled with 1-butene

(1 bar absolute). The tube was stood at ambient temperature for 30 seconds before re-evacuation, CD_2Cl_2 (*ca.* 0.6 mL) condensed into the tube and the contents assayed by ^{31}P{^{1}H} and ^{1}H NMR spectroscopies at 193 K.

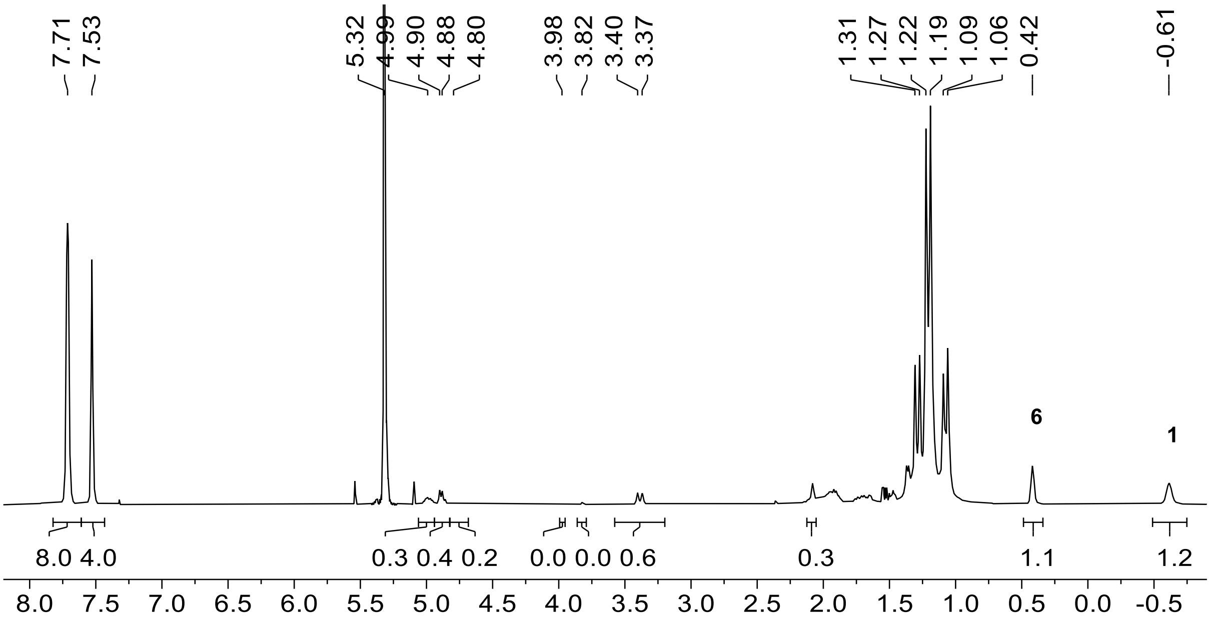

Figure S26. ^{1}H NMR spectrum recorded after addition of gaseous 1-butene to *[Rh(^{t}Bu$_2$PCH$_2$CH$_2$P^{t}Bu$_2$)(propene)][BAr$^{F}_4$]* ***1*** *for 30 seconds (400.11 MHz, CD_2Cl_2, 193 K).*

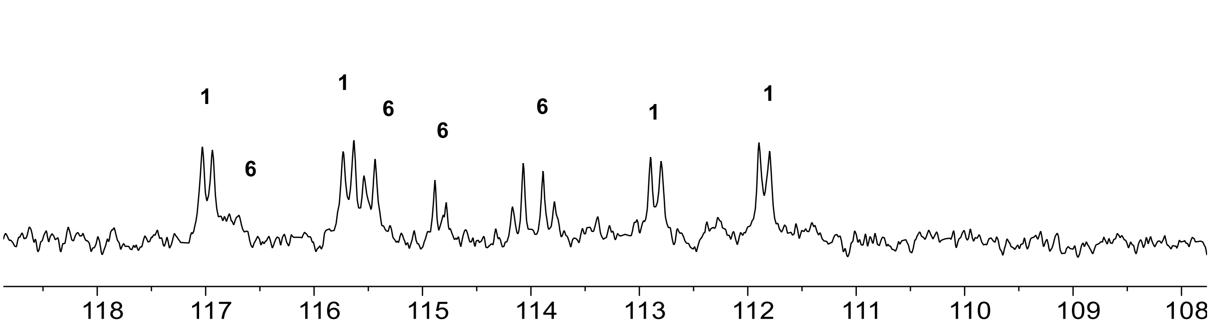

Figure S27. ^{1}H NMR spectrum recorded after addition of gaseous 1-butene to *[Rh(^{t}Bu$_2$PCH$_2$CH$_2$P^{t}Bu$_2$)(propene)][BAr$^{F}_4$]* ***1*** *for 30 seconds (161.99 MHz, CD_2Cl_2, 193 K).*

Gas Phase Monitoring of Equilibration of *p*-H_2 to *o*-H_2

A 5 mm thin wall NMR tube fitted with a high vacuum PTFE (J. Young) valve containing a single crystal of [Rh(^{t}Bu$_2$PCH$_2$CH$_2$P^{t}Bu$_2$)(propene)][BAr$^{F}_4$] **1** (dimensions ~ 0.6 mm x 0.3 mm x 0.3 mm) or [{RhH(μ-

H)(^{t}Bu$_2$PCH$_2$CH$_2$P^{t}Bu$_2$)}$_2$][BAr$^F{}_4$] **4** (dimensions ~ 0.7 mm x 0.4 mm x 0.3 mm) was evacuated and refilled with *para*-hydrogen. The tube was immediately monitored by gas-phase ^{1}H NMR spectroscopy and the growth of the integral of *ortho*-hydrogen plotted versus time as below.

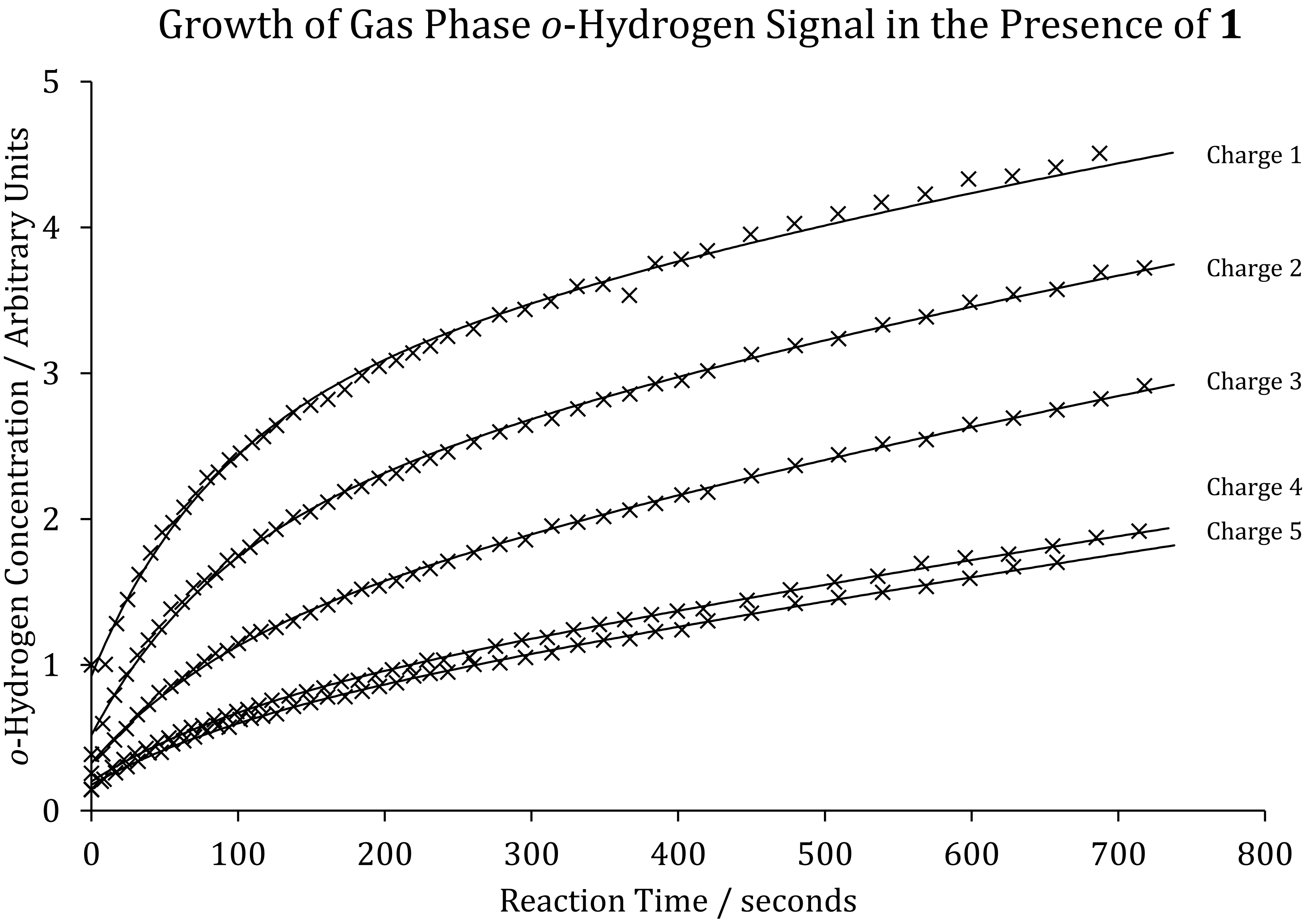
Growth of Gas Phase o-Hydrogen Signal in the Presence of 1
o-Hydrogen Concentration / Arbitrary Units
Reaction Time / seconds
0
1
2
3
4
5
0
100
200
300
400
500
600
700
800
Charge 1
Charge 2
Charge 3
Charge 4
Charge 5

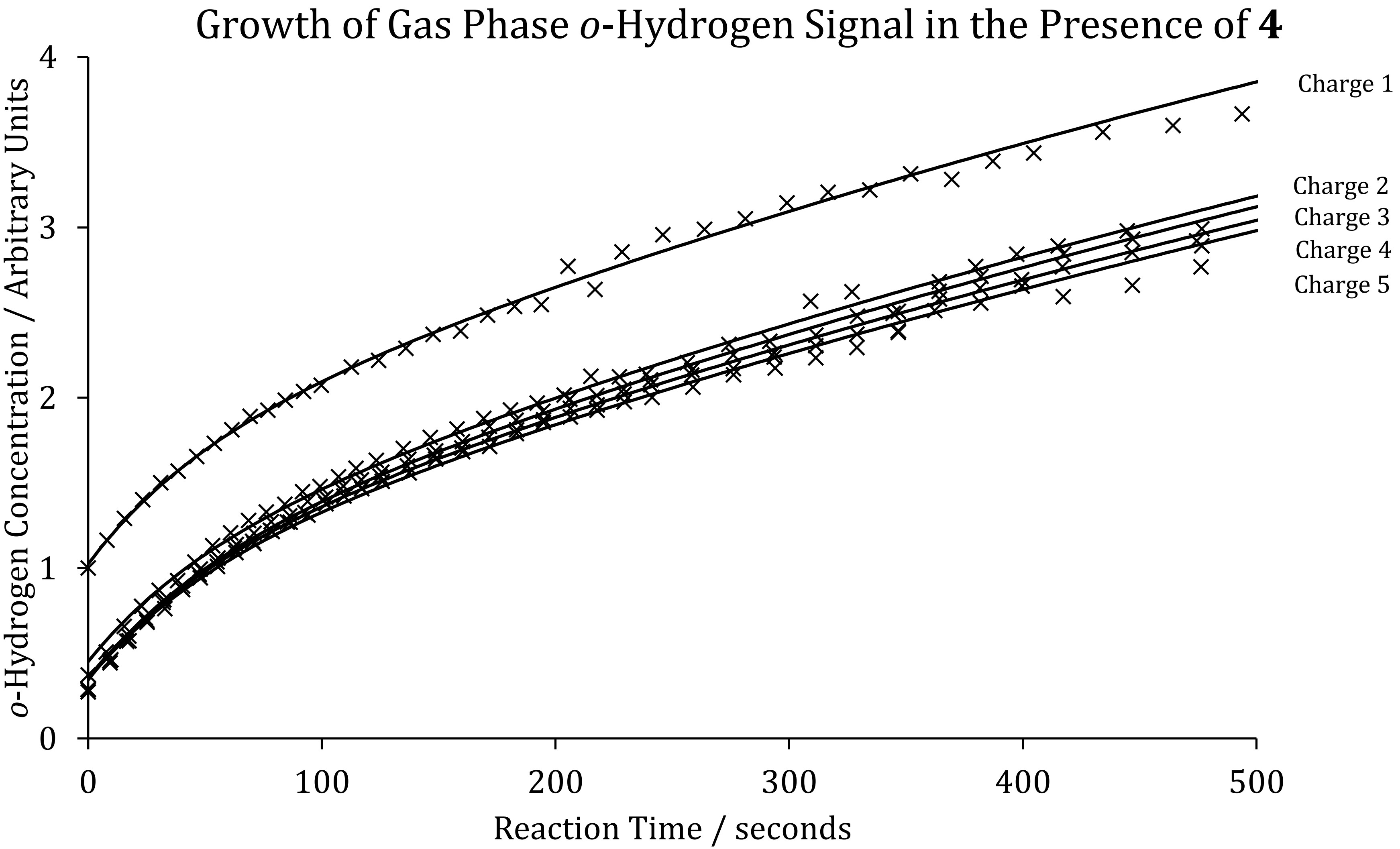
Growth of Gas Phase o-Hydrogen Signal in the Presence of 4
o-Hydrogen Concentration / Arbitrary Units
Reaction Time / seconds
0
1
2
3
4
0
100
200
300
400
500
Charge 1
Charge 2
Charge 3
Charge 4
Charge 5

Bulk Scale Analysis

Post-Catalysis Analysis of 1

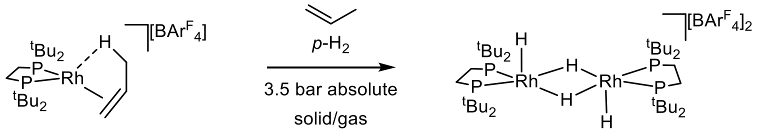

[Rh(^{t}Bu$_2$PCH$_2$CH$_2$P^{t}Bu$_2$)(propene)][BAr$^F_4$] **1**, (84.9 mg, 64.0 μmol) under propene (1 bar absolute) was pressurised with *para*-hydrogen to a total system pressure of 3.5 bar absolute and then agitated vigorously for 5 minutes to ensure gas mixing during catalysis. The vessel was evacuated, backfilled with propene (1 bar absolute), re-pressurised with *para*-hydrogen (total system pressure 3.5 bar absolute) and then vigorously mixed as previously. This process was repeated until a total of five hydrogenation cycles had been completed, whereupon the vessel was evacuated a final time, backfilled with argon and then transferred to an argon containing glovebox and packed into a solid-state rotor and 5 mm NMR tube for analysis.

^{31}P{^{1}H} NMR (CD_2Cl_2, 202.50 MHz, 183 K) δ 122.0 (dm, $^1J_{PRh}$ = 106).
^{13}C{^{1}H} CP MAS NMR (10 kHz spin rate, 100.66 MHz, 298 K) δ 164.1 (Ar^F), 136.6-119.0 (Ar^F), 39.3, 30.6, 23.9 (P*C*H$_2$).
^{31}P{^{1}H} CP MAS NMR (10 kHz spin rate, 162.04 MHz, 298 K) δ 122.4 (s br., fwhm = ~900 Hz).

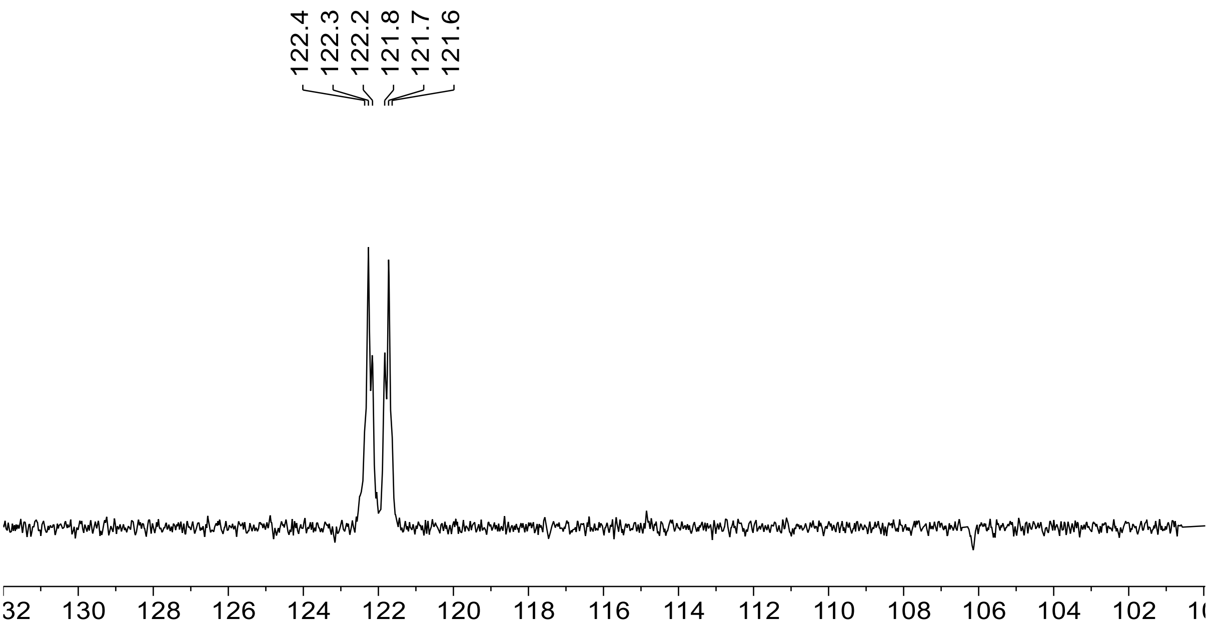

Figure S28. *^{31}P{^{1}H} NMR Spectrum of the organometallic product of [Rh(^{t}Bu$_2$PCH$_2$CH$_2$P^{t}Bu$_2$)(propene)][BAr$^F_4$]* ***1*** *subjected to five propene hydrogenation cycles (202.50 MHz, CD_2Cl_2, 183 K).*

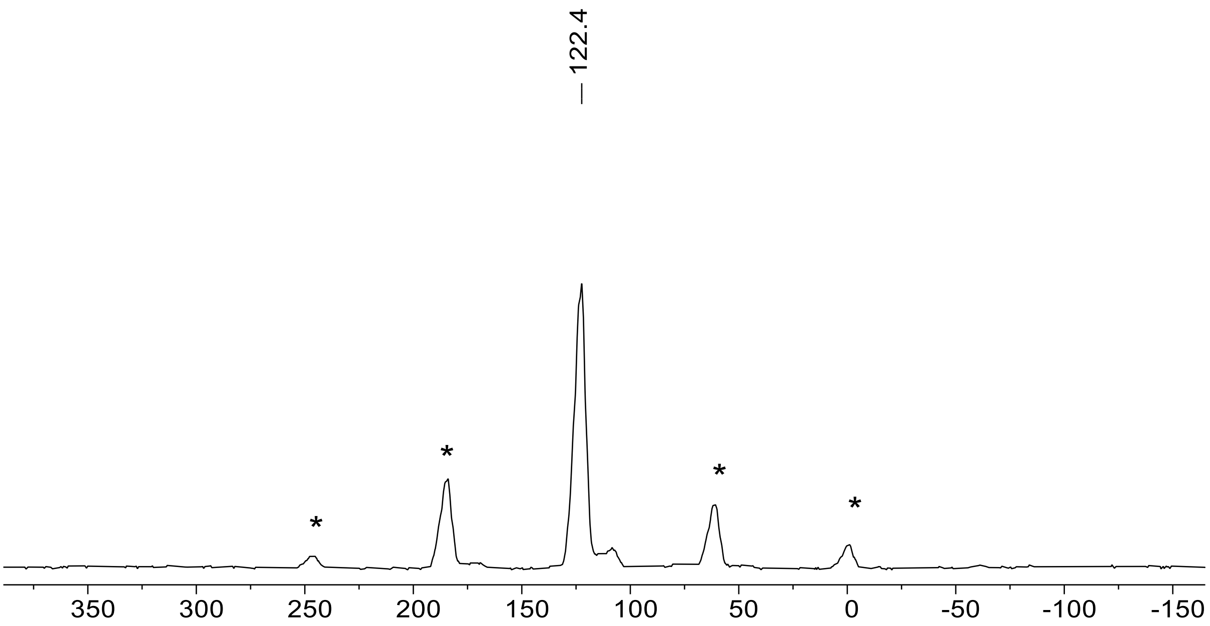

Figure S29. *$^{31}P\{^{1}H\}$ CP MAS NMR spectrum of the organometallic product of [Rh($^{t}Bu_2PCH_2CH_2P^{t}Bu_2$)(propene)][$BAr^{F}_4$]* ***1*** *subjected to five propene hydrogenation cycles (10 kHz spin rate, 162.04 MHz, 298 K). Spinning side-bands are indicated by *.*

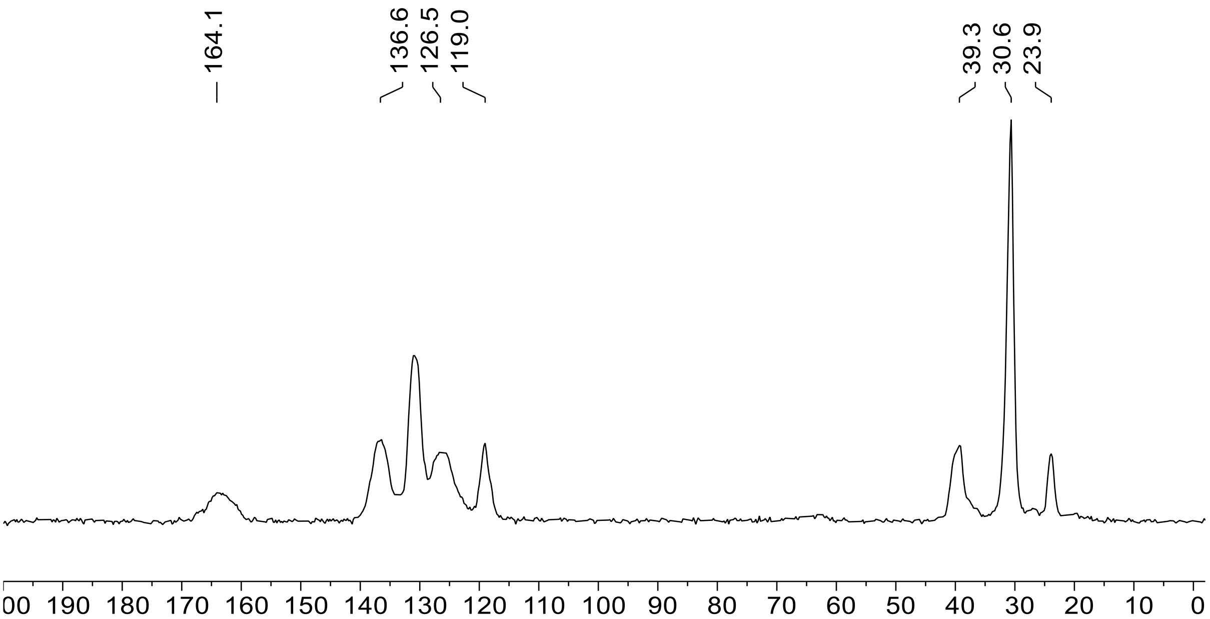

Figure S30. *$^{13}C\{^{1}H\}$ CP MAS NMR spectrum of the organometallic product of [Rh($^{t}Bu_2PCH_2CH_2P^{t}Bu_2$)(propene)][$BAr^{F}_4$]* ***1*** *subjected to five propene hydrogenation cycles (10 kHz spin rate, 100.66 MHz, 298 K).*

[Rh($^{t}Bu_2PCH_2CH_2P^{t}Bu_2$)(propene)][$BAr^{F}_4$] **1**, (59.0 mg, 44.5 μmol) under propene (1 bar absolute) was pressurised with *para*-hydrogen to a total system pressure of 3.5 bar absolute and then agitated vigorously for 2 minutes to ensure gas mixing during catalysis. The vessel was evacuated and packed into a solid-state rotor for analysis under argon.

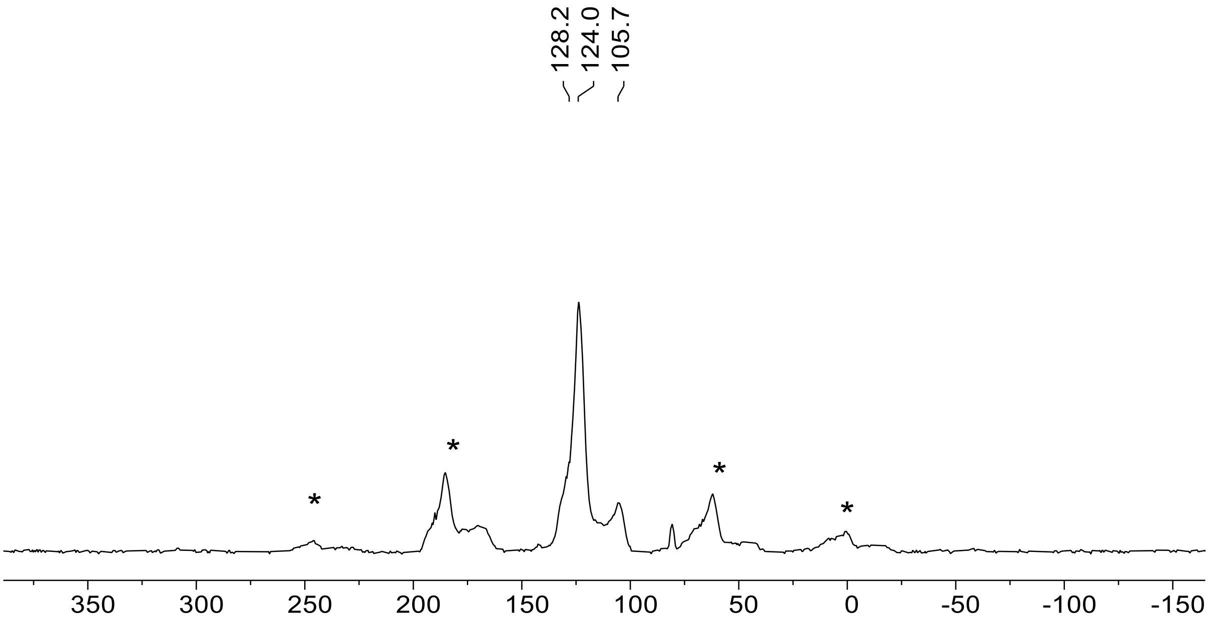

Figure S31. *$^{31}P\{^1H\}$ CP MAS NMR spectrum of the organometallic products of [Rh($^tBu_2PCH_2CH_2P^tBu_2$)(propene)][BAr$^F{}_4$] **1** subjected to one hydrogenation cycle (10 kHz spin rate, 162.04 MHz, 298 K). Spinning side-bands are indicated by *.*

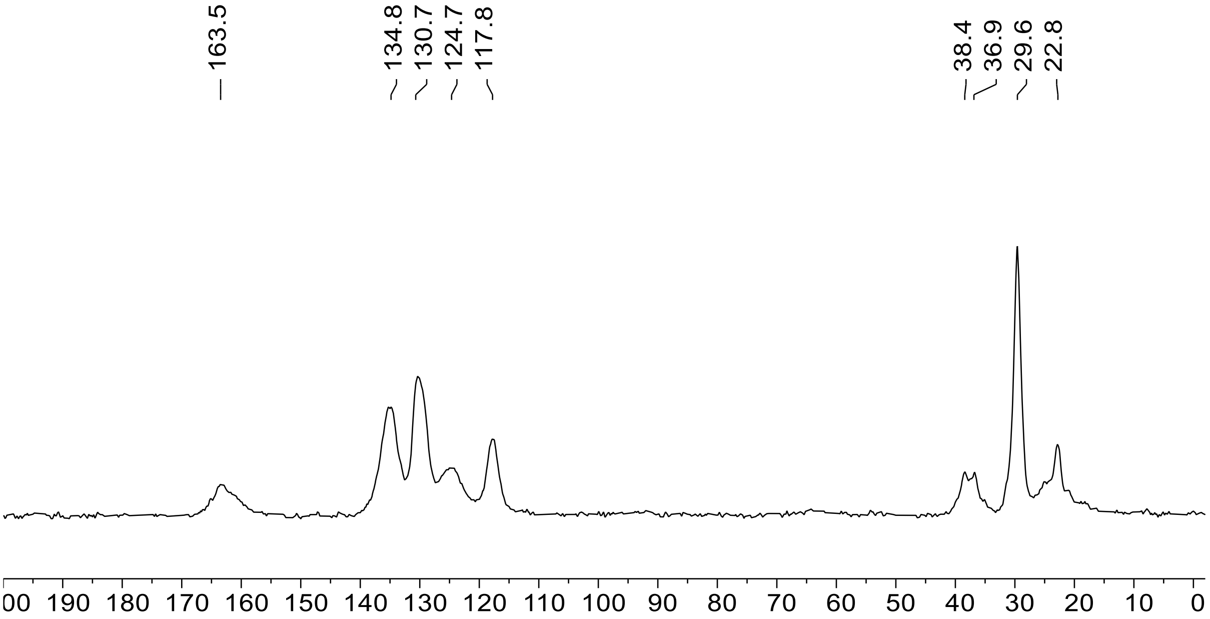

Figure S32. *$^{13}C\{^1H\}$ CP MAS NMR spectrum of the organometallic products of [Rh($^tBu_2PCH_2CH_2P^tBu_2$)(propene)][BAr$^F{}_4$] **1** subjected to one propene hydrogenation cycle (10 kHz spin rate, 100.66 MHz, 298 K).*

Post Catalysis Analysis of 4

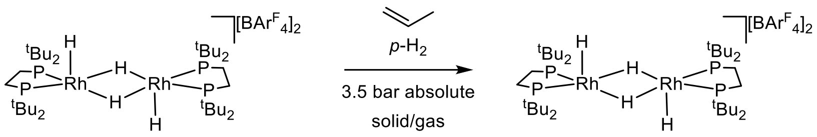

[{RhH(μ-H)(^{t}Bu$_2$PCH$_2$CH$_2$P^{t}Bu$_2$)}$_2$][BAr$^F{}_4$] **4** (91.3 mg, 35.5 μmol) under propene (1 bar absolute) was pressurised with *para*-hydrogen to a total system pressure of 3.5 bar absolute and then agitated vigorously for 5 minutes to ensure gas mixing during catalysis. The vessel was evacuated, backfilled with propene (1 bar absolute), re-pressurised with *para*-hydrogen (total system pressure 3.5 bar absolute) and then vigorously mixed as previously. This process was repeated until a total of five hydrogenation cycles had been completed, whereupon the vessel was evacuated a final time, backfilled with argon and then transferred to an argon containing glovebox and packed into a solid-state rotor for analysis.

^{31}P{^{1}H} NMR (CD_2Cl_2, 202.50 MHz, 183 K) δ 122.0 (dm, $^1J_{PRh}$ = 109).
^{13}C{^{1}H} CP MAS NMR (10 kHz spin rate, 100.66 MHz, 298 K) δ 162.4 (Ar^F), 135.8-119.2 (Ar^F), 39.7, 30.6, 24.1 (P*C*H$_2$).
^{31}P{^{1}H} CP MAS NMR (10 kHz spin rate, 162.04 MHz, 298 K) δ 123.9 (s br., fwhm = ~1000 Hz).

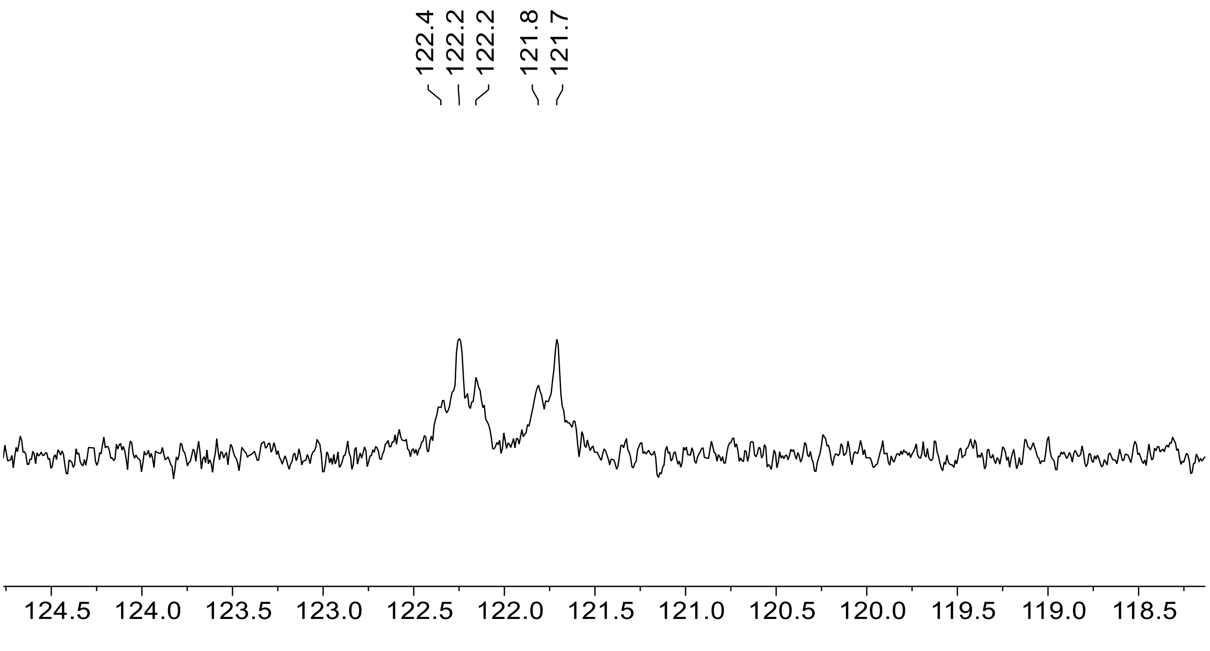

Figure S33. *^{31}P{^{1}H} NMR Spectrum of the organometallic product of [Rh(Cy$_2$PCH$_2$CH$_2$PCy$_2$)(propene)][BAr$^F{}_4$]* ***10*** *subjected to five propene hydrogenation cycles (202.50 MHz, CD$_2$Cl$_2$, 183 K).*

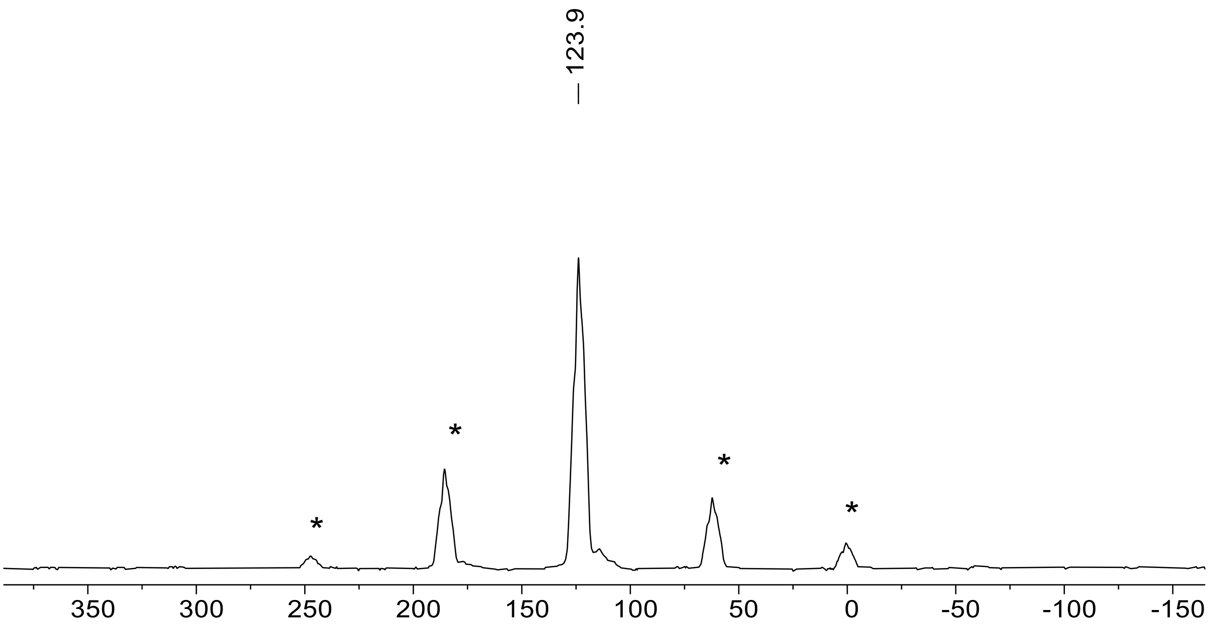

Figure S34. *$^{31}P\{^{1}H\}$ CP MAS NMR spectrum of the organometallic product of [{RhH(μ-H)($^{t}Bu_2PCH_2CH_2P^{t}Bu_2$)}$_2$][BAr$^{F}_4$]* ***4*** *subjected to five propene hydrogenation cycles (10 kHz spin rate, 162.04 MHz, 298 K). Spinning side-bands are indicated by *.*

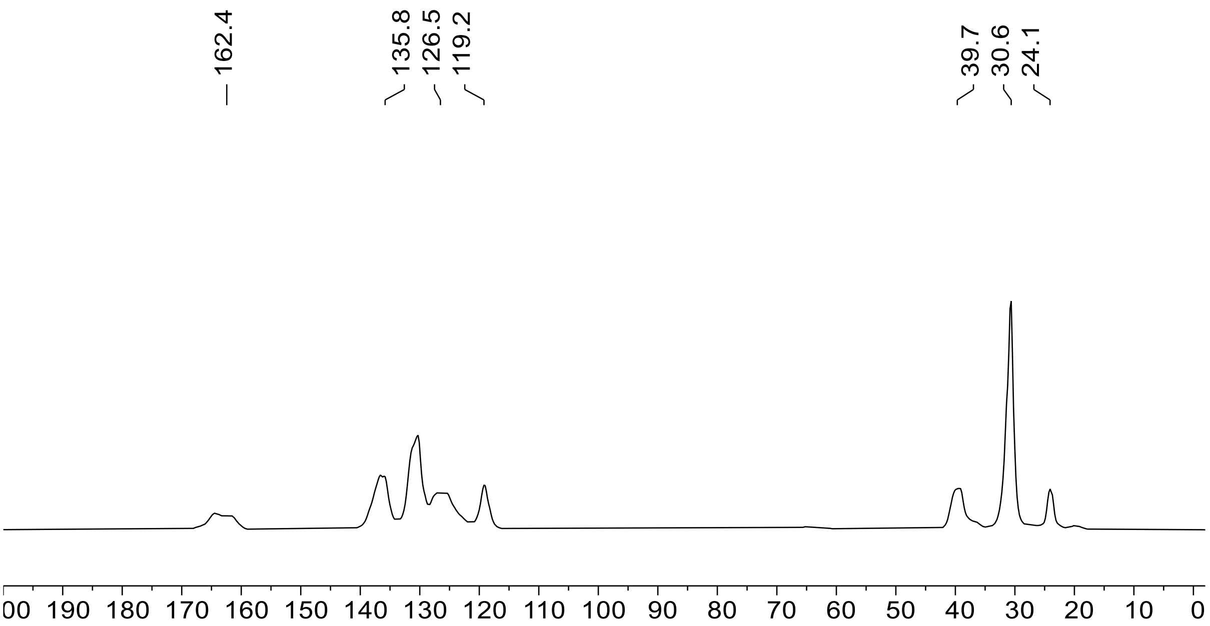

Figure S35. *$^{13}C\{^{1}H\}$ CP MAS NMR spectrum of the organometallic product of [{RhH(μ-H)($^{t}Bu_2PCH_2CH_2P^{t}Bu_2$)}$_2$][BAr$^{F}_4$]* ***4*** *subjected to five propene hydrogenation cycles (10 kHz spin rate, 100.66 MHz, 298 K).*

Post-catalysis Analysis of 10

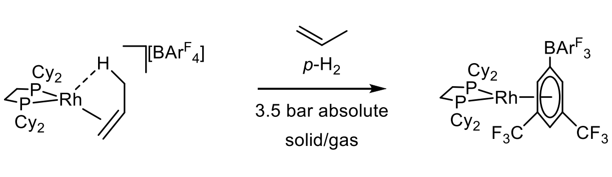

$[Rh(Cy_2PCH_2CH_2PCy_2)(propene)][BAr^F_4]$ **10**, (81.6 mg, 57.0 μmol) under propene (1 bar absolute) was pressurised with *para*-hydrogen to a total system pressure of 3.5 bar absolute and then agitated vigorously for 5 minutes to ensure gas mixing during catalysis. The vessel was evacuated, backfilled with propene (1 bar absolute), re-pressurised with *para*-hydrogen (total system pressure 3.5 bar absolute) and then vigorously mixed as previously. This process was repeated until a total of five hydrogenation cycles had been completed, whereupon the vessel was evacuated a final time, backfilled with argon and then transferred to an argon containing glovebox and packed into a 4 mm solid-state NMR rotor and a 5 mm NMR tube for analysis.

$^{31}P\{^1H\}$ NMR (CD_2Cl_2, 202.50 MHz, 203 K) δ 91.9 ($^1J_{PRh}$ = 201).
$^{13}C\{^1H\}$ CP MAS NMR (10 kHz spin rate, 100.66 MHz, 297 K) δ 163.9 (Ar^F), 137.0-91.4 (Ar^F), 39.4, 37.9, 27.8, 21.9, 17.2.
$^{31}P\{^1H\}$ CP MAS NMR (10 kHz spin rate, 162.04 MHz, 297 K) δ 91.4 (s br., fwhm = ~2100 Hz), 70.3 (s br, fwhm = ~2400 Hz).

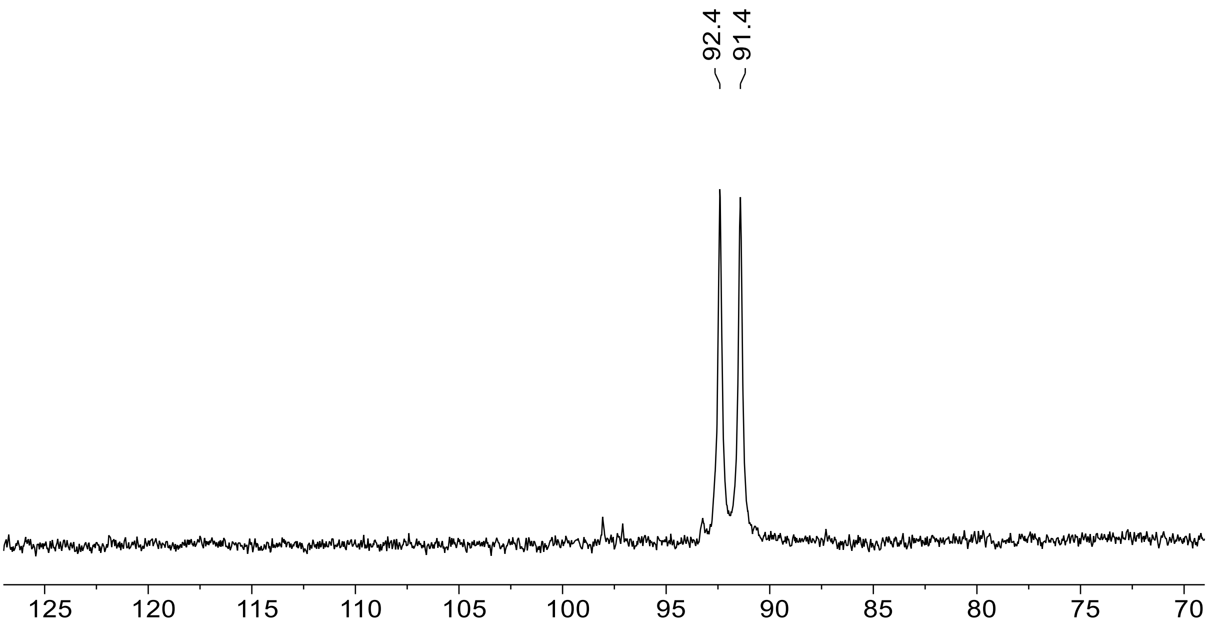

Figure S36. *$^{31}P\{^1H\}$ NMR Spectrum of the organometallic product of $[Rh(Cy_2PCH_2CH_2PCy_2)(propene)][BAr^F_4]$ **10** subjected to five propene hydrogenation cycles (202.50 MHz, CD_2Cl_2, 203 K).*

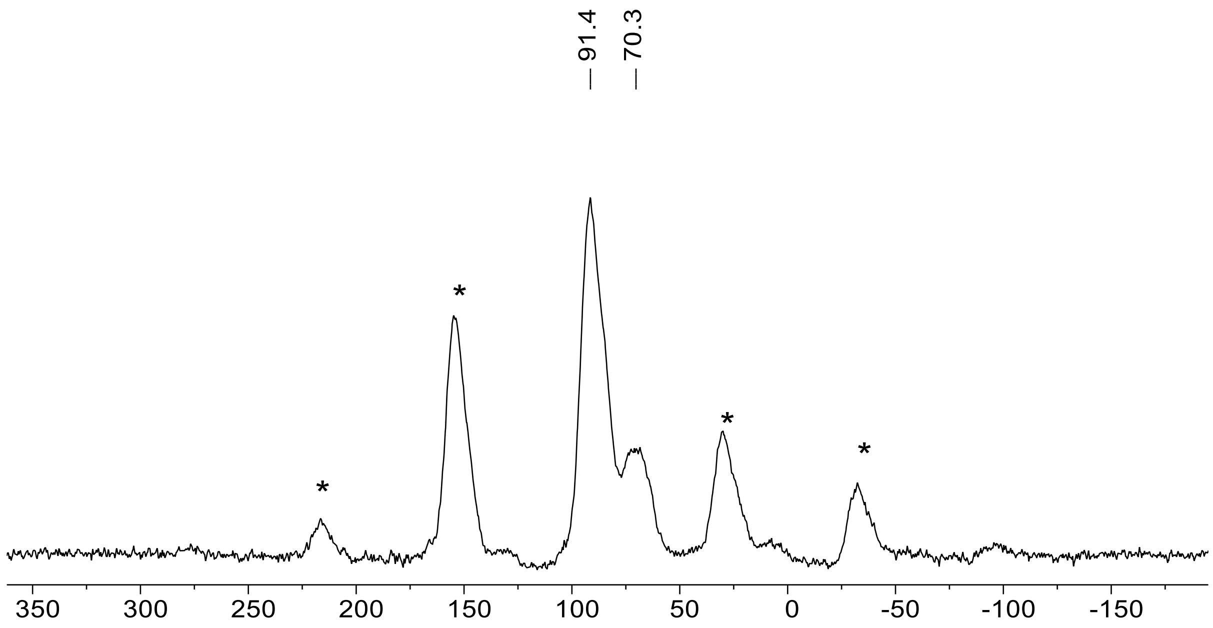

Figure S37. *$^{31}P\{^1H\}$ CP MAS NMR spectrum of the organometallic product of $[Rh(Cy_2PCH_2CH_2PCy_2)(propene)][BAr^F_4]$* ***10*** *subjected to five propene hydrogenation cycles (10 kHz spin rate, 162.04 MHz, 297 K). Spinning side-bands are indicated by *.*

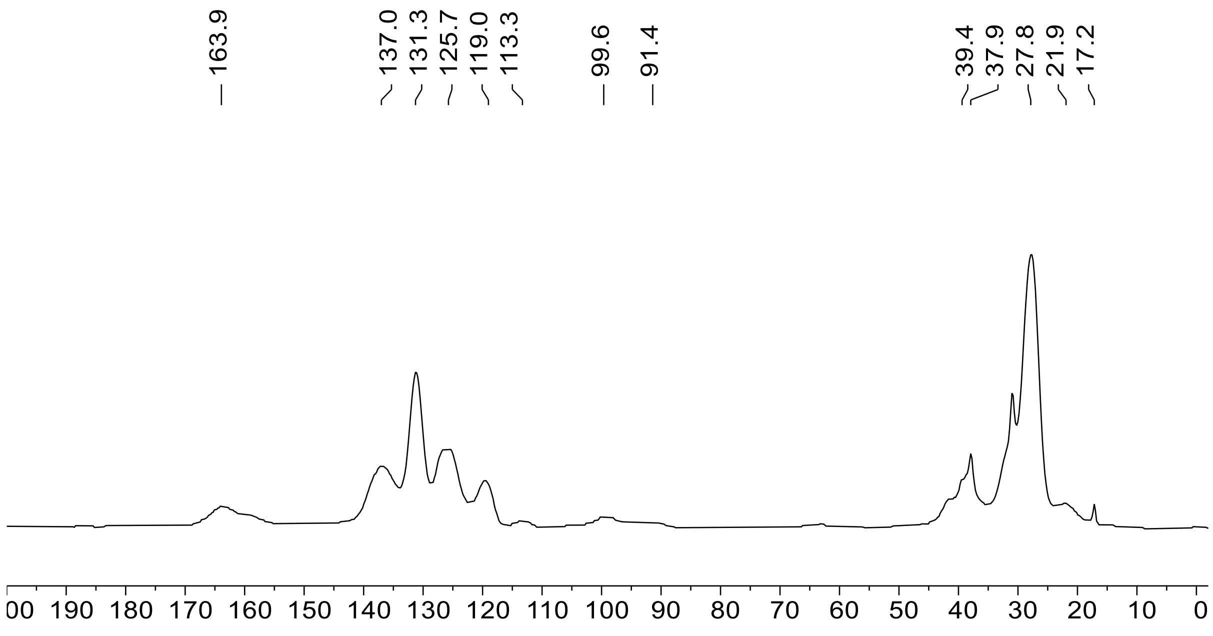

Figure S38. *$^{13}C\{^1H\}$ CP MAS NMR spectrum of the organometallic product of $[Rh(Cy_2PCH_2CH_2PCy_2)(propene)][BAr^F_4]$* ***10*** *subjected to five propene hydrogenation cycles (10 kHz spin rate, 100.66 MHz, 297 K).*

Scanning Electron Microscopy

Methodology

SEM images were taken using a JEOL JSM-7800F Prime Field Emission Scanning Electron Microscope at 1 keV using a lower electron detector (LED) at a working distance (WD) of 10 mm. Crystals for SEM analysis were mounted onto aluminium stubs using carbon coated double-sided tape and carbon coated by vapor deposition prior to imaging.

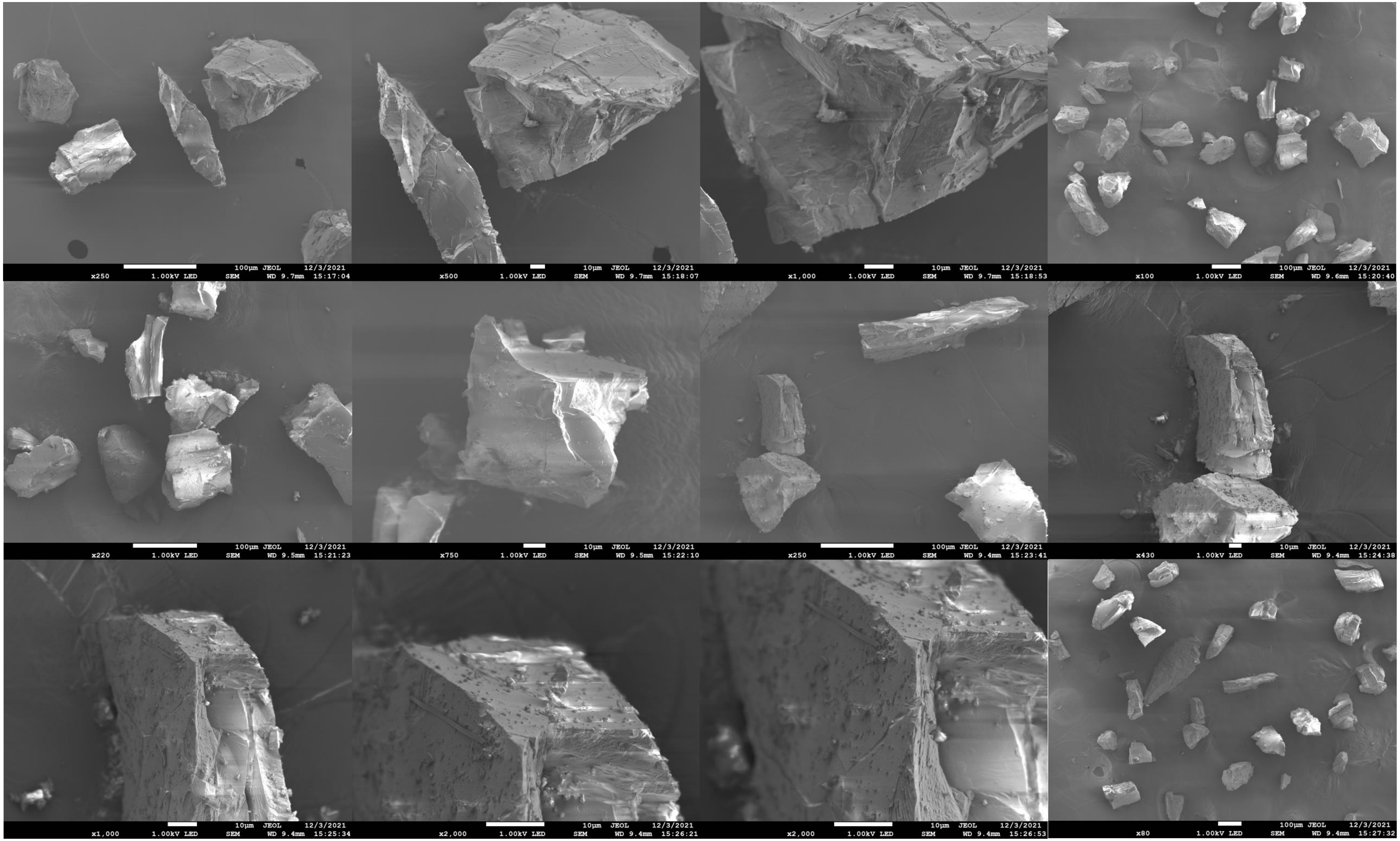

Figure S39. *SEM Images captured of samples of [Rh($^{t}Bu_2PCH_2CH_2P^{t}Bu_2$)(propene)][$BAr^{F}_4$]* ***1*** *as synthesised by solid/gas methods before PHIP catalysis.*

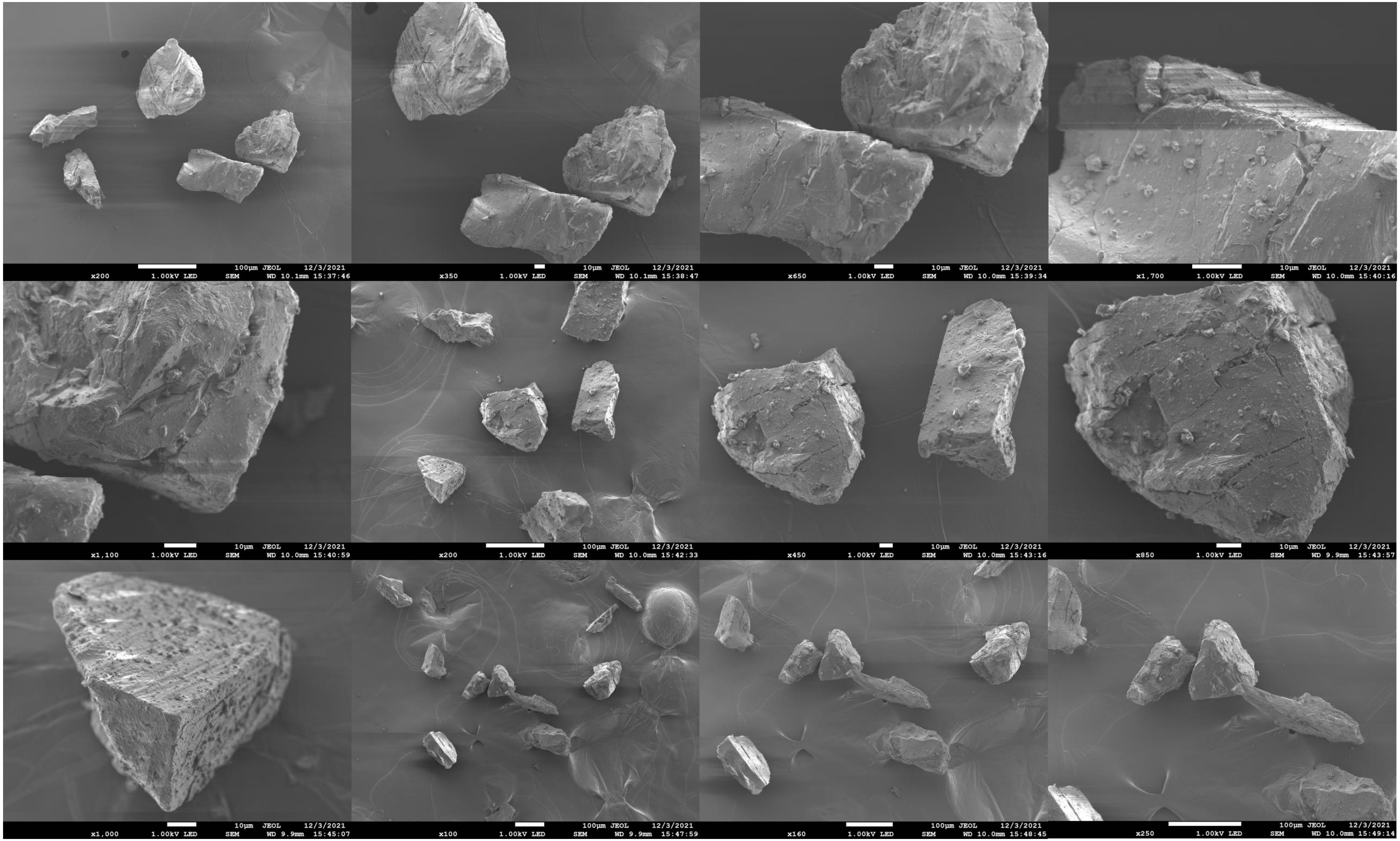

Figure S40. *SEM Images captured of samples of [Rh(^{t}Bu$_2$PCH$_2$CH$_2$P^{t}Bu$_2$)(propene)][BAr$^F{}_4$]* ***1*** *as synthesised by solid/gas methods after PHIP catalysis.*

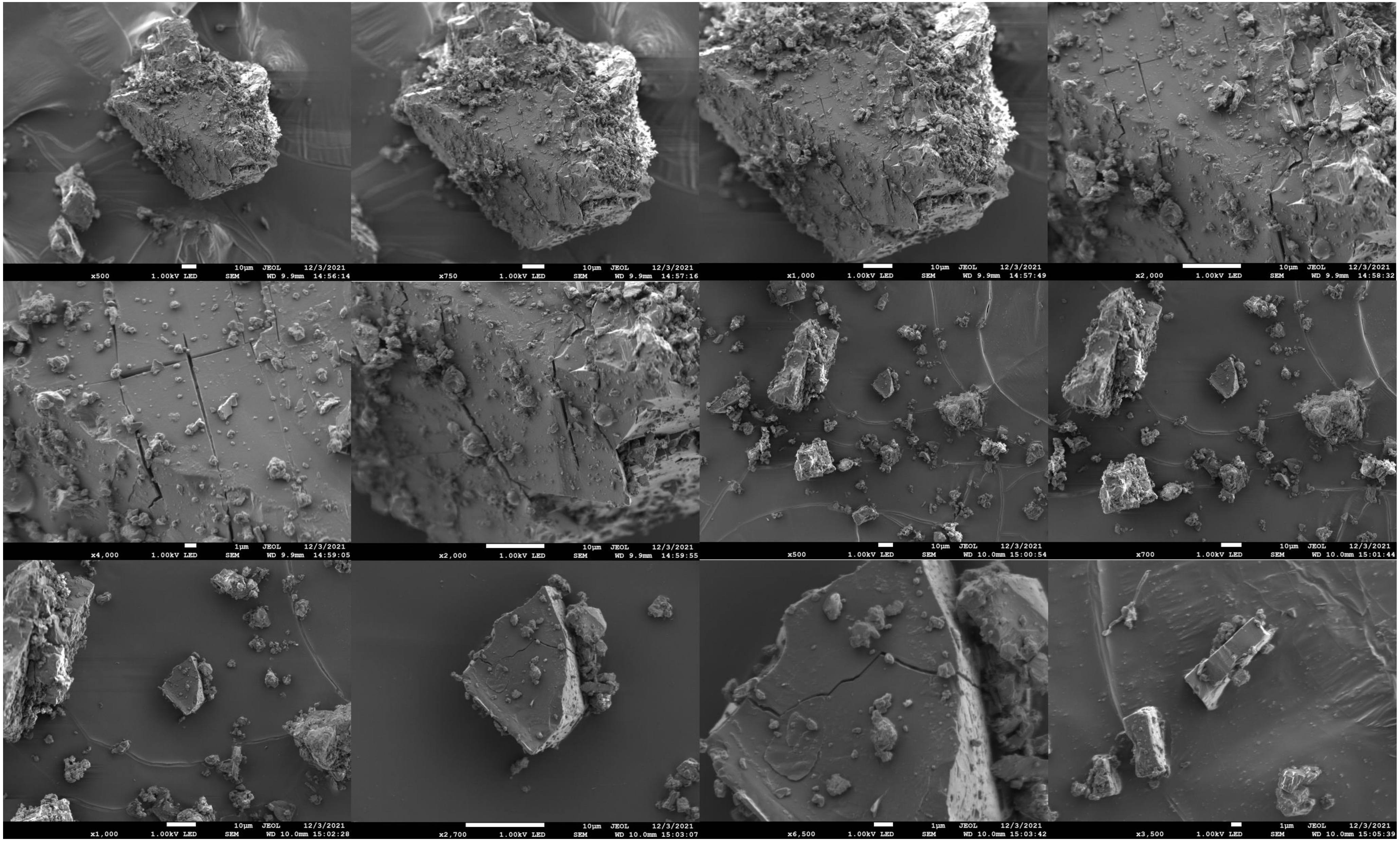

Figure S41. *SEM Images captured of samples of [Rh($Cy_2PCH_2CH_2PCy_2$)(propene)][BAr^{F}_4]* ***10*** *as synthesised by solid/gas methods before PHIP catalysis.*

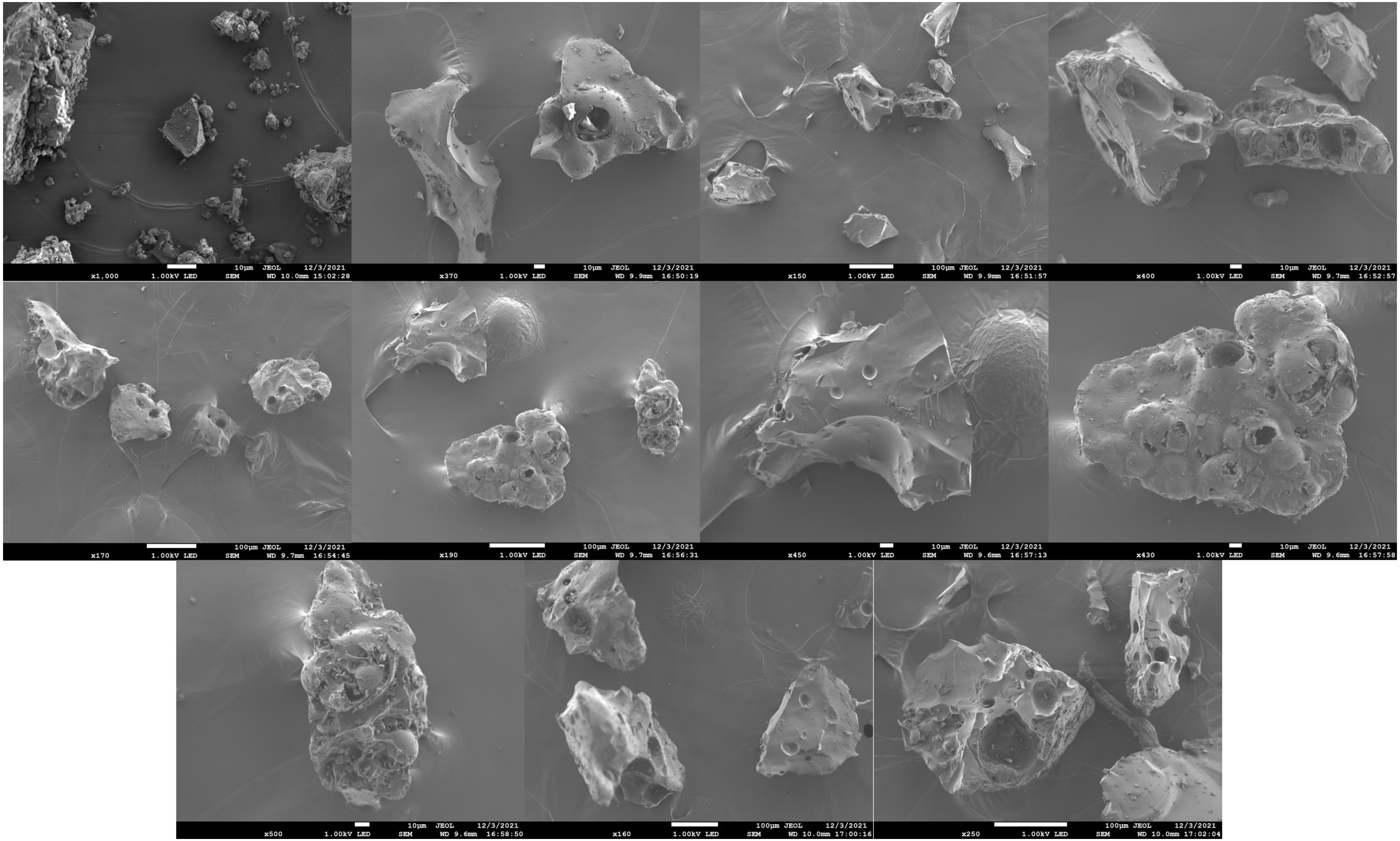

Figure S42. *SEM Images captured of samples of [Rh($Cy_2PCH_2CH_2PCy_2$)(propene)][BAr^{F}_4]* ***10*** *as synthesised by solid/gas methods after PHIP catalysis.*

X-Ray Absorption Spectroscopy

Methodology

XAS experiments were performed at beamline B18, Diamond Light Source, Oxfordshire, UK. A double-crystal Si(311) monochromator was used to scan X-ray energy from -200 to 840 eV relative to Rh K edge (23,220 eV). Each sample (sufficient that the absorption coefficient jump at the edge was satisfactory for transmission measurements) was prepared by loading finely ground powder into 3 mm OD borosilicate capillaries (wall thickness 0.01 mm, Capillary Tube Supplies Ltd, UK) in a nitrogen glovebox. These were employed for transmission XAS measurements with pure Rh metal foil measured concurrently between a second and third ionisation chamber, enabling X-ray energy calibration and data alignment.

XANES spectra were aligned by calibration of the first zero crossing point of the second derivative of the Rh foil reference spectra in each case. XAFS data processing was performed using IFEFFIT[12] with the Horae package[13] (Athena and Artemis). The amplitude reduction factor, S_0^2 was derived from EXAFS data analysis of the Rh foil reference spectrum (for which the co-ordination numbers of the face centred cubic (fcc) metal are known), yielding a value of 0.83±0.04. This was then fixed in the analysis of sample spectra. The parameters corresponding to the correction to the photoelectron energy origin, bond lengths, and mean-squared relative deviation of atoms around absorbing atoms were then varied during fitting. For the reference spectrum of independently synthesized **4** the known co-ordinations numbers (CNs) of Rh-Rh and Rh-P were assigned their known values from diffraction and an excellent fit was obtained (allowing the variation of the Rh-Rh CN also did not result in a better fit or a value significantly different to the expected CN of 1). In the fit of the sample of **1** that had mediated five cycles of propene hydrogenation, the Rh-Rh CN was permitted to vary, but was still found to be close to 1 (the Rh-P co-ordination was kept fixed to minimize the number of variables in the fit as there are two coordinating P atoms in both possible monomeric species and the dimer). In addition to obtaining a clear fit to the Rh dimer with a near identical bond length to the reference sample, attempts to fit data for this sample with nearby carbon atoms rather than a nearby Rh, as would be present in monomeric species, failed. This is expected given the contribution of the Rh-Rh at 2-3 Å, which cannot be replaced by contributions from Rh-P or Rh-C, which do not extend to these high R values. This can be seen in the plots of individual contribution paths for Rh-Rh and Rh-P that follow. All fits were performed using multiple k-weight fitting, although k^3-weighted data is shown in figures. Improved fits of the reference metal foil were obtained by fitting to higher values of R using a model accounting for multiple scattering paths (those regarded significant in FEFF) up to 5.5 Å effective path length and using the further physically reasonable assumption that all Rh-Rh interactions grow or shrink by a constant scaling factor, α relative to the known bulk Rh fcc metal crystal. Assumed relations of co-ordination number and mean-squared relative deviation are tabulated in Table S2. The upper k-limit for the range employed in each sample fit was selected on the basis of where the data became too noisy to identify clear oscillations, although nearby values of k were also checked to ensure the selection did not dramatically change the fit outcome.

Refined parameter	Sample of **1** after 5 cycles of propene/H_2	Reference sample of independently synthesized **4**	X-ray diffraction refined value
ΔE_0	-6(2)	-3(1)	
S_0^2	0.83	0.83	
$CN_{Rh\text{-}Rh}$	0.9(5)	1	
$CN_{Rh\text{-}P}$	2	2	
$R_{Rh\text{-}Rh}$	2.64(2)	2.66(2)	2.6575(5)
$R_{Rh\text{-}P}$	2.26(1)	2.281(8)	2.2809(7)<br>2.2973(8)
σ^2_{Rh}	0.005(3)	0.005(2)	
σ^2_{P}	0.0025(7)	0.0017(6)	
Goodness of Fit			
R-factor	0.040	0.011	
Reduced χ^2	13122	9382	
Fitting Range			
k-range	3.0-13.8	3.0-11.5	
R-range	1.0-3.2	1.15-3.00	
No. of independent points	14.9	9.7	
No. of fitted parameters	6	5	

Table S1. *EXAFS fitting parameters for the Rh K-Edge data on a sample of* ***1*** *that had mediated five cycles of propene hydrogenation and a reference sample of independently synthesized* ***4****. Fitting parameters:* S_0^2 *= 0.83 as determined by the use of a Rh foil standard. Parameters shown in blue were defined rather than fitted variables.*

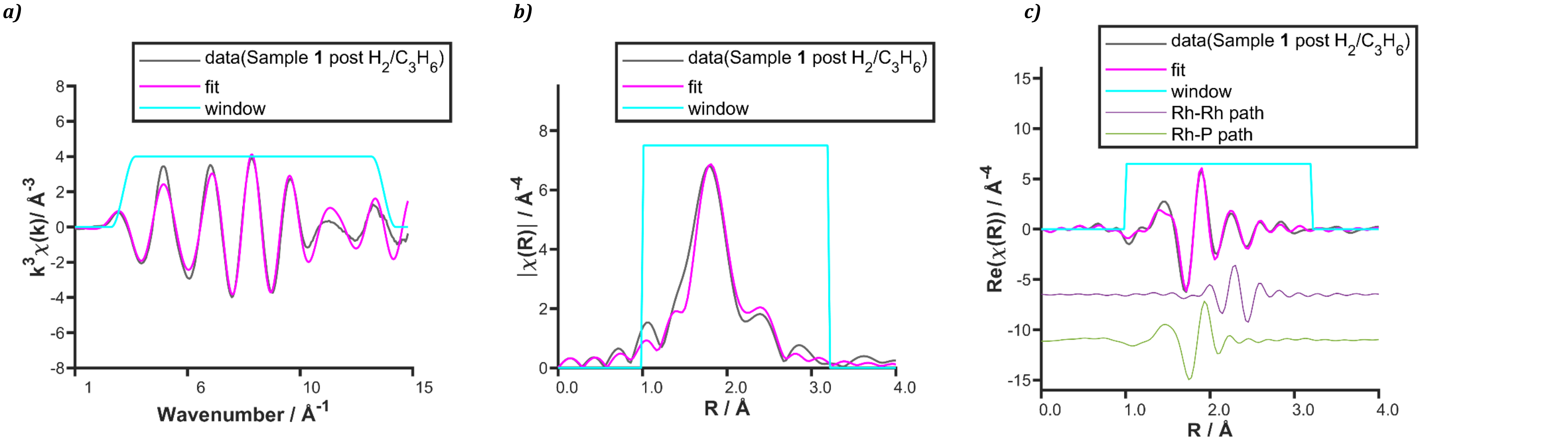

Figure S43. *Fit to Rh K-edge EXAFS spectra of sample 1 after 5 cycles of propene hydrogenation, showing (a) k-space and (b) magnitude R-space fits, along with (c) the real part of the R-space fit and component path contributions. Fit parameters and information shown in Table S1.*

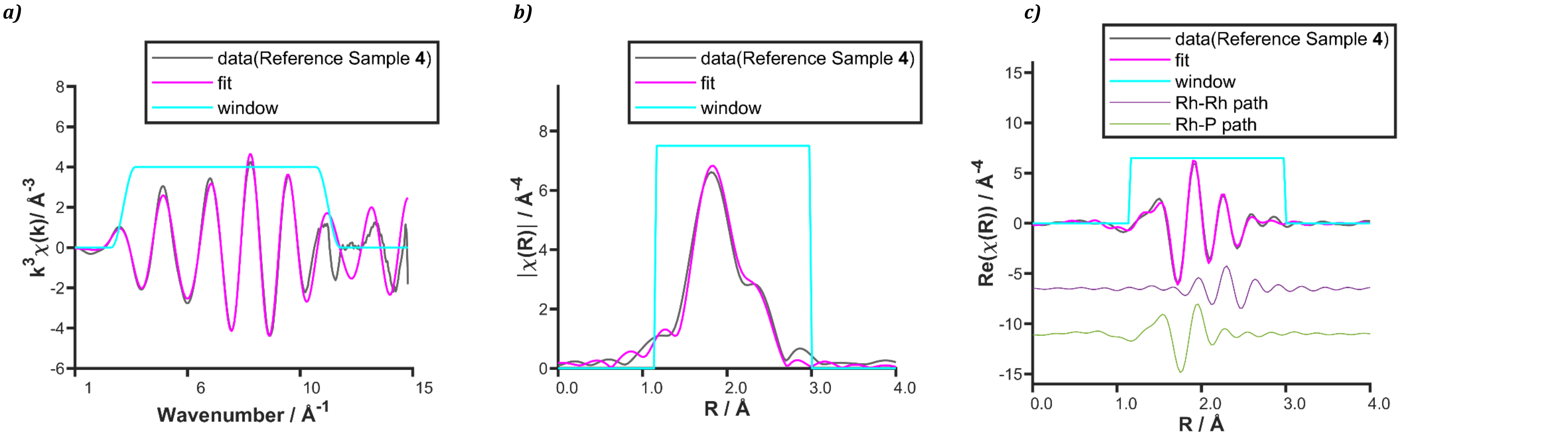

Figure S44. *Fit to Rh K-edge EXAFS spectra of a reference sample of independently synthesized 4, showing (a) k-space and (b) magnitude R-space fits, along with (c) the real part of the R-space fit and component path contributions. Fit parameters and information shown in Table S1.*

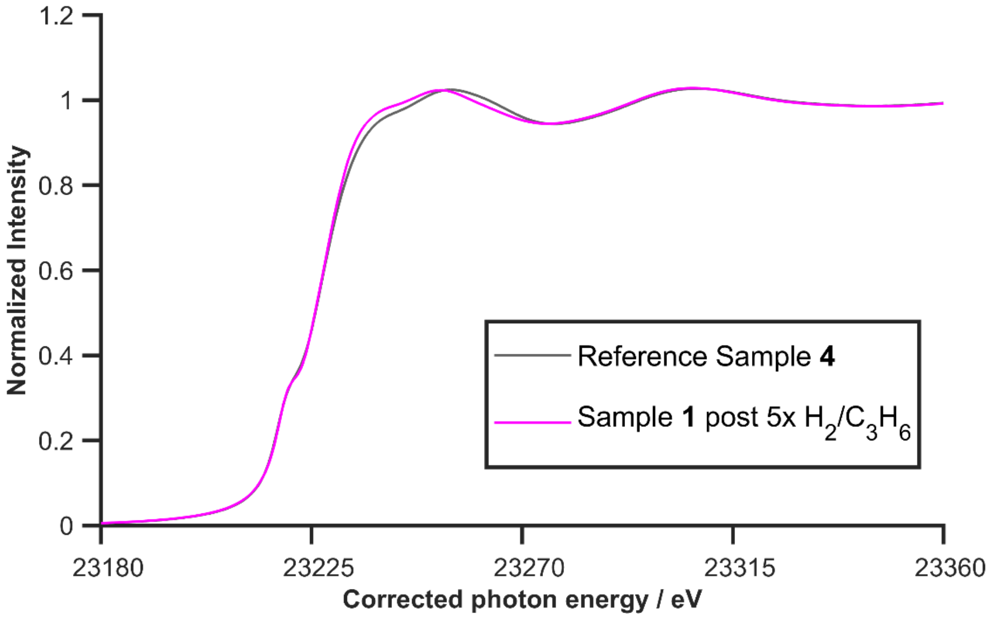

Figure S45. *Normalized XANES spectra at the Rh K-edge for a sample of 1 that had mediated five cycles of propene hydrogenation and a reference sample of independently synthesized 4. The photon energy scale has been corrected by alignment of concurrently measured Rh foil data.*

Scattering path	Co-ordination number	σ^2
Rh1 Single scattering	*12*	σ^2(Rh1)
Rh2 Single scattering	*6*	σ^2(Rh2)
Rh1Rh1 Acute triangle	*48*[a]	2 × σ^2(Rh1)
Rh1Rh2 Other double scattering	*48*	σ^2(Rh1)+ σ^2(Rh2)
Rh3 Single scattering	*24*	σ^2(Rh3)
Rh1Rh1 Obtuse triangle	*48*[a]	2 × σ^2(Rh1)
Rh1Rh3 Obtuse triangle	*96*	σ^2(Rh1)+ σ^2(Rh3)
Rh4 Single scattering	*12*	σ^2(Rh4)
Rh1Rh1 Non-forward linear	*12*[a]	2 × σ^2(Rh1)
Rh1Rh4 Forward scattering	*24*	σ^2(Rh4)
Rh1 Forward through absorber	*12*	2 × σ^2(Rh1)
Rh1Rh4Rh1 Double forward scattering	*12*	σ^2(Rh4)

Table S2. *Assumed relations of co-ordination number and mean-squared relative deviation used in extended Rh foil fit (based on fcc structure).* [a]*To minimise the number of independent parameters the approximation is made that* $CN_{Rh1xRh1}$ *can be used when fitting all three of these paths involving two nearest shell Rh scatterers and the 4:4:1 ratio maintained.*

refined parameter	Rh foil sample
ΔE_0	3.1(4)
S_0^2	0.83(4)
α	-0.0122(6)
σ^2(Rh1)	0.0036(3)
σ^2(Rh2)	0.006(1)
σ^2(Rh3)	0.0057(6)
σ^2(Rh4)	0.0058(6)
goodness of fit	
R-factor	0.014
Reduced χ^2	717
fitting range	
k-range	3.0-14.5
R-range	1.0-5.5
No. of independent points	32.7
No. of fitted parameters	7

Table S3. *EXAFS fitting parameters for the Rh K-Edge data on Rh foil, were α is the scaling factor applied to each path length relative to the reported crystallographic bond lengths in bulk fcc Rh.*

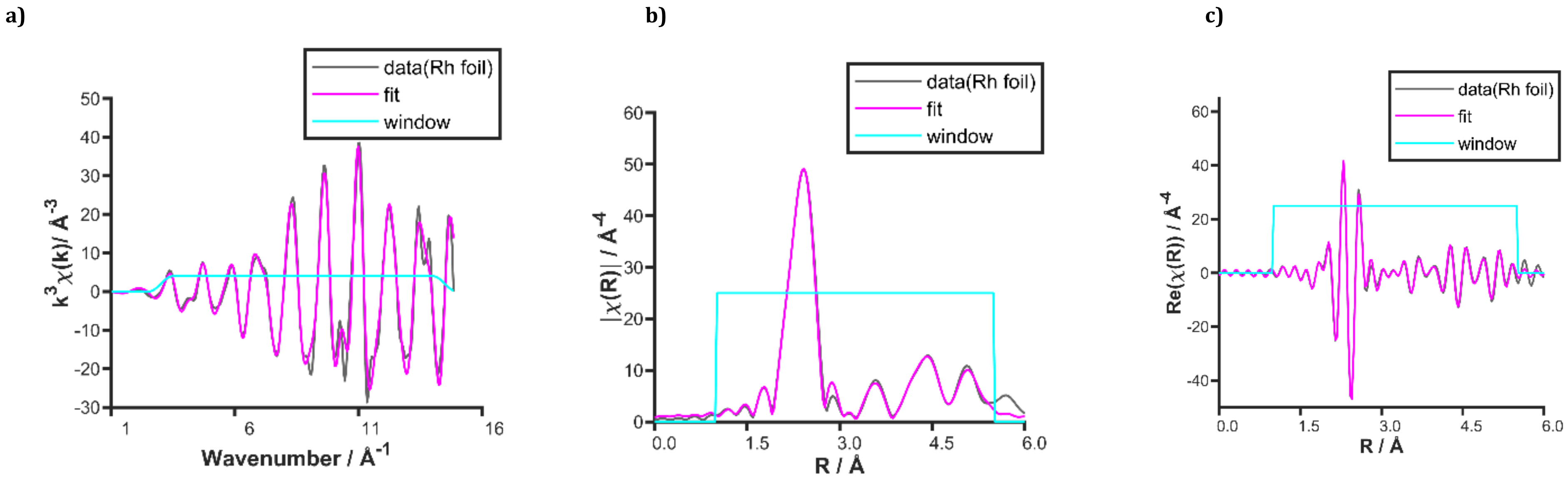

Figure S46. *Fit to Rh K-edge EXAFS spectra of a Rh foil sample, showing (a) k-space and (b) magnitude R-space fits, along with (c) the real part of the R-space fit. Fit parameters and information shown in Table S3.*

Gas-phase NMR of 1-Butyne to 1,3-Butadiene Isomerisation

A sample of [Rh(^{t}Bu$_2$PCH$_2$CH$_2$P^{t}Bu$_2$)(nbd)][BAr^F$_4$] (8.8 mg, 6.4 µmol) in an NMR tube was hydrogenated (2 bara absolute) for 90 minutes before the tube was evacuated and the placed under an atmosphere of propene (2 bar absolute) for 120 minutes. The headspace was evacuated again and the tube backfilled with 1-butyne (1 bar absolute) and the headspace monitored by gas phase NMR spectroscopy for 30 hours, wherein a slow consumption of gaseous 1-butyne and appearance of signals attributed to 1,3-butadiene was observed.

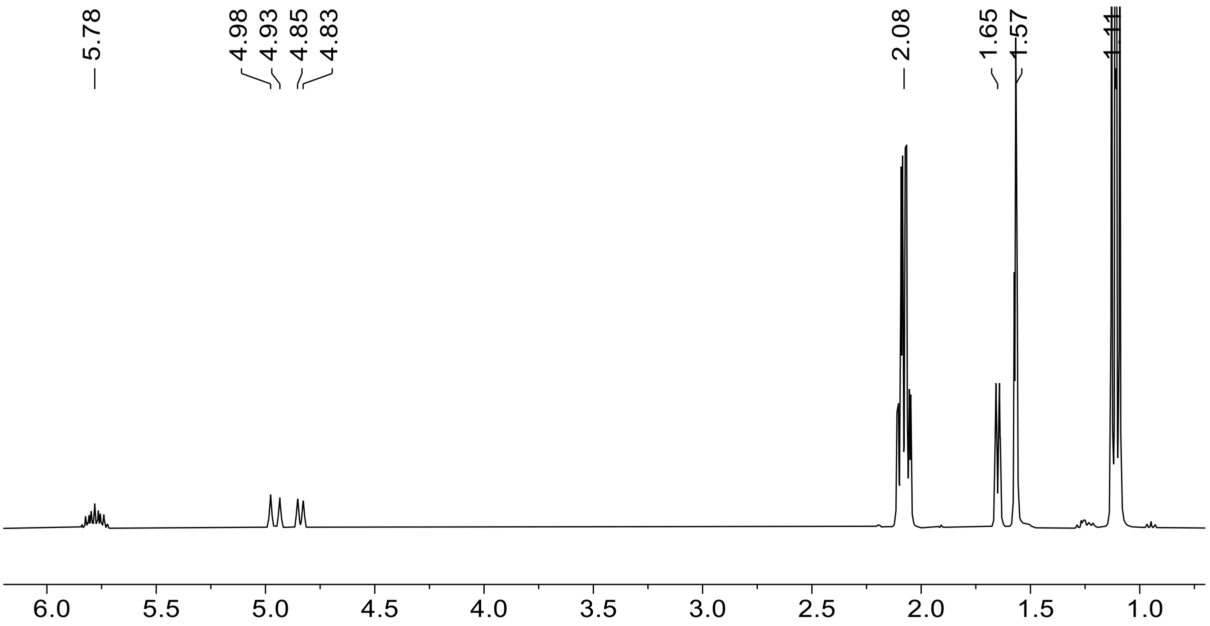

Figure S47. *Gas-phase ^{1}H NMR spectrum of a mixture of 1-butyne and propene recorded immediately after addition of 1-butyne to [Rh(^{t}Bu$_2$PCH$_2$CH$_2$P^{t}Bu$_2$)(propene)][BAr^F$_4$]* ***1*** *(298 K).*

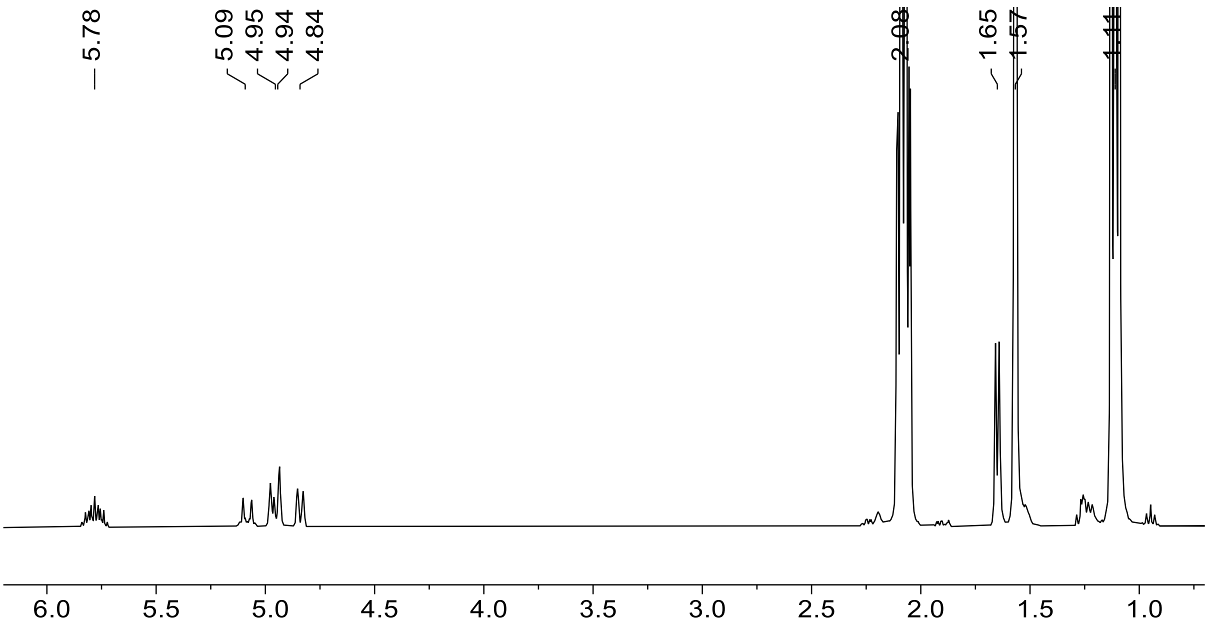

Figure S48. *Gas-phase ^1H NMR spectrum of a mixture of 1-butyne, 1,3-butadiene and propene recorded 30 hours after addition of 1-butyne to [Rh($^tBu_2PCH_2CH_2P^tBu_2$)(propene)][BAr^F_4]* ***1*** *(298 K).*

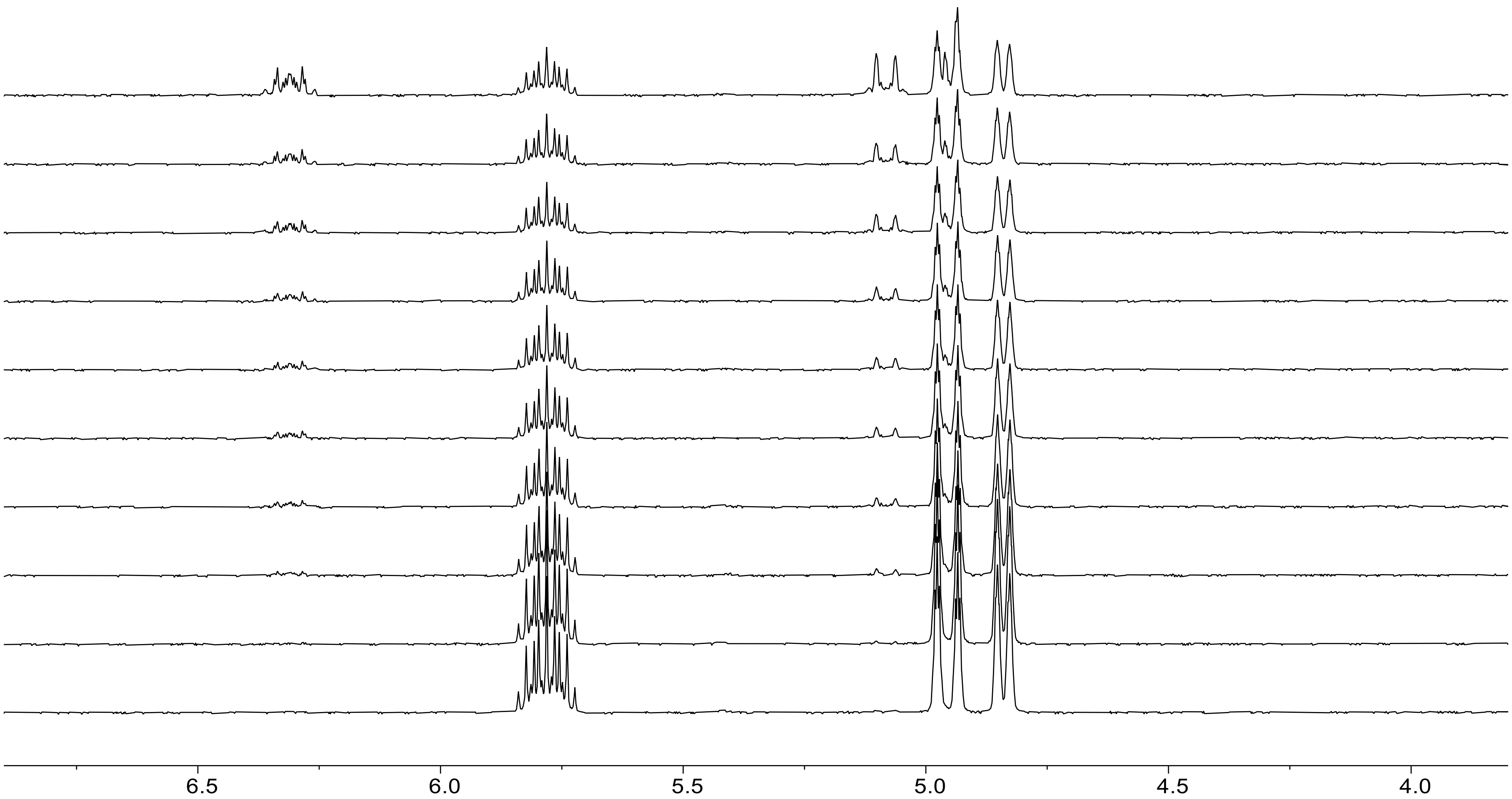

Figure S49. *Stack plot of gas phase NMR spectra recorded at 298 K demonstrating the slow growth over 30 hours of resonances that are attributed to 1,3-butadiene, δ 6.31, 5.08 and 4.96 (obscured).*

Solid/Gas Alkyne Cyclotrimerisation

General Procedure

[Rh(^{t}Bu$_2$PCH$_2$CH$_2$P^{t}Bu$_2$)(propene)][BAr$^F{}_4$] **1** (*ca.* 6 µmol) in a valved NMR tube was hydrogenated (2 bar absolute) for 90 minutes before evacuation and re-pressurisation with propene (2 bar absolute). The sample was stored at room temperature for 120 minutes before the tube was re-evacuated and back-filled with 1-butyne (1 bar absolute). The tube was stored at ambient temperature for 24 hours, 72 hours, 7 days or 28 days before it was evacuated and 1,2-$C_6H_4F_2$ (*ca.* 0.6 mL) was condensed into the tube under vacuum and the contents assayed by ^{31}P NMR spectroscopy. The sample after 72 hours was also analyzed by direct injection of the solution in 1,2-$C_6H_4F_2$ *via* a syringe pump, inside a purpose built dinitrogen glovebox,[14] into a Bruker HCT II Ultra Quadrupole Ion Trap ESI mass spectrometer operating in positive ion mode.

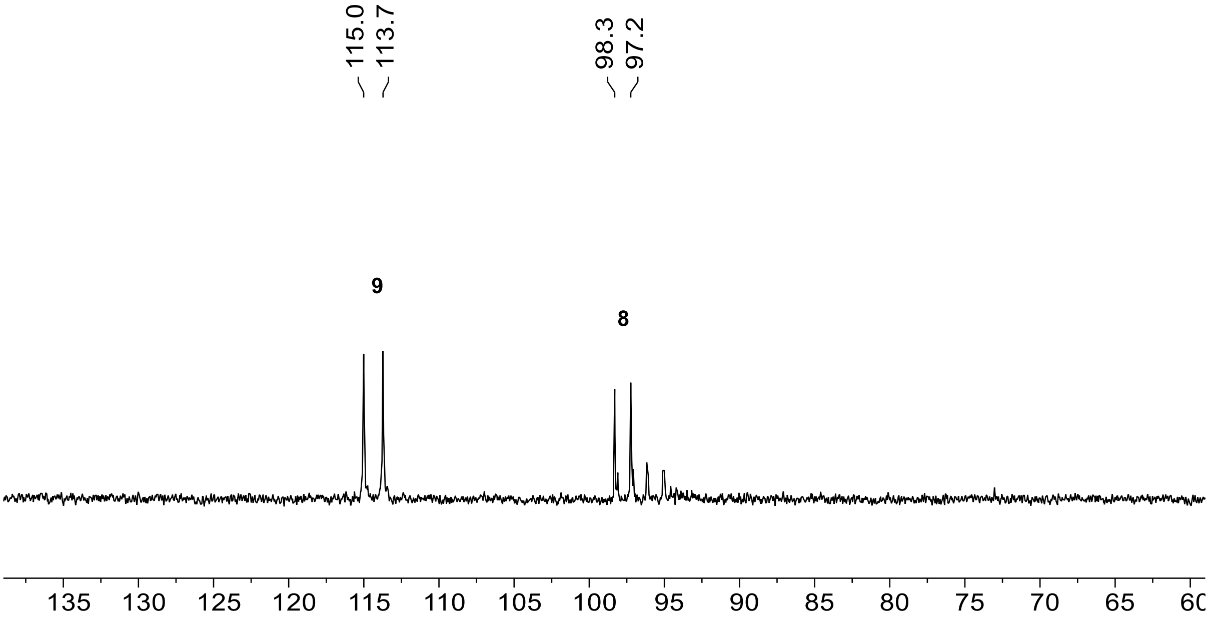

Figure S50. *^{31}P{^{1}H} NMR spectrum recorded after dissolution of the products of the solid/gas reaction between [Rh(^{t}Bu$_2$PCH$_2$CH$_2$P^{t}Bu$_2$)(propene)][BAr$^F{}_4$]* ***1*** *and 1-butyne after 24 hours (161.99 MHz, 1,2-$C_6H_4F_2$, 298 K).*

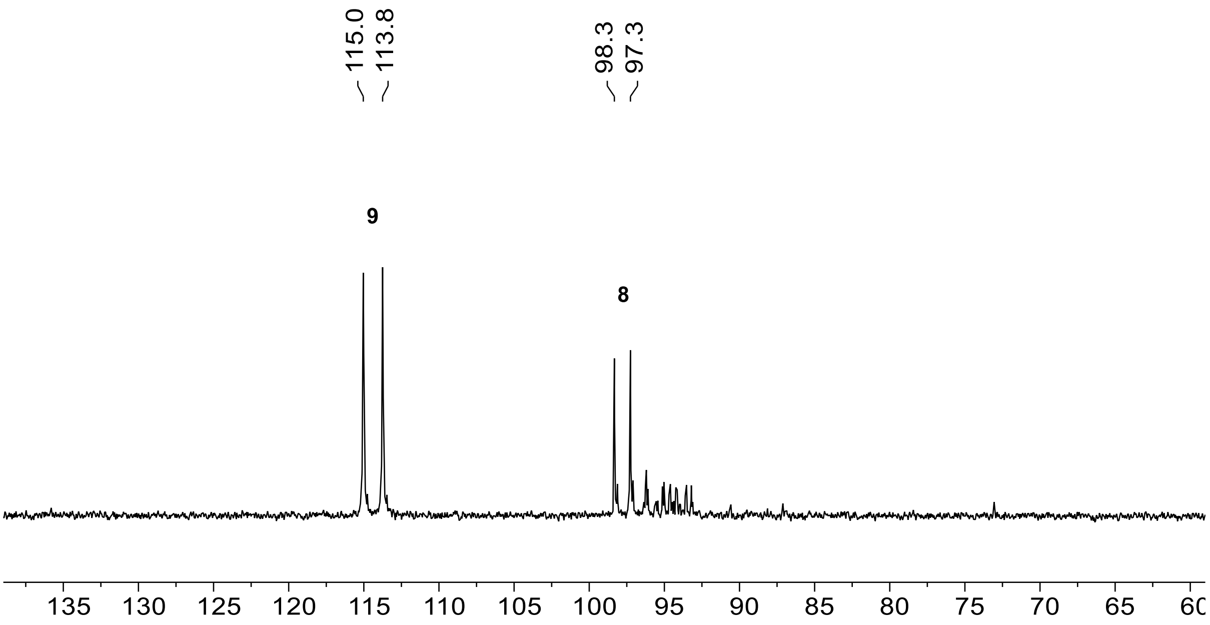

Figure S51. *$^{31}P\{^1H\}$ NMR spectrum recorded after dissolution of the products of the solid/gas reaction between [Rh($^tBu_2PCH_2CH_2P^tBu_2$)(propene)][BAr^F_4]* ***1*** *and 1-butyne after 72 hours (161.99 MHz, 1,2-$C_6H_4F_2$, 298 K).*

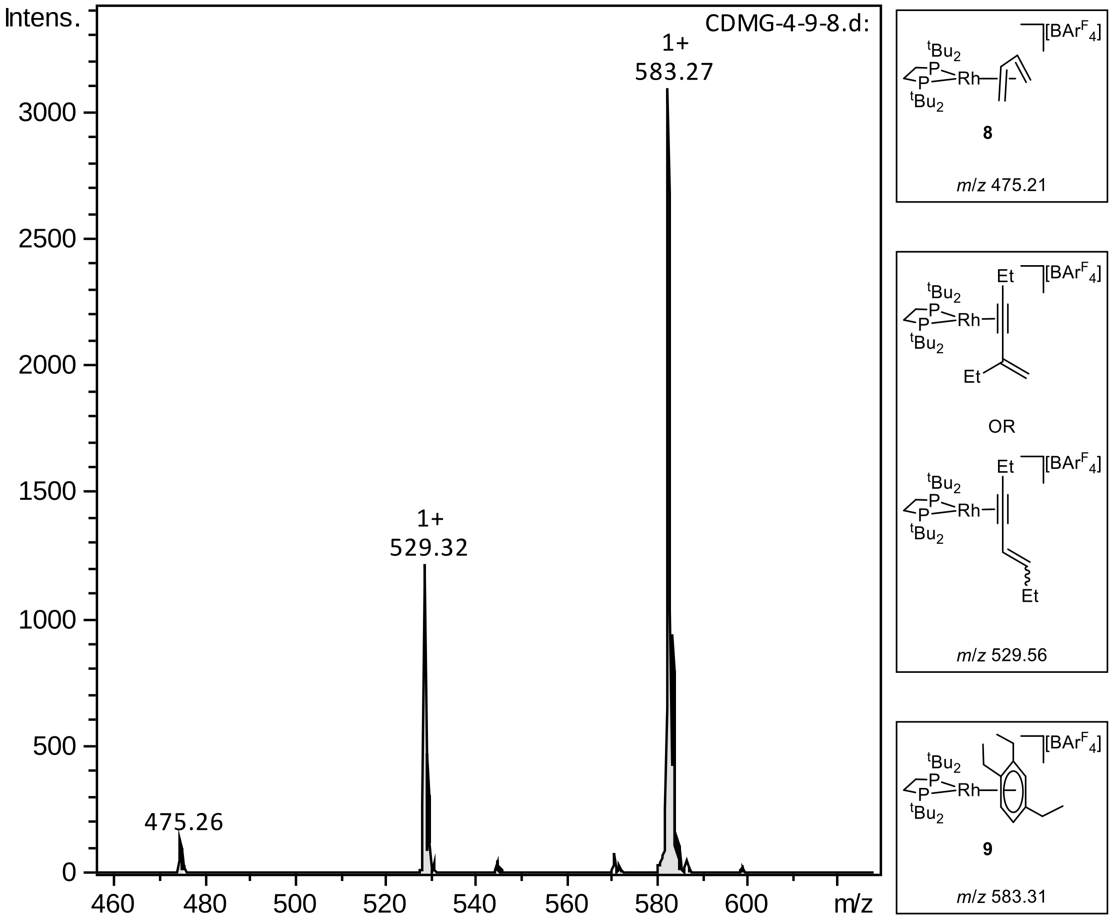

Figure S52. *ESI MS recorded after dissolution in 1,2-$C_6H_4F_2$ of the products of the solid/gas reaction between [Rh($^tBu_2PCH_2CH_2P^tBu_2$)(propene)][BAr^F_4]* ***1*** *and 1-butyne after 72 hours. Calculated* m/z for proposed structures are depicted at right.

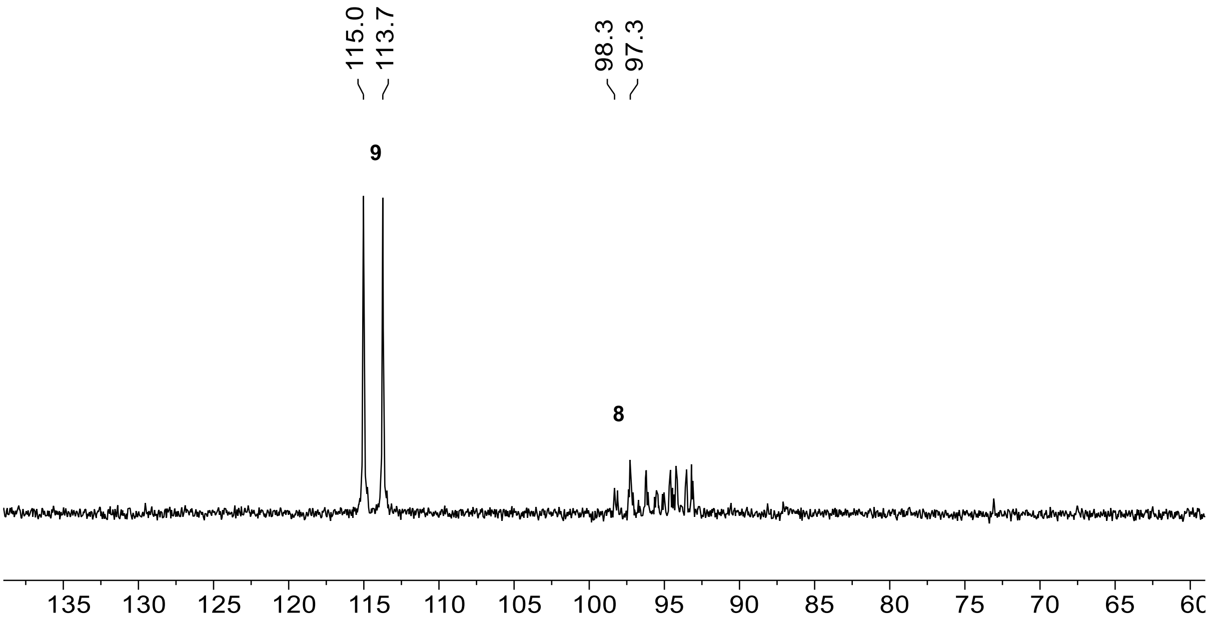

Figure S53. *$^{31}P\{^{1}H\}$ NMR spectrum recorded after dissolution of the products of the solid/gas reaction between [Rh($^{t}Bu_2PCH_2CH_2P^{t}Bu_2$)(propene)][BAr$^{F}_4$]* ***1*** *and 1-butyne after 7 days (161.99 MHz, 1,2-$C_6H_4F_2$, 298 K).*

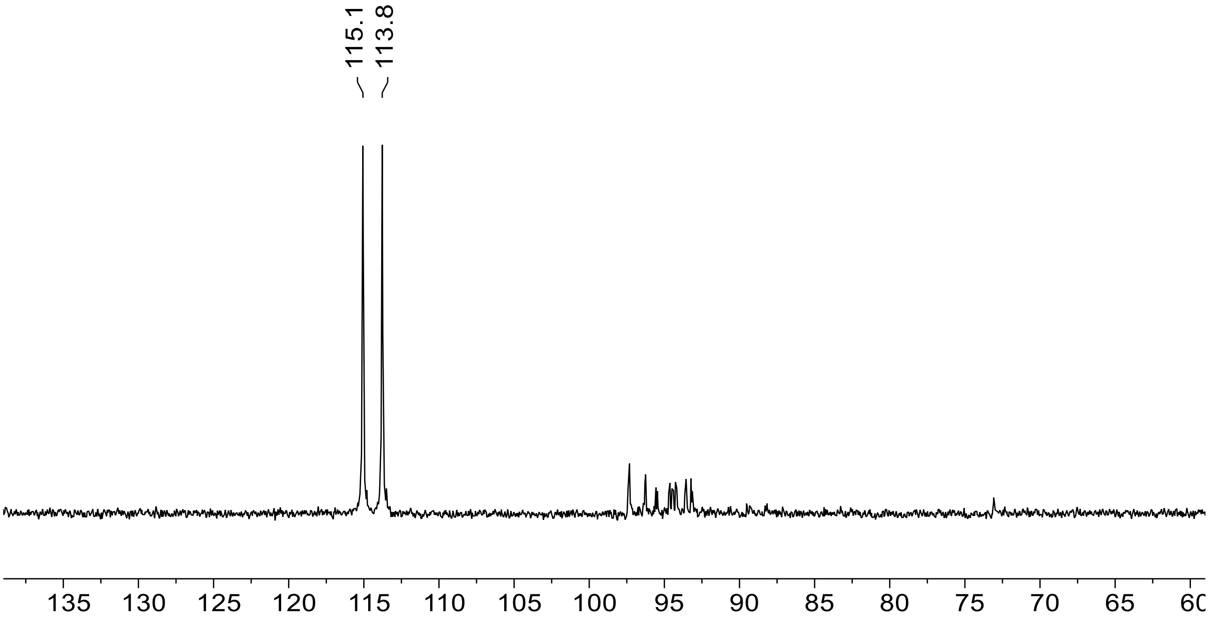

Figure S54. *$^{31}P\{^{1}H\}$ NMR spectrum recorded after dissolution of the products of the solid/gas reaction between [Rh($^{t}Bu_2PCH_2CH_2P^{t}Bu_2$)(propene)][BAr$^{F}_4$]* ***1*** *and 1-butyne after 28 days (161.99 MHz, 1,2-$C_6H_4F_2$, 298 K).*

Gas Chromatographic Analysis of C_8 Coupled Fragments

[Rh(^{t}Bu$_2$PCH$_2$CH$_2$P^{t}Bu$_2$)(propene)][BAr$^F{}_4$] (36.0 mg, 26.1 µmol) was placed under an atmosphere of 1-butyne (1 bar absolute) and stored at room temperature for 30 minutes. The tube was then evacuated and CD_3CN (*ca*. 0.6 mL) condensed in under vacuum, the tube sealed under static vacuum and the tube and contents stood for 60 minutes at ambient temperature under static vacuum Volatile materials were then condensed on the high vacuum line into a separate tube and analysed by ^{1}H NMR and Gas Chromatography with the following method:

Inlet:250 C, Splitless; Oven: Start 50 C; Ramp 1 5 C/min to 280 C 5 min hold; Total run time 51 min

Column HP1 (DB1) 0.25 um FT l:30m Col ID 0.32mm

Detector FID: Temp 330 °C

Decane Retention Time (Boiling Point 174 °C) = 10.64 min

Dodecane Retention Time (Boiling Point 216 °C) = 22.673 min

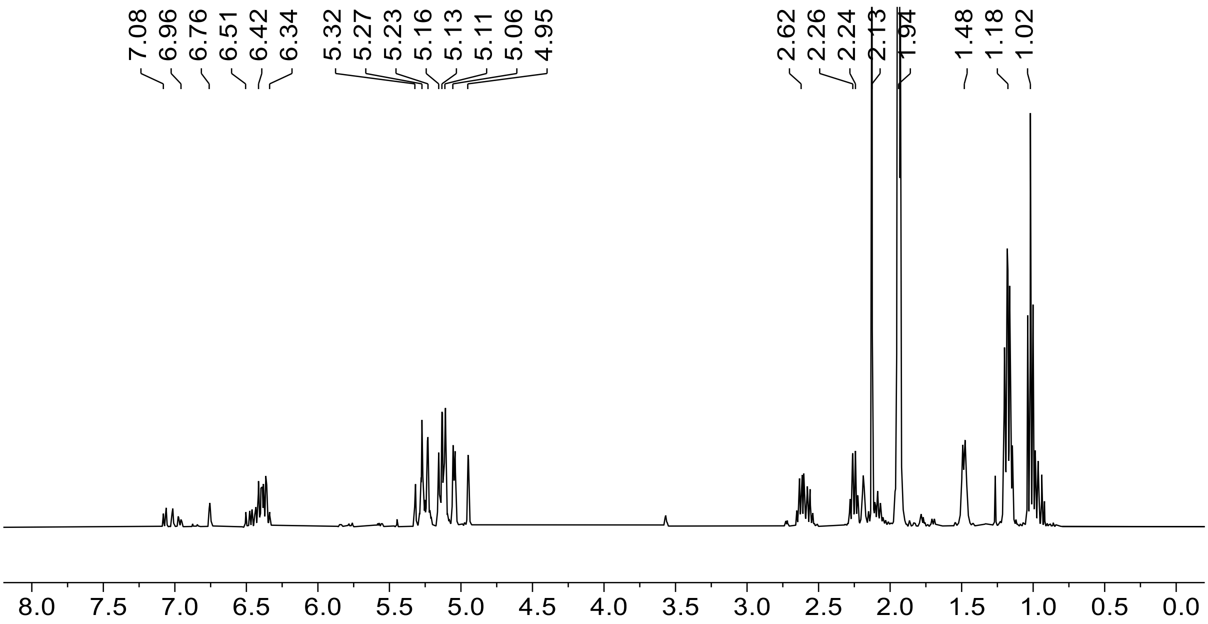

Figure S55. *^{1}H NMR spectrum of condensable materials released on treatment with acetonitrile (400.12 MHz, CD_3CN, 298 K).*

Chromatogram and Results

Injection Details

Injection Name:	**20221027_MG_CDMG4-39**	*Run Time (min):*	**67.50**
Vial Number:	**9**	*Injection Volume:*	**1.00**
Injection Type:	**Unknown**	*Channel:*	**FrontDetector**
Calibration Level:		*Wavelength:*	**n.a.**
Instrument Method:	**20220915_MG_C8toC12_M2**	*Bandwidth:*	**n.a.**
Processing Method:	**Basic Quantitative**	*Dilution Factor:*	**1.0000**
Injection Date/Time:	**27/Oct/22 19:10**	*Sample Weight:*	**1.0000**

Chromatogram

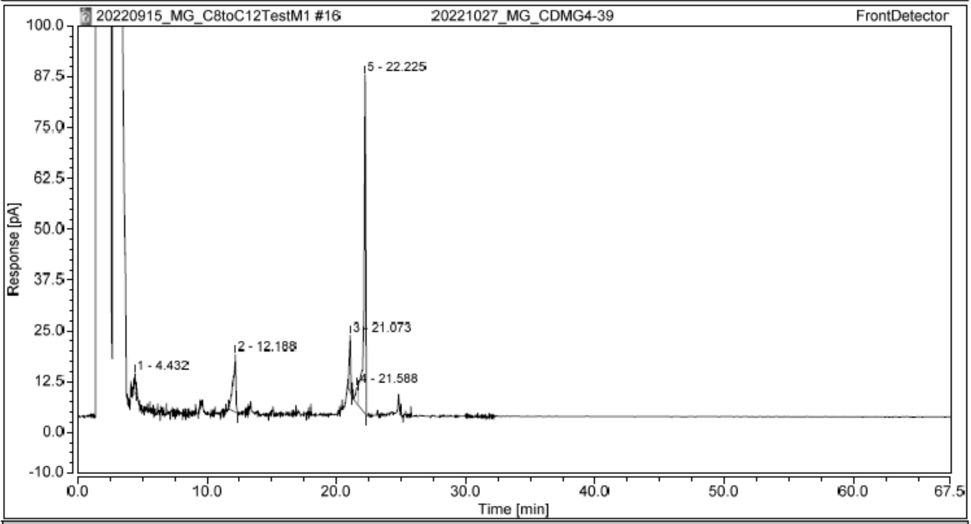

Integration Results

No.	Peak Name	Retention Time min	Area pA*min	Height pA	Relative Area %	Relative Height %	Amount n.a.
1		4.432	0.613	4.800	3.30	3.99	n.a.
2		12.188	3.612	14.111	19.46	11.72	n.a.
3		21.073	1.745	13.998	9.40	11.62	n.a.
4		21.588	0.740	4.085	3.99	3.39	n.a.
5		22.225	11.854	83.443	63.85	69.28	n.a.
**Total:**			**18.565**	**120.437**	**100.00**	**100.00**	

Gas Chromatographic Analysis of C_8 Coupled and Hydrogenated Fragments

[Rh(^{t}$Bu_2PCH_2CH_2P^tBu_2$)(propene)][BAr^F_4] (37.2 mg, 27.0 μmol) was placed under an atmosphere of 1-butyne (1 bar absolute) and stored at room temperature for 30 minutes. The tube was then evacuated and refilled with H_2 (4 bar absolute) and stored at room temperature overnight. The tube was then cooled in liquid nitrogen, evacuated and CD_3CN (*ca*. 0.6 mL) condensed in under vacuum, the tube sealed under static vacuum and the tube and contents stood for 60 minutes at ambient temperature under static vacuum Volatile materials were then condensed on the high vacuum line into a separate tube and analysed by ^1H NMR and Gas Chromatography with the following method:

Inlet:250 C, Splitless; Oven: Start 50 C; Ramp 1 5 C/min to 280 C 5 min hold; Total run time 51 min

Column HP1 (DB1) 0.25 um FT l:30m Col ID 0.32mm

Detector FID: Temp 330 °C

Decane Retention Time (Boiling Point 174 °C) = 10.64 min

Dodecane Retention Time (Boiling Point 216 °C) = 22.673 min

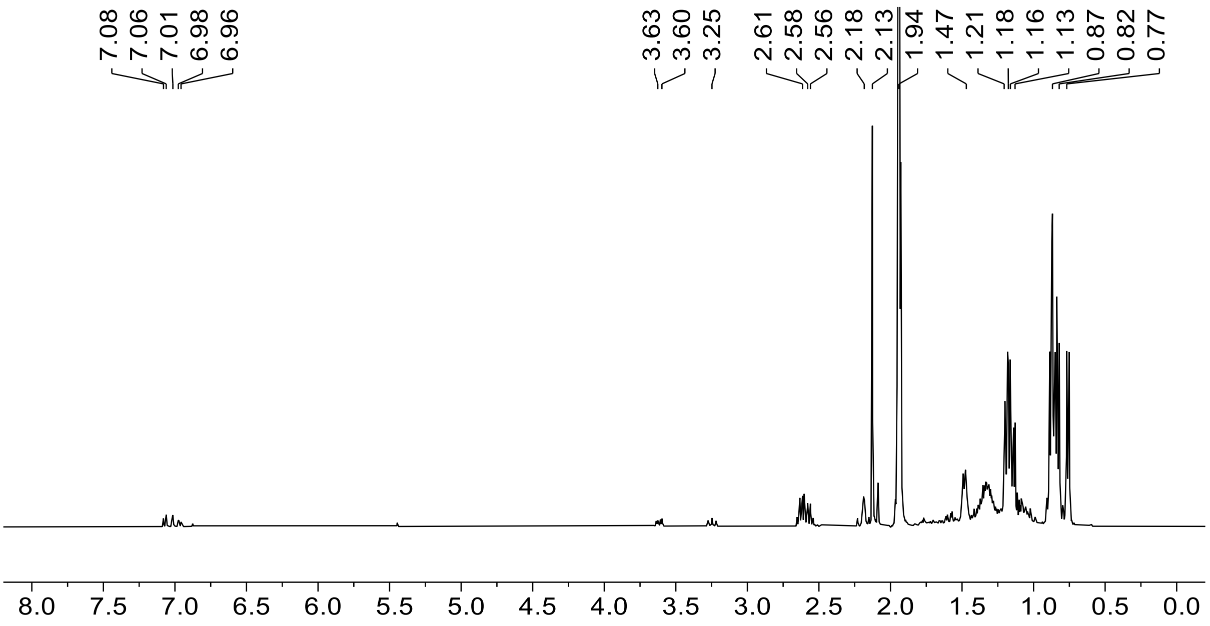

Figure S56. *^{1}H NMR spectrum of condensable materials released on treatment with acetonitrile after solid/gas hydrogenation (400.12 MHz, CD_3CN, 298 K).*

Chromatogram and Results

Injection Details

Injection Name:	**20221027_MG_CDMG4-40**	*Run Time (min):*	**67.50**
Vial Number:	**10**	*Injection Volume:*	**1.00**
Injection Type:	**Unknown**	*Channel:*	**FrontDetector**
Calibration Level:		*Wavelength:*	**n.a.**
Instrument Method:	**20220915_MG_C8toC12_M2**	*Bandwidth:*	**n.a.**
Processing Method:	**Basic Quantitative**	*Dilution Factor:*	**1.0000**
Injection Date/Time:	**27/Oct/22 20:34**	*Sample Weight:*	**1.0000**

Chromatogram

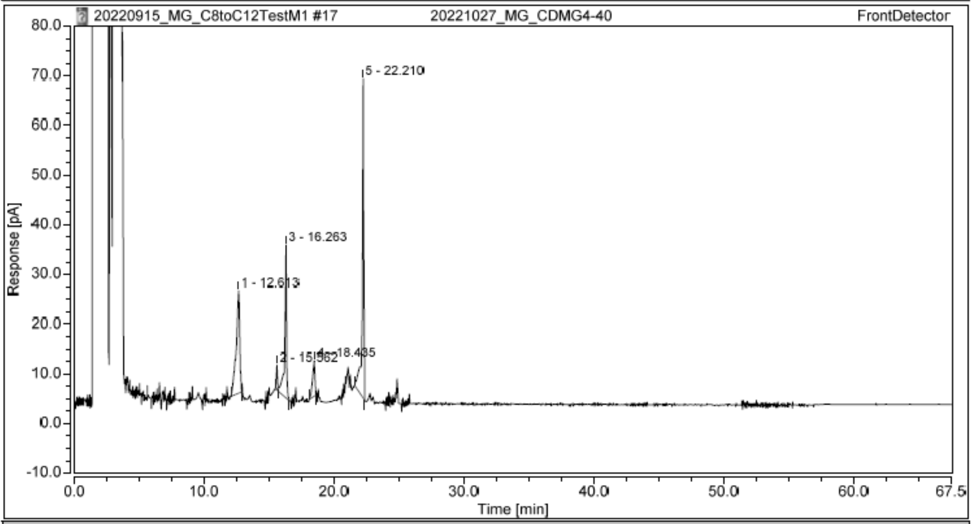

Integration Results

No.	Peak Name	Retention Time min	Area pA*min	Height pA	Relative Area %	Relative Height %	Amount n.a.
1		12.613	6.431	20.571	32.10	16.13	n.a.
2		15.562	0.333	4.696	1.66	3.68	n.a.
3		16.263	4.232	30.794	21.12	24.15	n.a.
4		18.435	1.460	7.118	7.29	5.58	n.a.
5		22.210	7.579	64.349	37.83	50.46	n.a.
**Total:**			**20.036**	**127.528**	**100.00**	**100.00**	

Solution Alkyne Cyclotrimerisation

Experimental

[Rh(^{t}Bu$_2$PCH$_2$CH$_2$P^{t}Bu$_2$)(norbornadiene)][BAr$^F{}_4$] **1** (10 mg, 7.3 μmol) in a valved NMR tube was hydrogenated (2 bar absolute) for 90 minutes before evacuation and re-pressurisation with propene (2 bar absolute). The sample was stored at room temperature for 120 minutes before the tube was re-evacuated 1,2-$C_6H_4F_2$ (*ca.* 0.6 mL) was condensed into the tube under vacuum and then back-filled with 1-butyne (1 bar absolute) and the contents monitored by ^{31}P NMR spectroscopy over 24 hours.

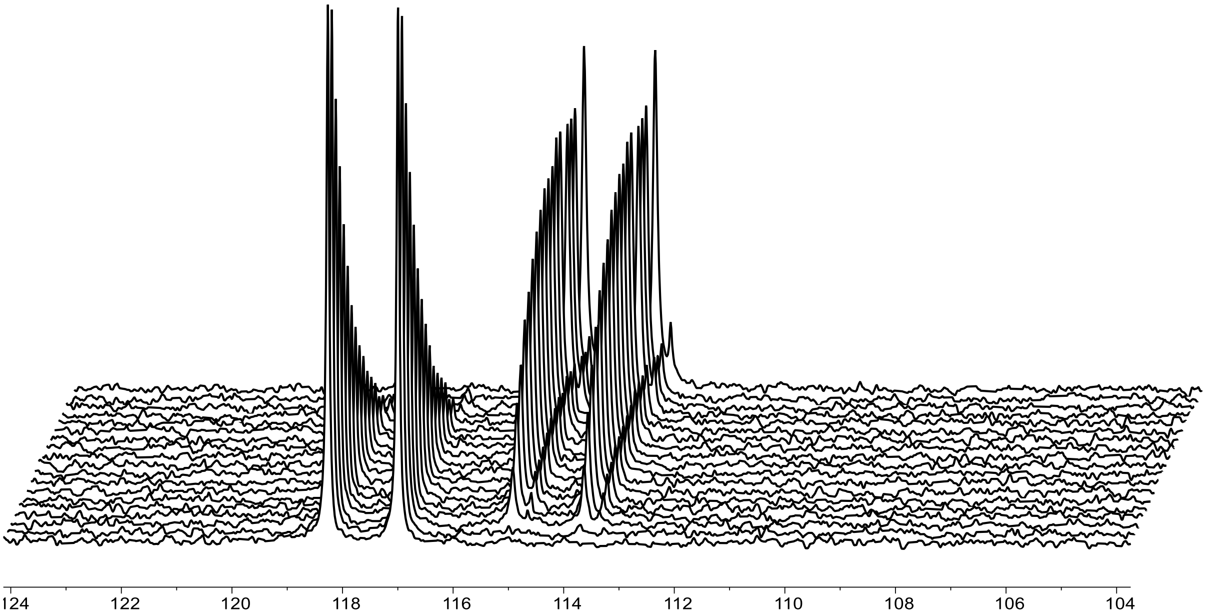

Figure S57. *Stack plot of ^{31}P{^{1}H} NMR spectra demonstrating the cyclotrimerization of 1-butyne by [Rh(^{t}Bu$_2$PCH$_2$CH$_2$P^{t}Bu$_2$)(1,2-C$_6$H$_4$F$_2$)][BAr$^F{}_4$] (δ 117.6) over 24 hours at 298 K to form [Rh(^{t}Bu$_2$PCH$_2$CH$_2$P^{t}Bu$_2$)(1,2,4-Et$_3$C$_6$H$_3$))][BAr$^F{}_4$] (δ 114.3) and [Rh(^{t}Bu$_2$PCH$_2$CH$_2$P^{t}Bu$_2$)(1,3,5-Et$_3$C$_6$H$_3$))][BAr$^F{}_4$] (δ 114.0).*

Gas Phase Catalysis

General Procedure

A 5 mm thin wall NMR tube fitted with a high vacuum PTFE (J. Young) valve containing the appropriate quantity of sieved catalyst (1.8-2.2 mg, 1.4-1.6 μmol generally; 0.3-0.5 mg for 1-butene hydrogenation, packed in a nitrogen glovebox) was evacuated and backfilled with the target substrate gas (1.05-1.08 bar absolute) on a specially constructed stainless-steel vacuum/*para*-hydrogen/substrate gas NMR tube triple manifold as has been described previously.[10,11] Tubes for experiments involving gas mixtures (1-butene and propene, 1.5 bar absolute) were filled by condensation of the individual gas (0.75 bar absolute) into the tube with the aid of a mercury manometer on a glass high vacuum line. The tube was sealed at this pressure and the headspace above the sealed tube evacuated and re-filled (4 bar absolute) with *para*-hydrogen five times on the stainless steel manifold. The manifold was isolated from the *para*-hydrogen source after a final refill to a static pressure (4 bar absolute). The valve was then opened at the NMR tube to equilibrate substrate gas and *para*-hydrogen pressures (final system pressure ~3.5 bar absolute) in the NMR tube, which was then rapidly sealed and vigorously shaken during transit to the NMR spectrometer (distance ~5 m) before insertion (without use of the spectrometer's lift) and immediate acquisition through means of a pre-loaded experiment ('*autosuspend*' entry before d1 in the Bruker pulse program). The interval between pressurisation and first acquisition is reliably 10 seconds. Subsequent acquisition of data was made through the Bruker AU program 'multizg.' After all the desired data was acquired, the tube was removed from the spectrometer, returned to the gas manifold, evacuated and recharged with the same substrate gas and re-pressurised as above. Data acquisition was completed similarly.

Representative Spectra: Propene

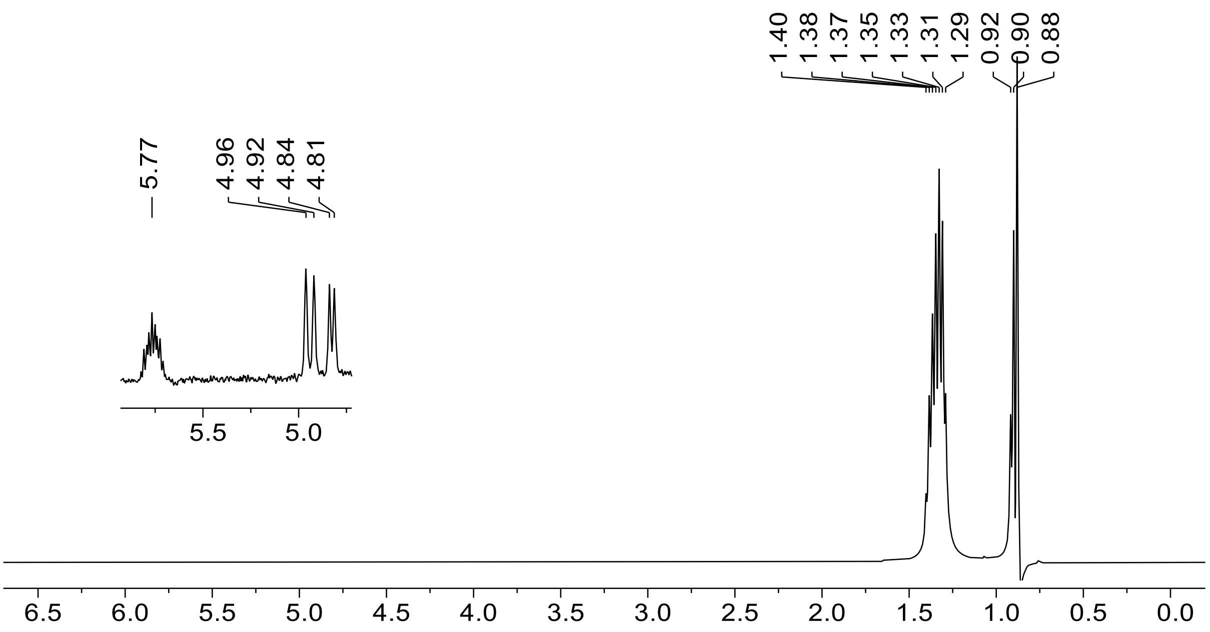

Figure S58. *Gas-phase ^{1}H NMR spectrum of the PHIP hydrogenation of propene to propane using catalyst **1** under mostly ALTADENA conditions. The inset region is magnified approximately 500-fold to demonstrate the propene resonances (298 K).*

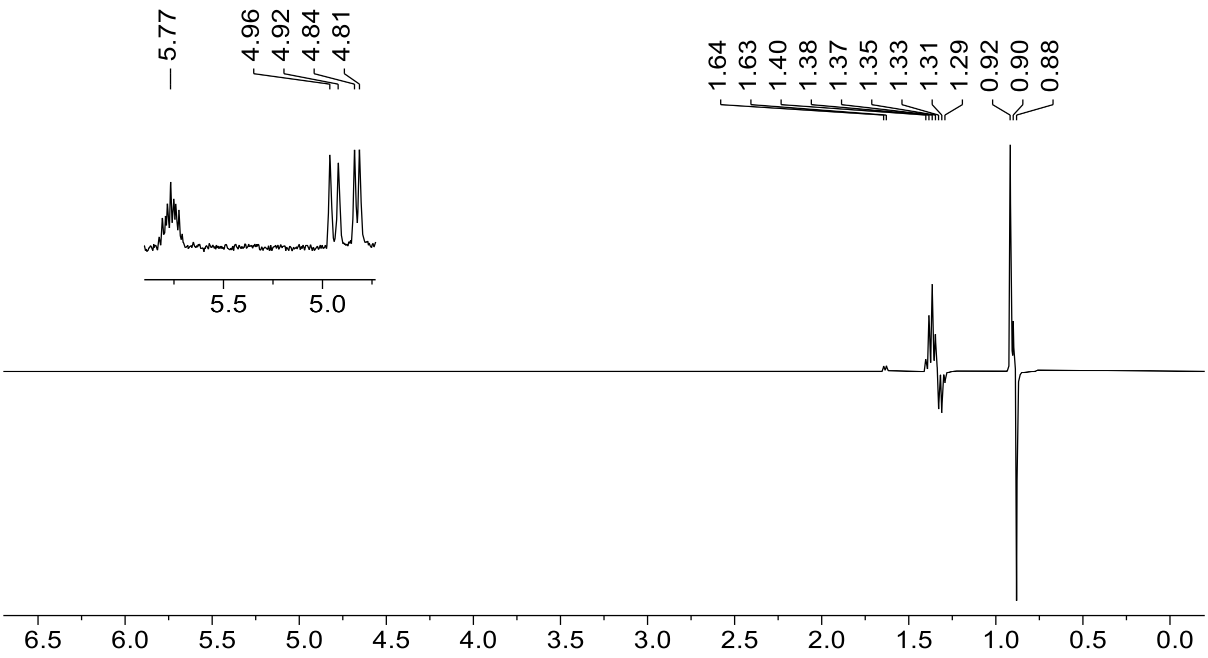

Figure S59. *Gas-phase* ^{1}H *NMR spectrum of the PHIP hydrogenation of propene to propane using catalyst* ***1*** *under mostly PASADENA conditions (approximately 30 seconds after insertion into the spectrometer). The inset region is magnified approximately 100-fold to demonstrate the propene resonances (298 K).*

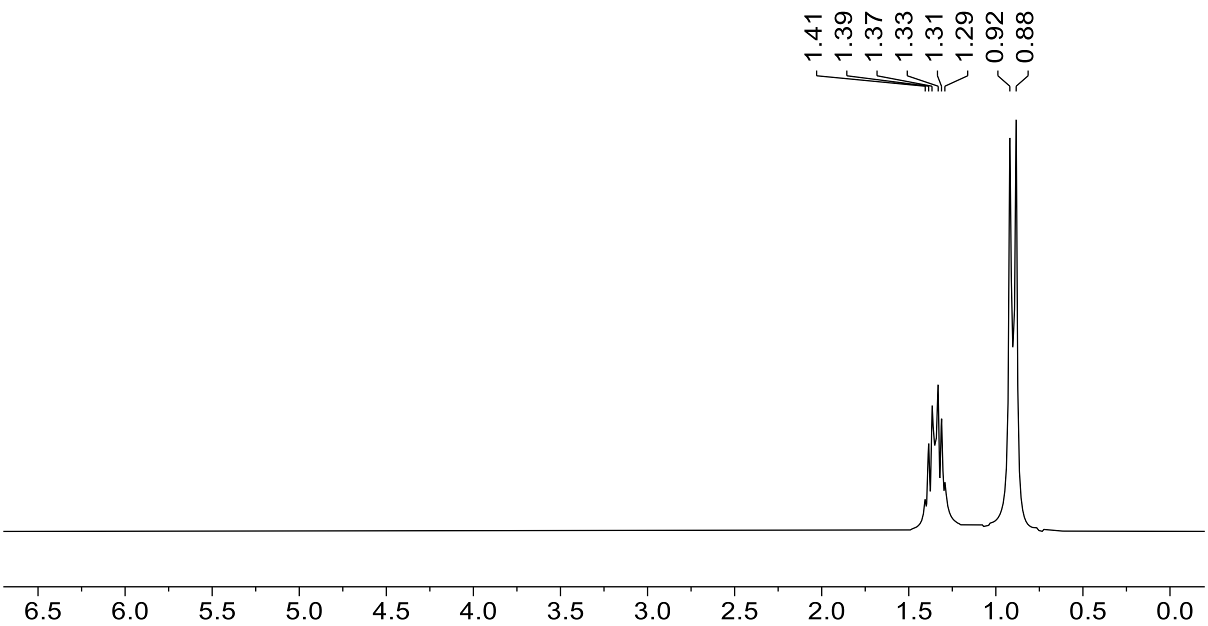

Figure S60. *Gas-phase OPSYdq spectrum of the PHIP hydrogenation of propene to propane using catalyst* ***1****. The spectrum has been magnitude calculated to demonstrate all components in phase (298 K).*

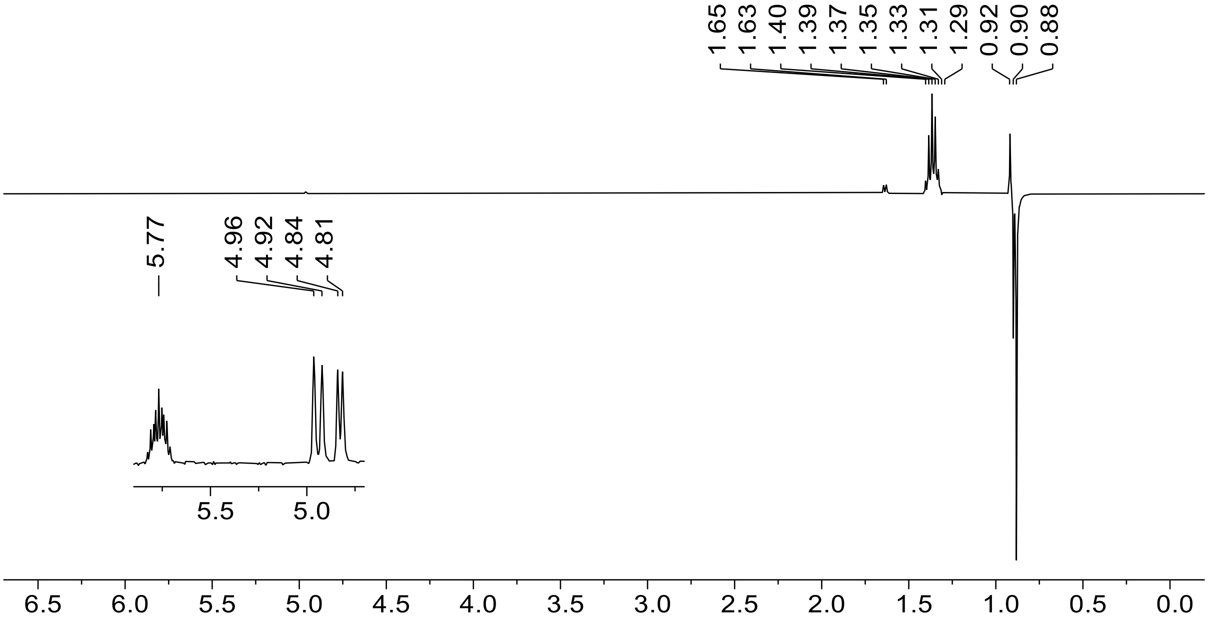

Figure S61. *Gas-phase* ^{1}*H NMR spectrum of the PHIP hydrogenation of propene to propane using catalyst* ***1*** *after two catalysis cycles under mostly ALTADENA conditions. The inset region is magnified approximately 100-fold to demonstrate the propene resonances (298 K).*

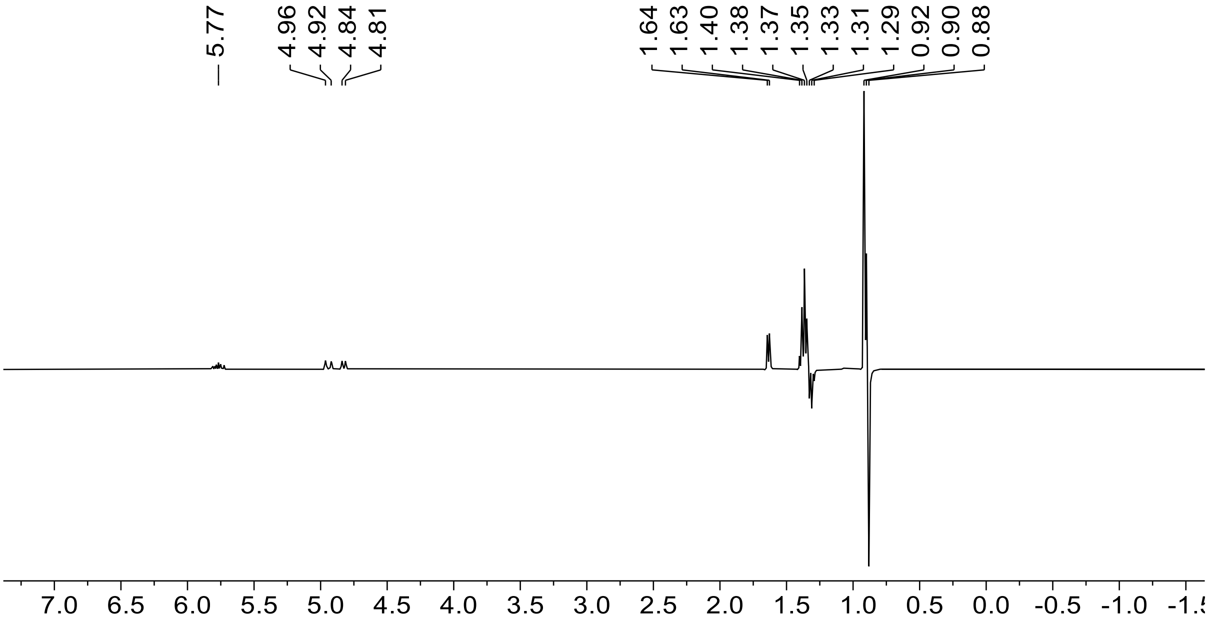

Figure S62. *Gas-phase* ^{1}*H NMR spectrum of the PHIP hydrogenation of propene to propane using catalyst* ***1*** *after two catalysis cycles under mostly PASADENA conditions (approximately 30 seconds after insertion into the spectrometer, 298 K).*

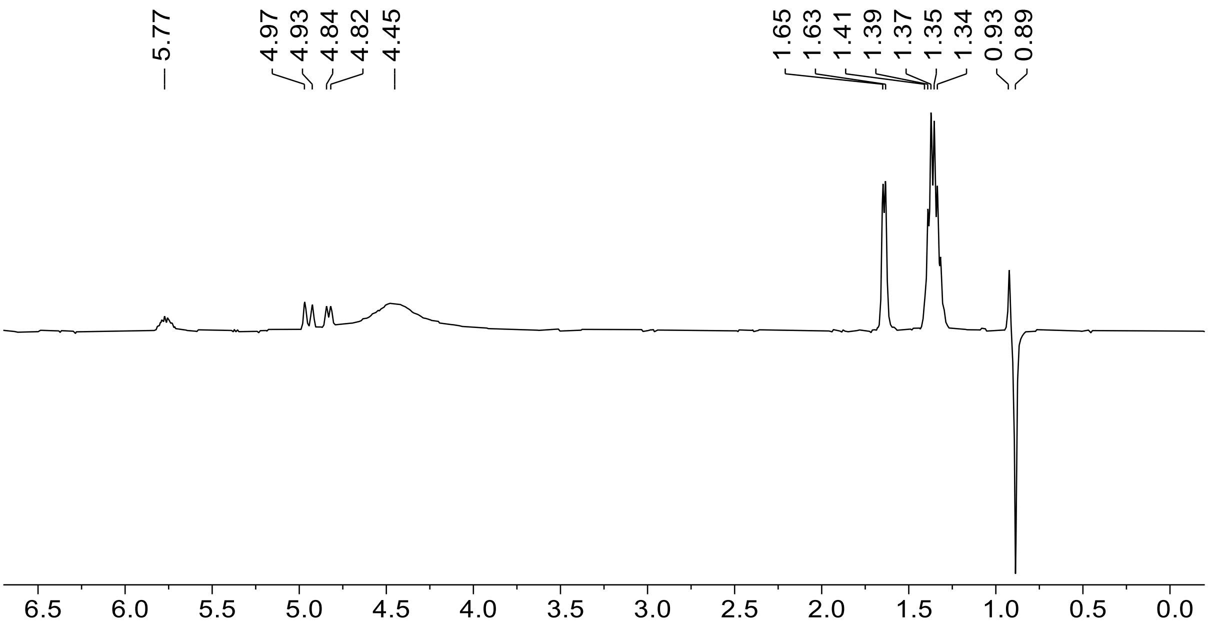

Figure S63. *Gas-phase ^{1}H NMR spectrum of the hydrogenation of propene to propane recorded immediately after insertion into the NMR spectrometer using catalyst **1** and thermally polarised hydrogen (298 K).*

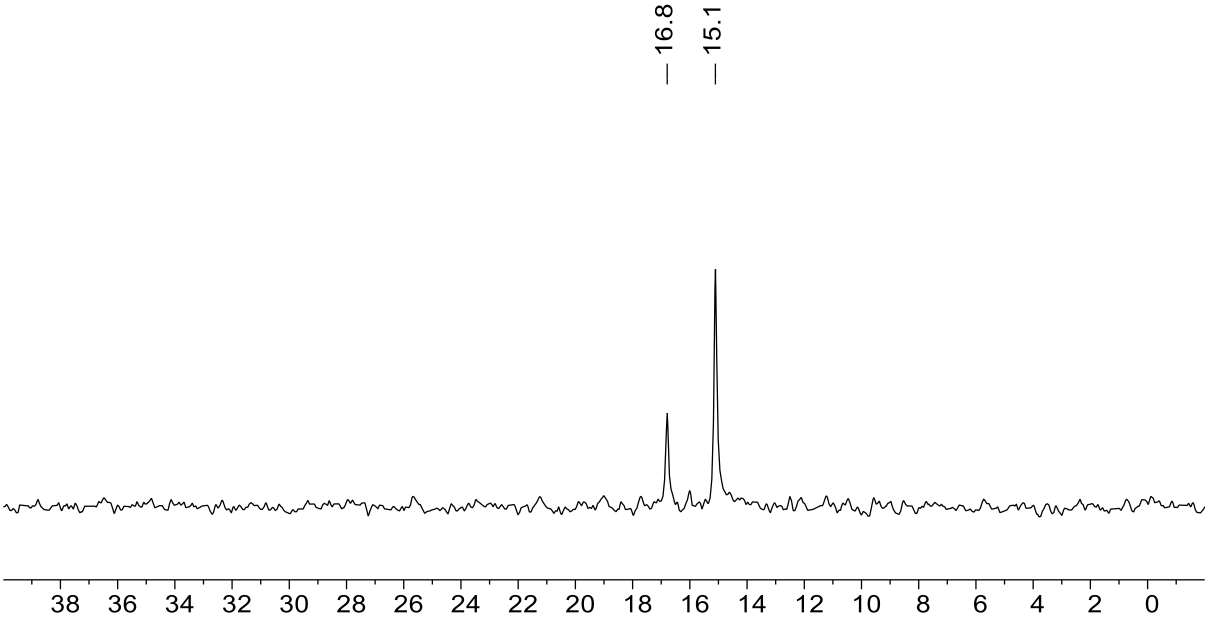

Figure S64. *Gas-phase $^{13}C\{^{1}H\}$ INEPT of the PHIP hydrogenation of propene to propane using catalyst **1** under mostly ALTADENA conditions (298 K).*

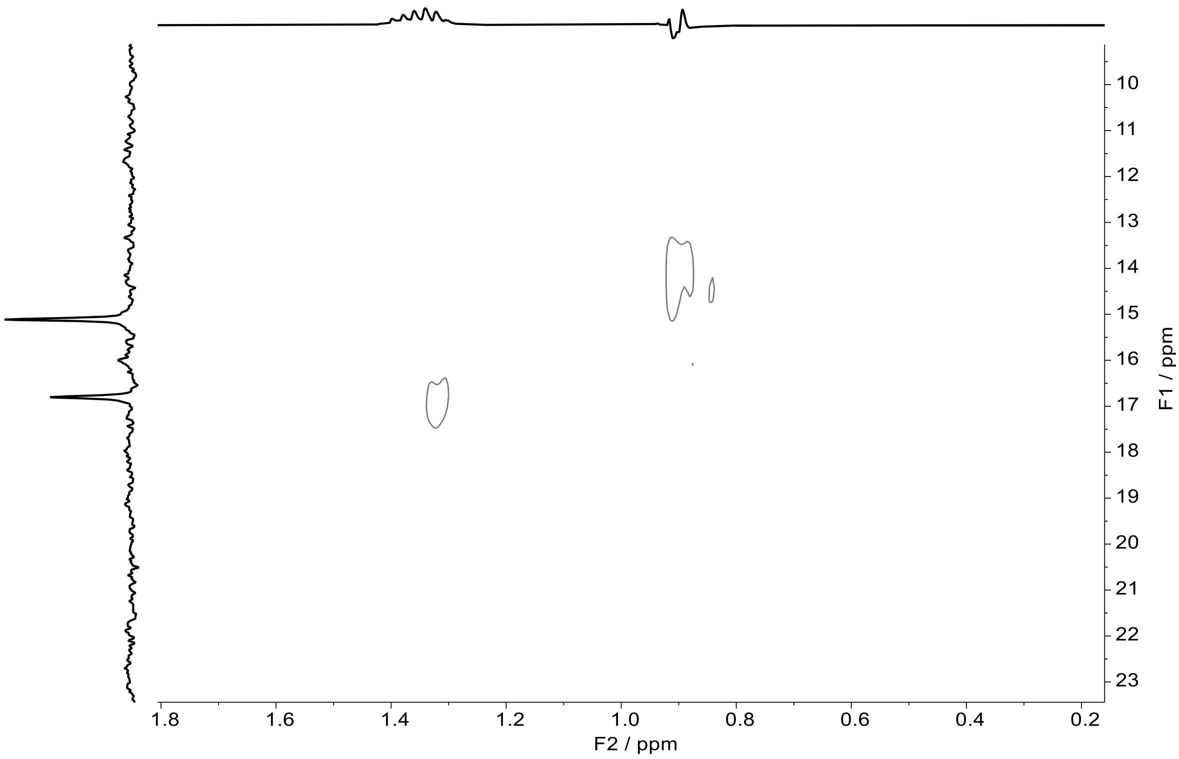

Figure S65. *Gas-phase* ^{13}C*-*^{1}H *HMQC of the PHIP hydrogenation of propene to propane using catalyst* ***1*** *(298 K).*

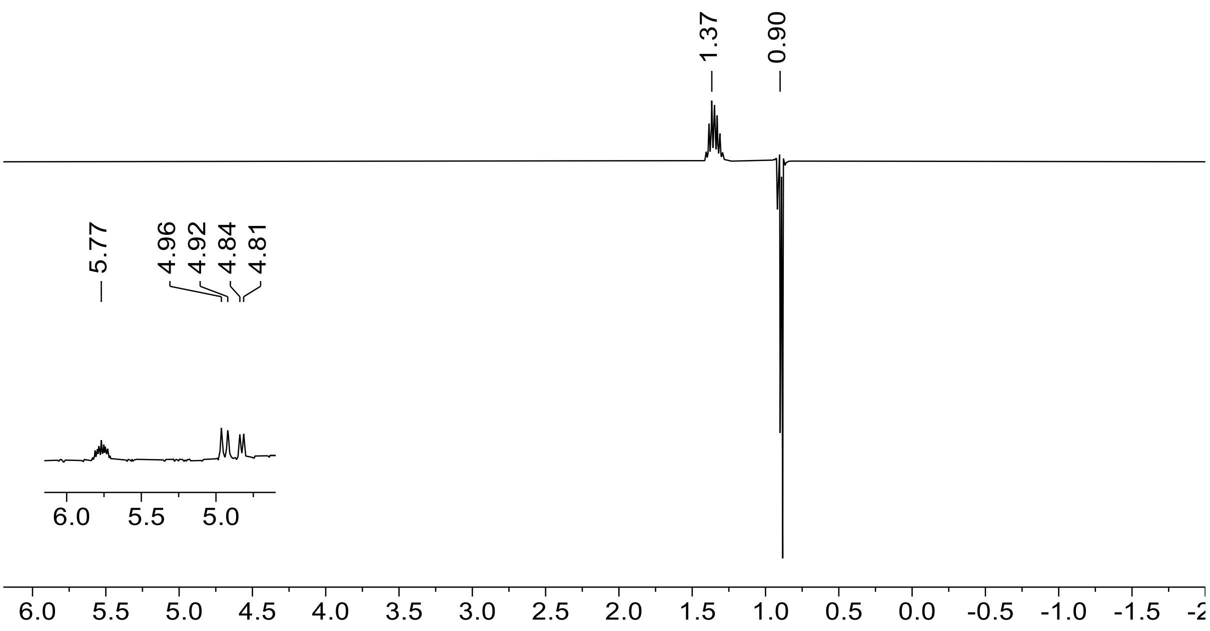

Figure S66. *Gas-phase* ^{1}H *NMR spectrum of the PHIP hydrogenation of propene to propane using catalyst* ***10*** *under mostly ALTADENA conditions. The inset region is magnified approximately 700-fold to demonstrate the propene resonances (298 K).*

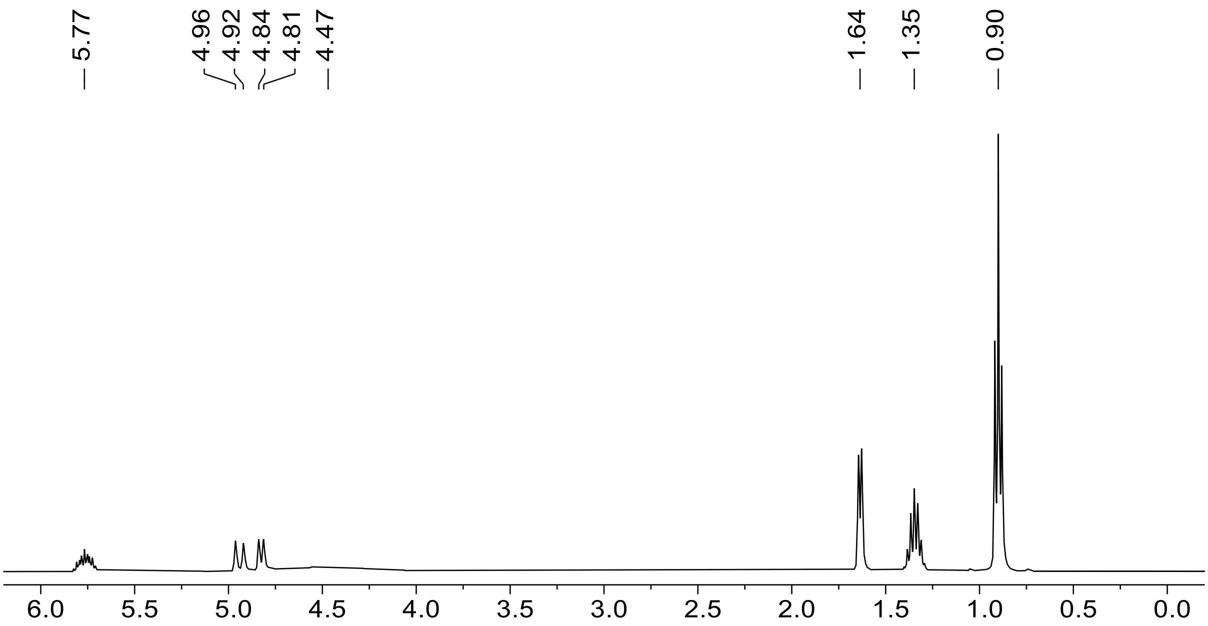

Figure S67. *Gas-phase ^{1}H NMR spectrum of the PHIP hydrogenation of propene to propane using catalyst* ***10*** *under mostly PASADENA conditions (approximately 30 seconds after insertion into the spectrometer, 298 K).*

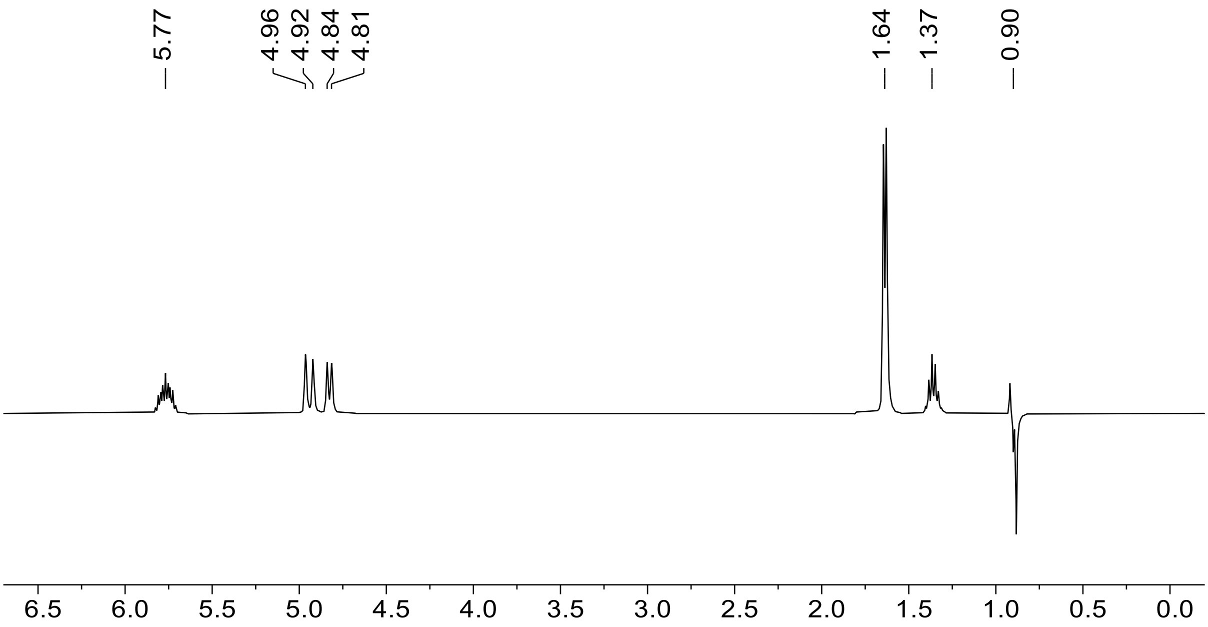

Figure S68. *Gas-phase ^{1}H NMR spectrum of the PHIP hydrogenation of propene to propane using catalyst* ***10*** *after two catalysis cycles under mostly ALTADENA conditions (298 K).*

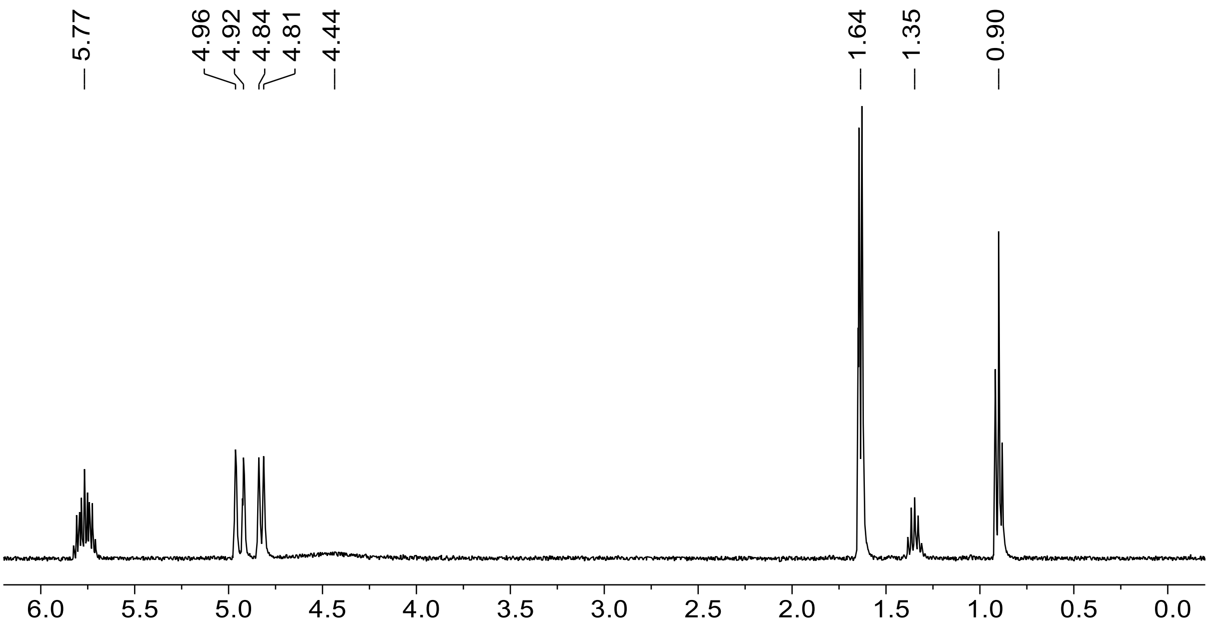

Figure S69. *Gas-phase* [1]*H NMR spectrum of the PHIP hydrogenation of propene to propane using catalyst* ***10*** *after two catalysis cycles under mostly PASADENA conditions (approximately 30 seconds after insertion into the spectrometer, 298 K).*

Representative Spectra: 1-Butene

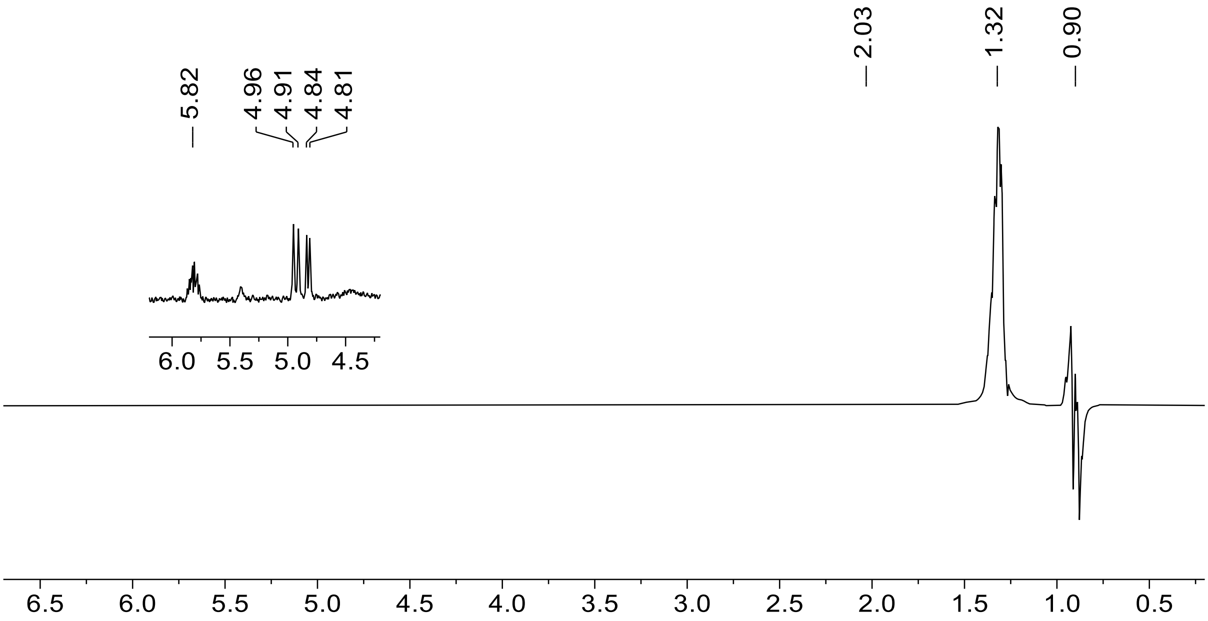

Figure S70. *Gas-phase ^{1}H NMR spectrum of the PHIP hydrogenation of 1-butene to butane using catalyst* ***1*** *under mostly ALTADENA conditions. The inset region is magnified approximately 600-fold to demonstrate the propene resonances (298 K).*

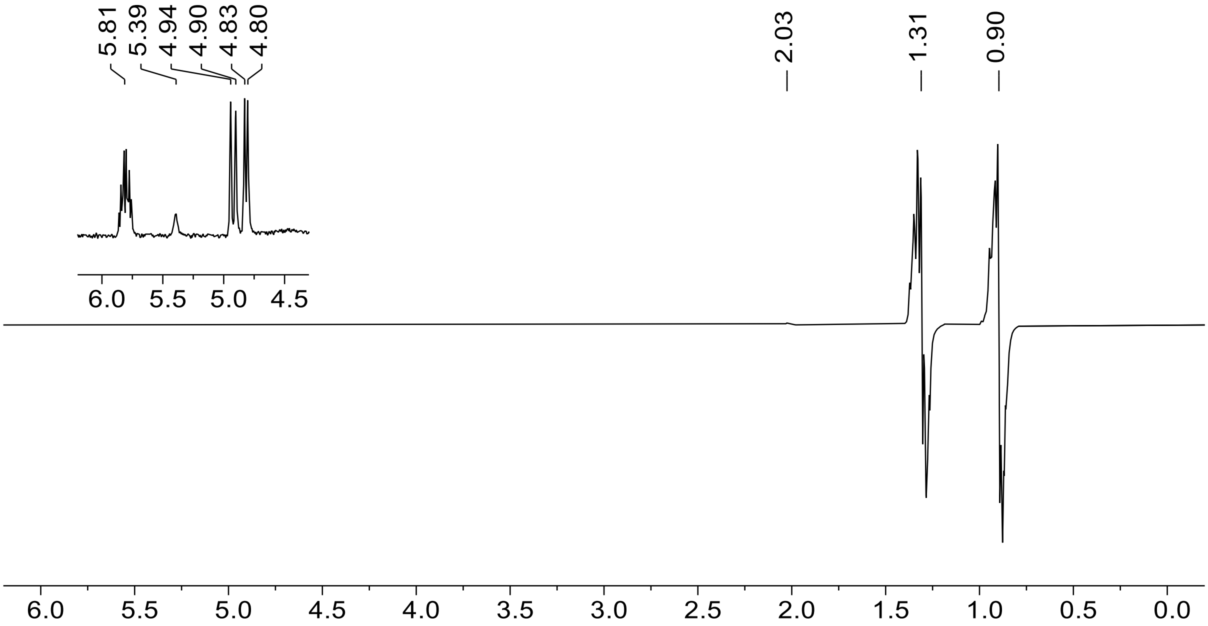

Figure S71. *Gas-phase ^{1}H NMR spectrum of the PHIP hydrogenation of 1-butene to butane using catalyst* ***1*** *under mostly PASADENA conditions (approximately 30 seconds after insertion into the spectrometer). The inset region is magnified approximately 150-fold to demonstrate the 1-butene and 2-butene resonances (298 K).*

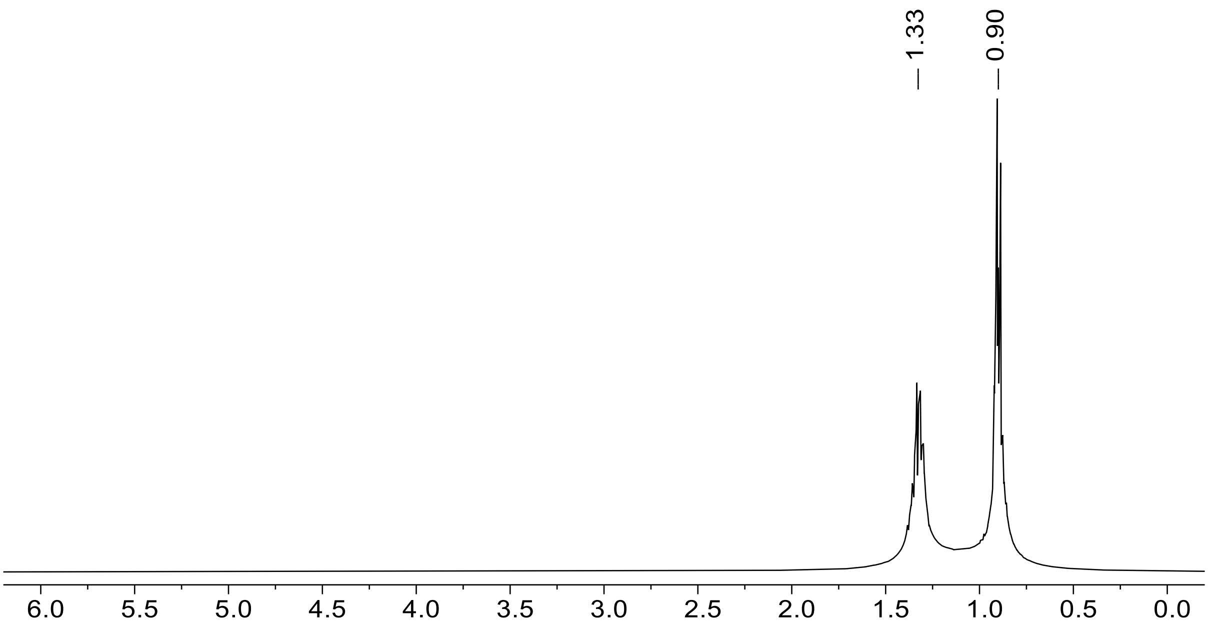

Figure S72. *Gas-phase OPSYdq spectrum of the PHIP hydrogenation of 1-butene to butane using catalyst* ***1****. The spectrum has been magnitude calculated to demonstrate all components in phase (298 K).*

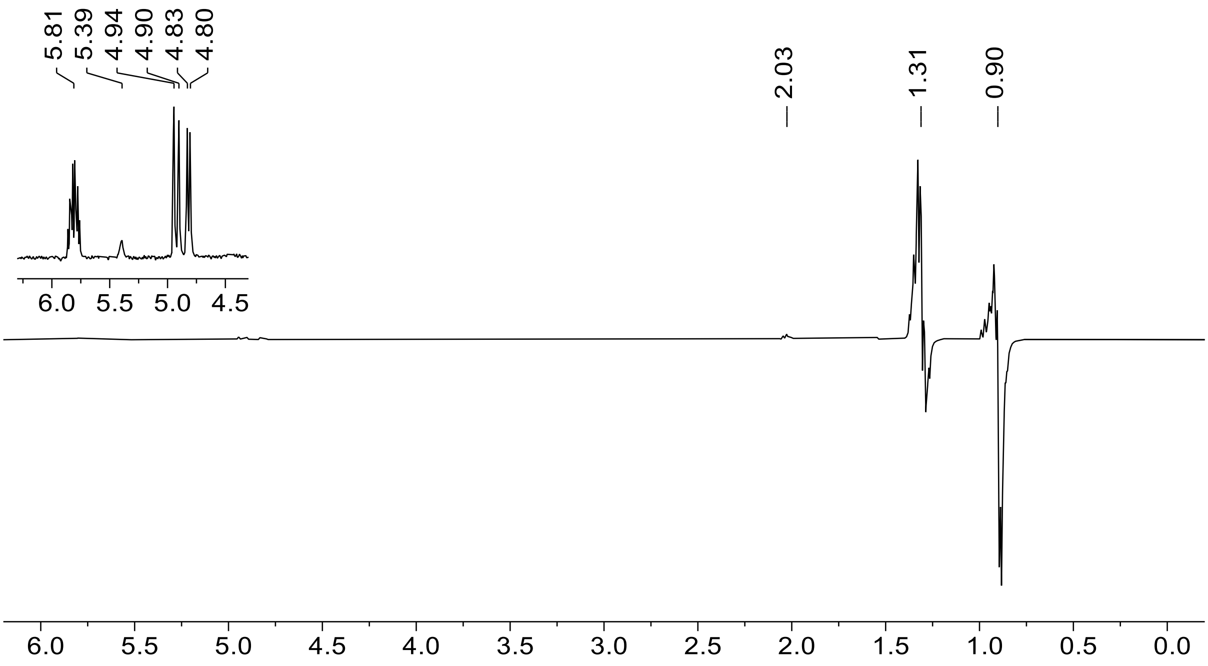

Figure S73. *Gas-phase* ^{1}H *NMR spectrum of the PHIP hydrogenation of 1-butene to butane using catalyst* ***1*** *after two catalysis cycles under mostly ALTADENA conditions. The inset region is magnified approximately 70-fold to demonstrate the 1-butene and 2-butene resonances (298 K).*

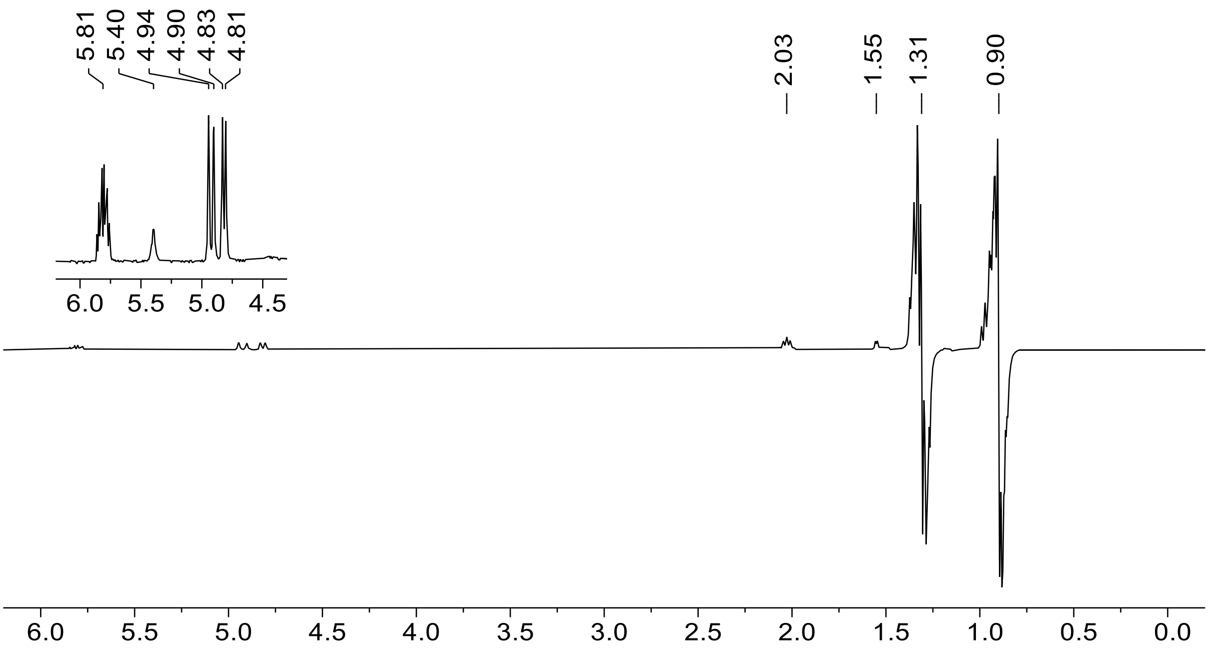

Figure S74. *Gas-phase* ^{1}*H NMR spectrum of the PHIP hydrogenation of 1-butene to butane using catalyst* ***1*** *after two catalysis cycles under mostly PASADENA conditions (approximately 30 seconds after insertion into the spectrometer). The inset region is magnified approximately 25-fold to demonstrate the 1-butene and 2-butene resonances (298 K).*

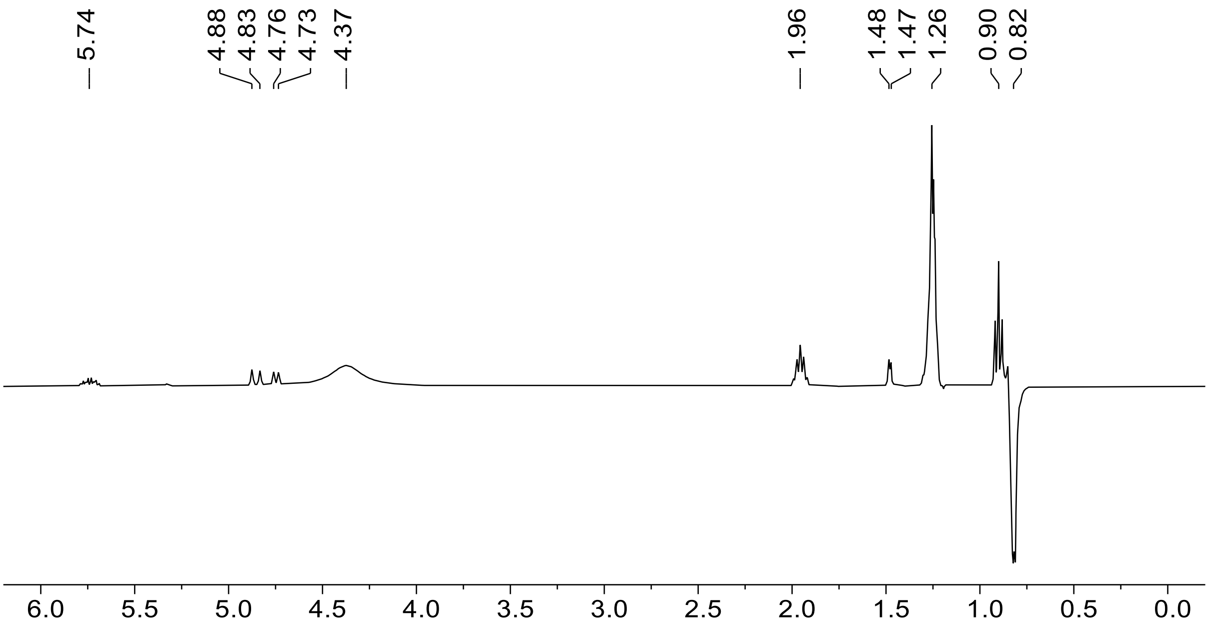

Figure S75. *Gas-phase* ^{1}*H NMR spectrum of the hydrogenation of 1-butene to butane recorded immediately after insertion into the NMR spectrometer using catalyst* ***1*** *and thermally polarised hydrogen (298 K).*

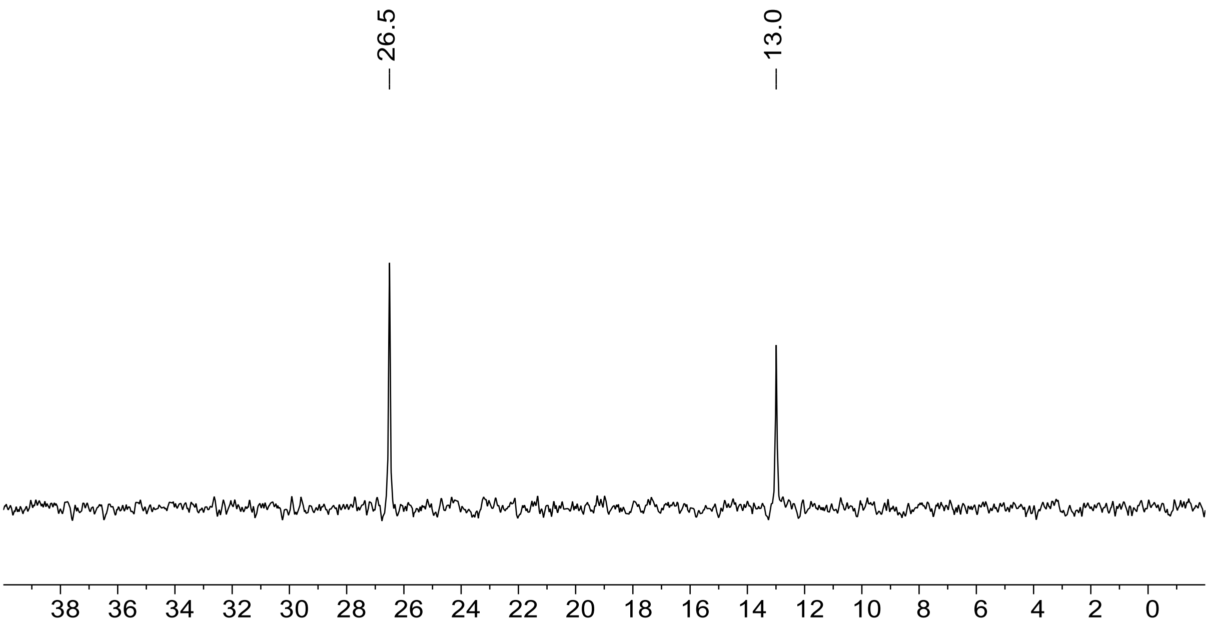

Figure S76. *Gas-phase* $^{13}C\{^1H\}$ *INEPT of the PHIP hydrogenation of 1-butene to butane using catalyst* ***1*** *under mostly ALTADENA conditions (298 K).*

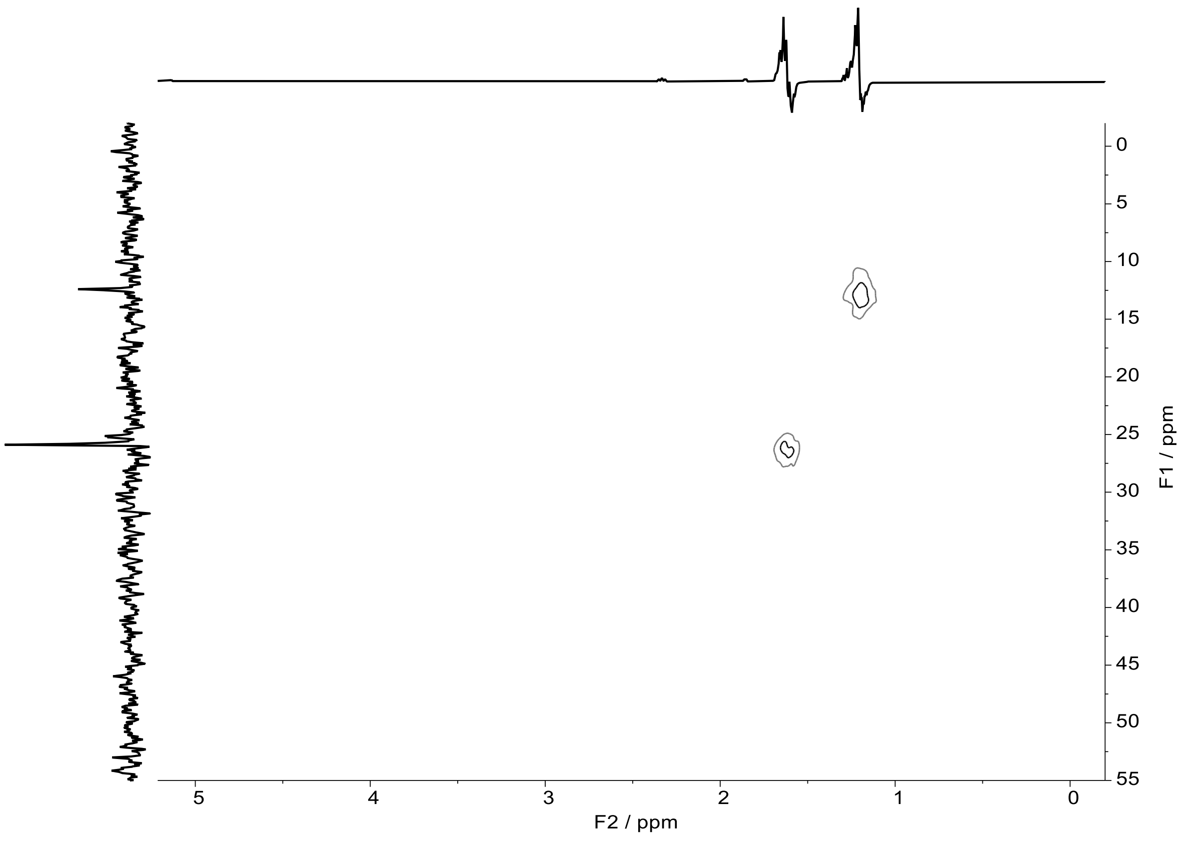

Figure S77. *Gas-phase* ^{13}C-^{1}H *HMQC of the PHIP hydrogenation of 1-butene to butane using catalyst* ***1*** *(298 K).*

Representative Spectra: Propyne

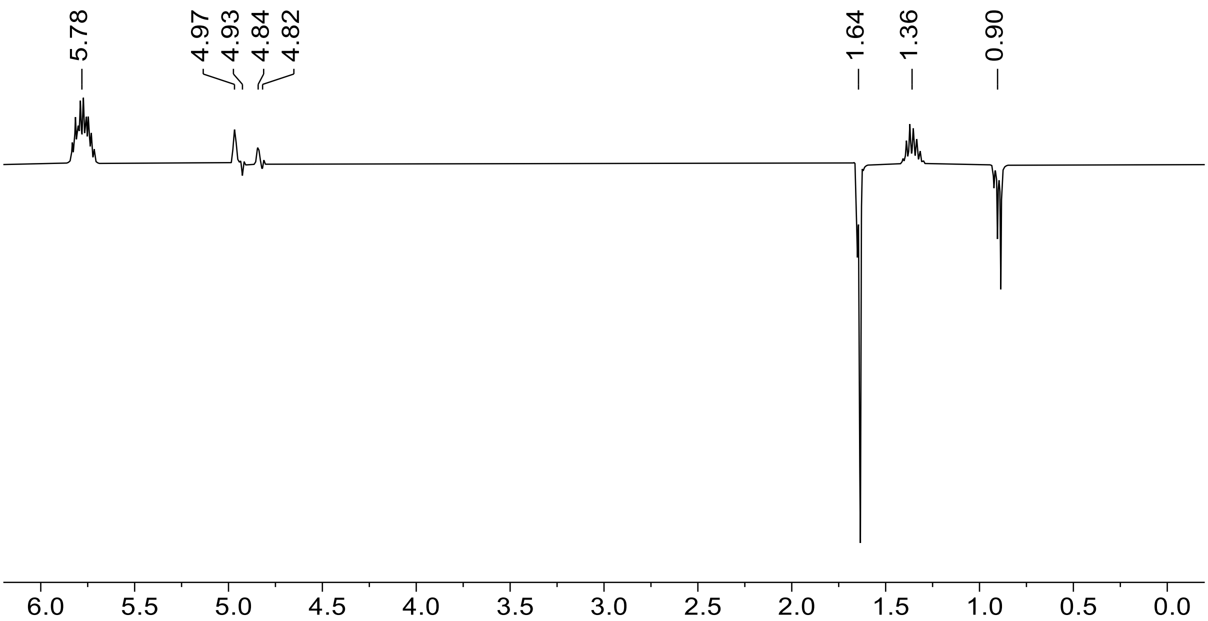

Figure S78. *Gas-phase ¹H NMR spectrum of the PHIP hydrogenation of propyne to propene and propane using catalyst **1** under mostly ALTADENA conditions (298 K).*

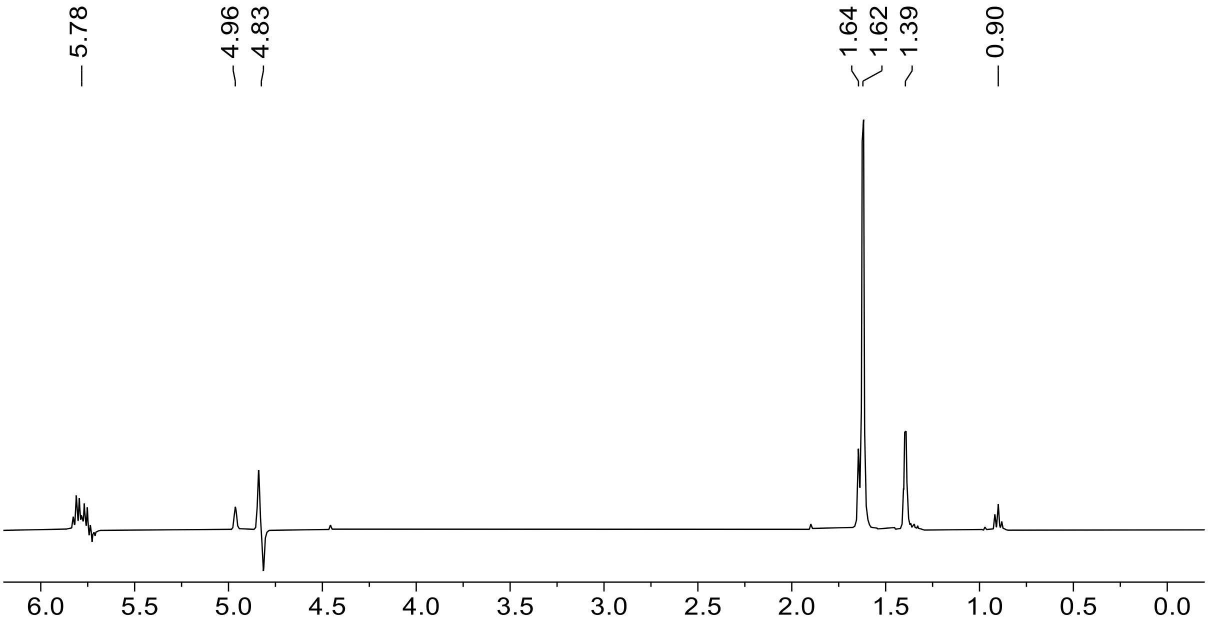

Figure S79. *Gas-phase ¹H NMR spectrum of the PHIP hydrogenation of propyne to propene and propane catalyst **1** under mostly PASADENA conditions (approximately 30 seconds after insertion into the spectrometer, 298 K).*

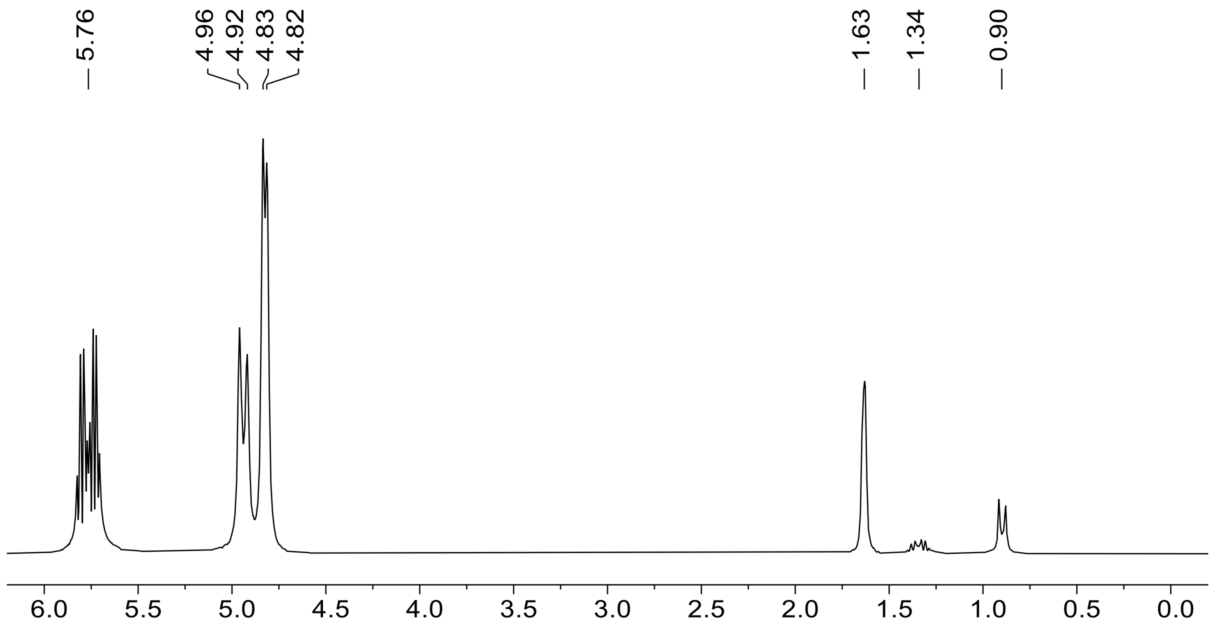

Figure S80. *Gas-phase OPSYdq spectrum of the PHIP hydrogenation of propyne to propene and propane using catalyst **1** under mostly ALTADENA conditions. The spectrum has been magnitude calculated to demonstrate all components in phase (298 K).*

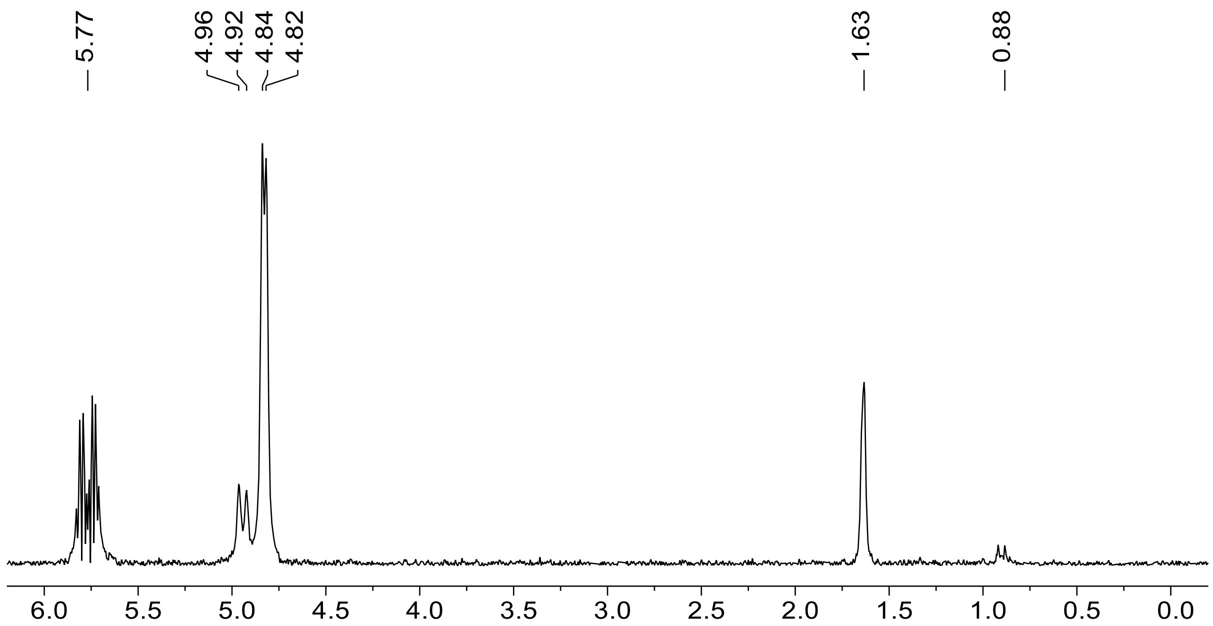

Figure S81. *Gas-phase OPSYdq spectrum of the PHIP hydrogenation of propyne to propene and propane using catalyst **1** under mostly PASADENA conditions. The spectrum has been magnitude calculated to demonstrate all components in phase (298 K).*

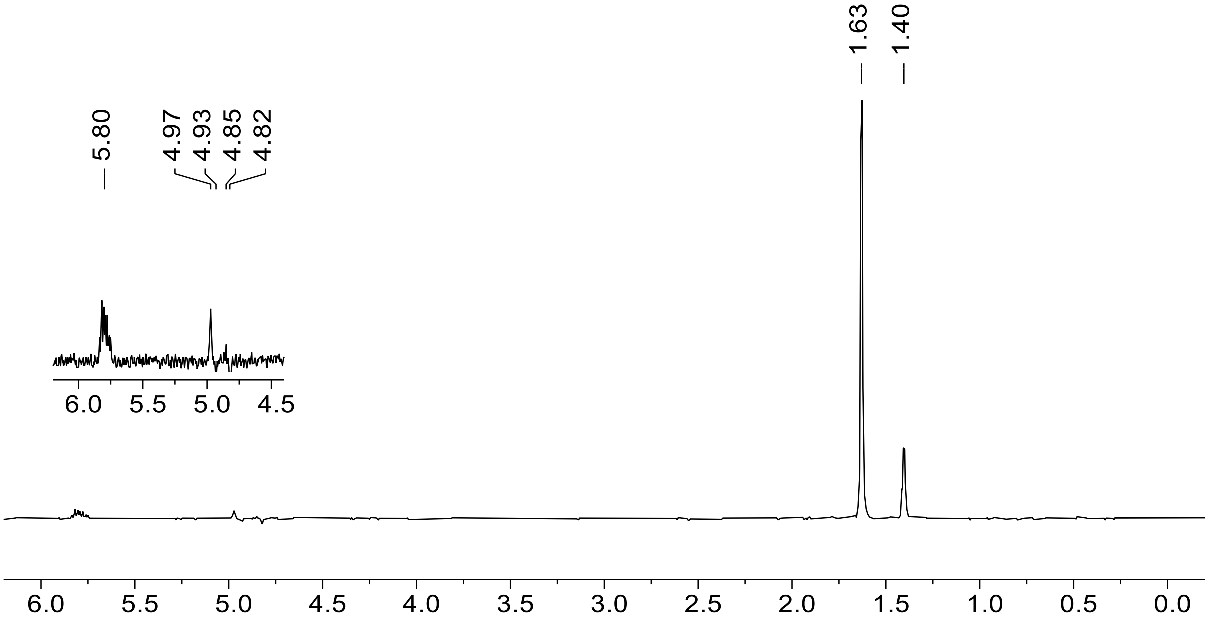

Figure S82. *Gas-phase* ^{1}H *NMR spectrum of the PHIP hydrogenation of propyne to propene and propane using catalyst* ***1*** *on a second substrate charge under mostly PASADENA conditions. The inset region is magnified approximately 10-fold to demonstrate weakly enhanced propene resonances (298 K).*

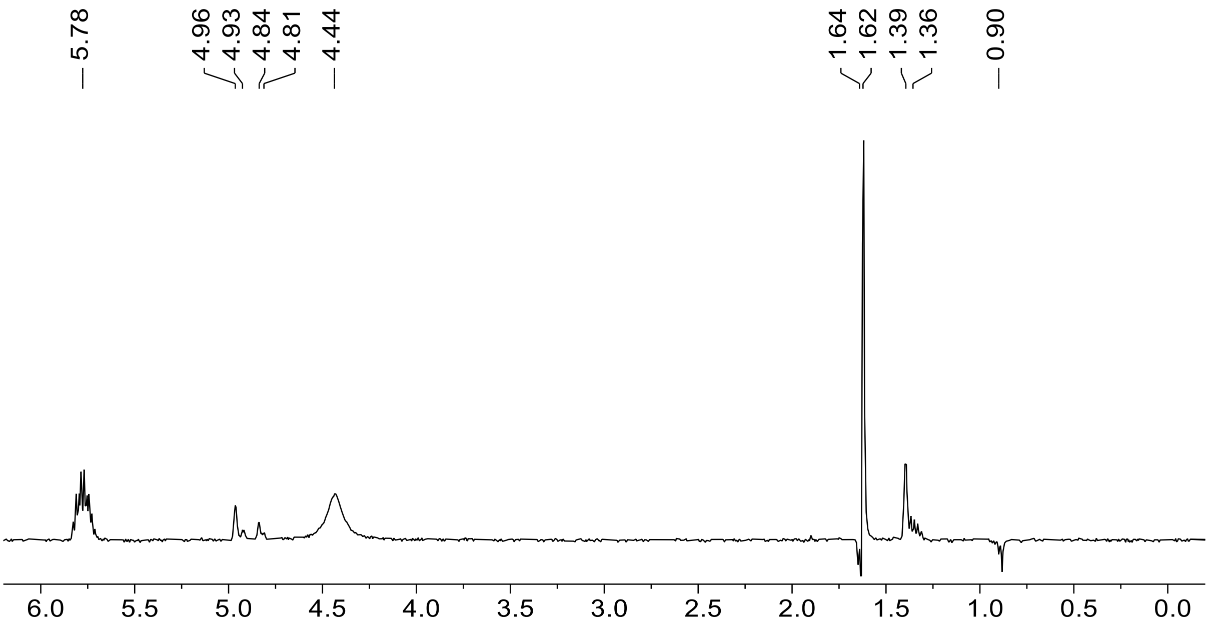

Figure S83. *Gas-phase* ^{1}H *NMR spectrum of the hydrogenation of propyne to propene and propane recorded immediately after insertion into the NMR spectrometer using catalyst* ***1*** *and thermally polarised hydrogen (298 K).*

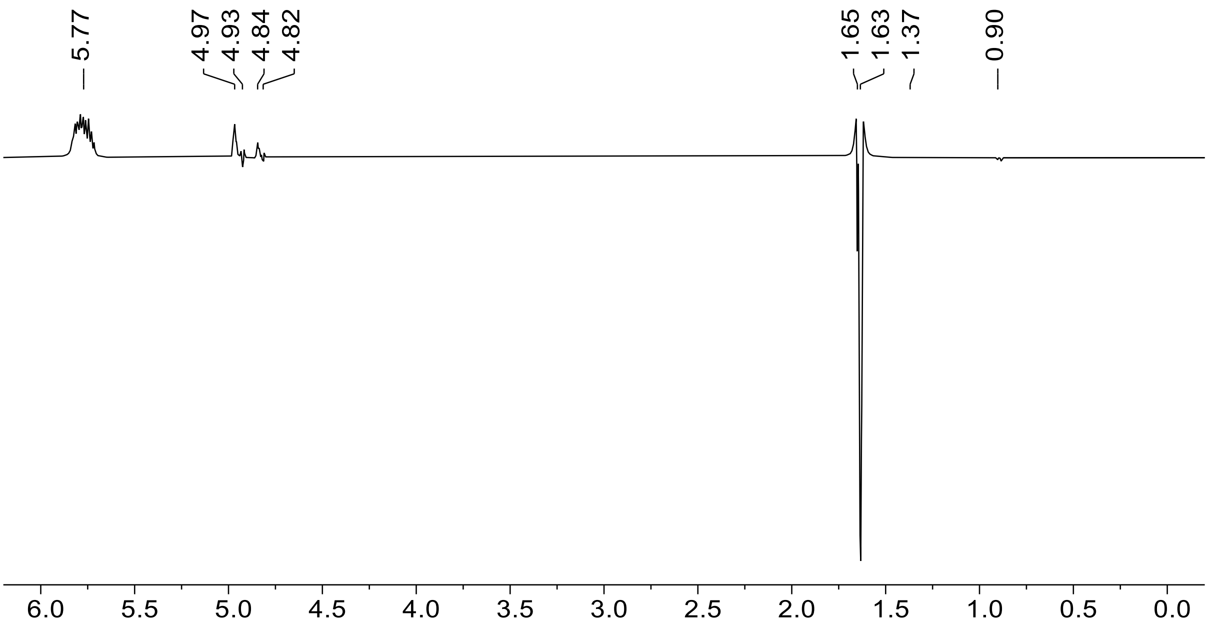

Figure S84. *Gas-phase* ^{1}*H NMR spectrum of the PHIP hydrogenation of propyne to propene and propane using catalyst* ***4*** *using catalyst* ***7*** *under mostly ALTADENA conditions (298 K).*

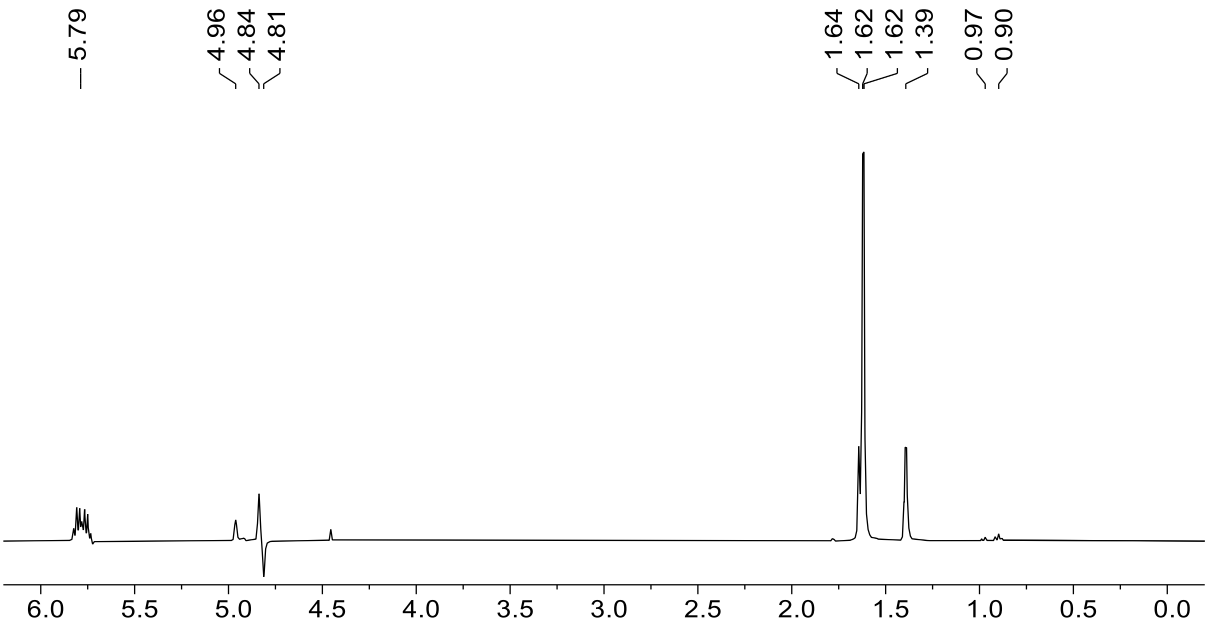

Figure S85. *Gas-phase* ^{1}*H NMR spectrum of the PHIP hydrogenation of propyne to propene and propane using catalyst* ***4*** *under mostly PASADENA conditions (approximately 30 seconds after insertion into the spectrometer) (298 K).*

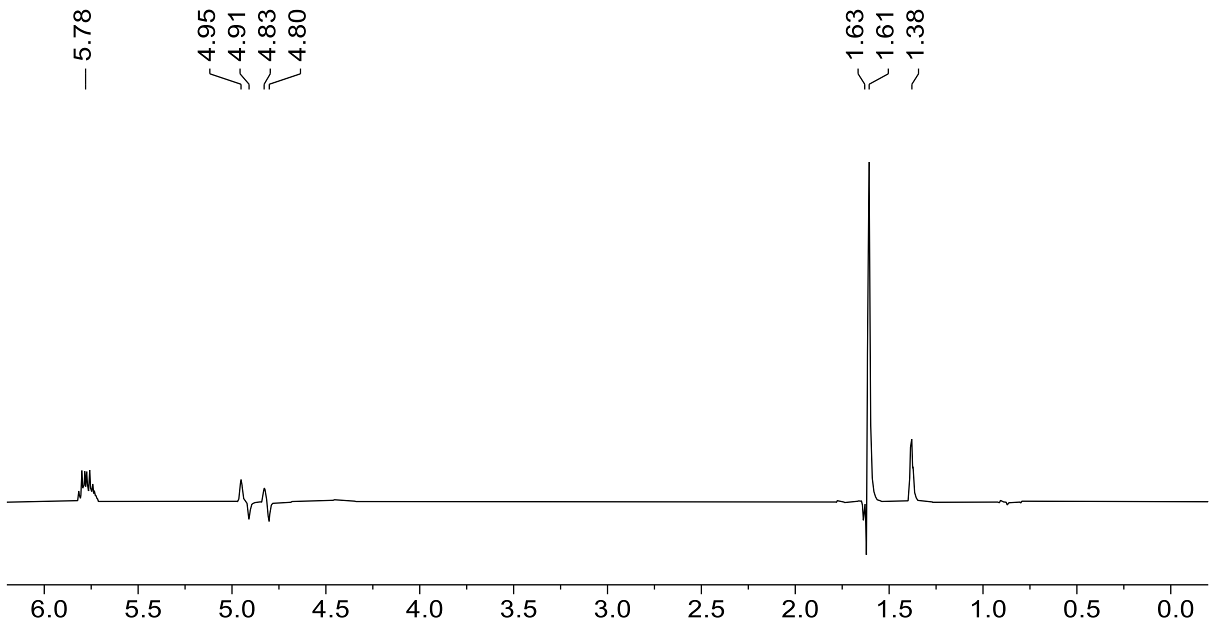

Figure S86. *Gas-phase ^1H NMR spectrum of the PHIP hydrogenation of propyne to propene and propane using catalyst **7** under mostly ALTADENA conditions (298 K).*

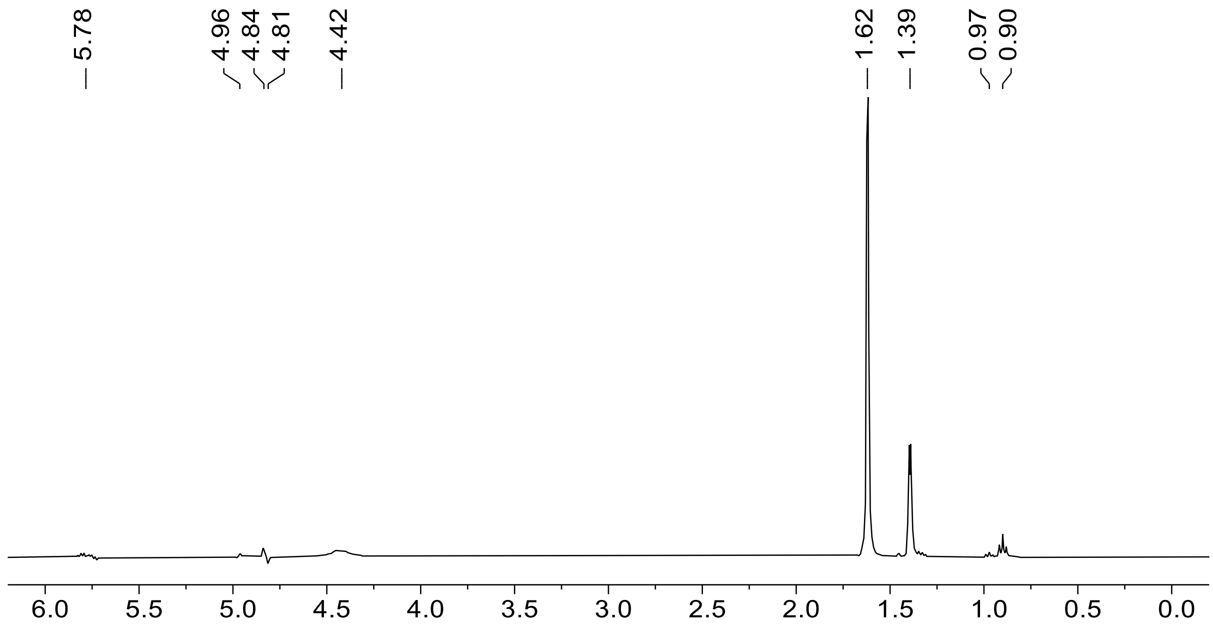

Figure S87. *Gas-phase ^1H NMR spectrum of the PHIP hydrogenation of propyne to propene and propane using catalyst **7** under mostly PASADENA conditions (approximately 30 seconds after insertion into the spectrometer) (298 K).*

Representative Spectra: 1-Butyne

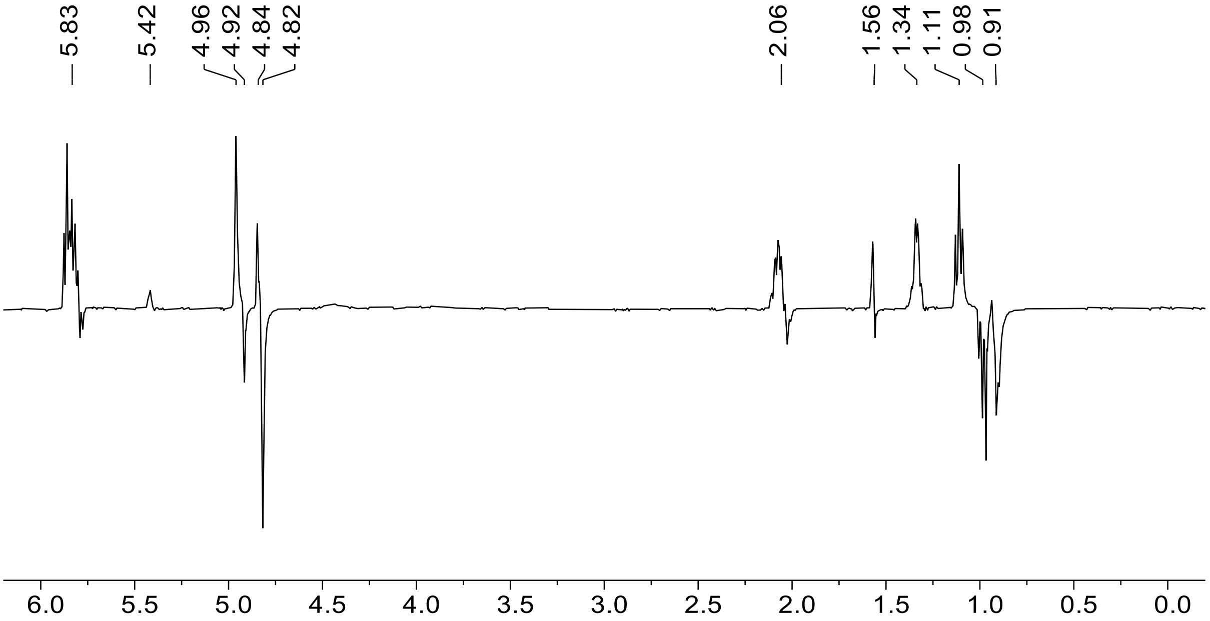

Figure S88. *Gas-phase* [1]*H NMR spectrum of the PHIP hydrogenation of 1-butyne to 1-butene and butane using catalyst* ***1*** *under mostly ALTADENA conditions (298 K).*

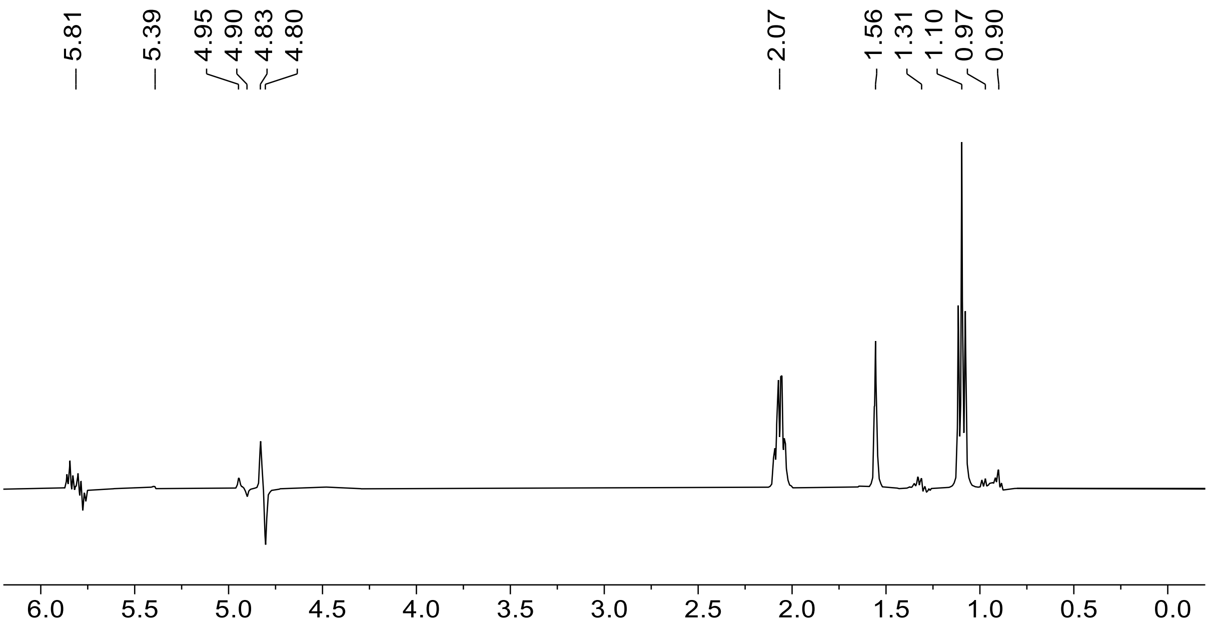

Figure S89. *Gas-phase* [1]*H NMR spectrum of the PHIP hydrogenation of 1-butyne to 1-butene and butane using catalyst* ***1*** *under mostly PASADENA conditions (approximately 30 seconds after insertion into the spectrometer) (298 K).*

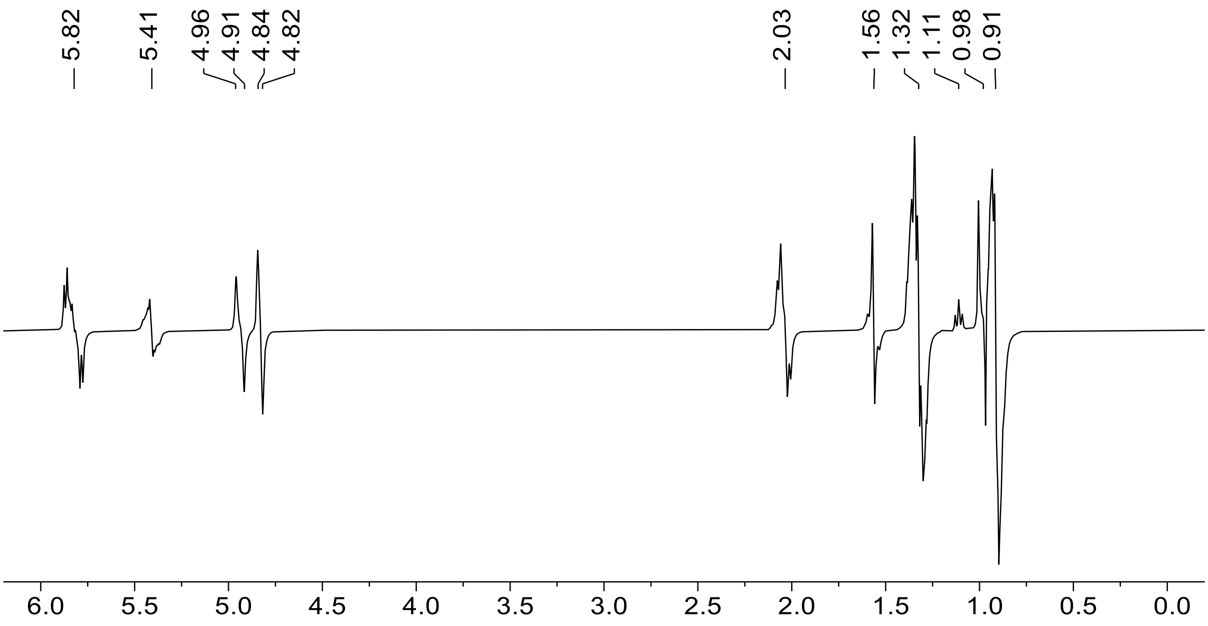

Figure S90. *Gas-phase ^{1}H NMR spectrum of the PHIP hydrogenation of 1-butyne to 1-butene and butane using catalyst **1** under PASADENA conditions (approximately 165 seconds after insertion into the spectrometer) (298 K).*

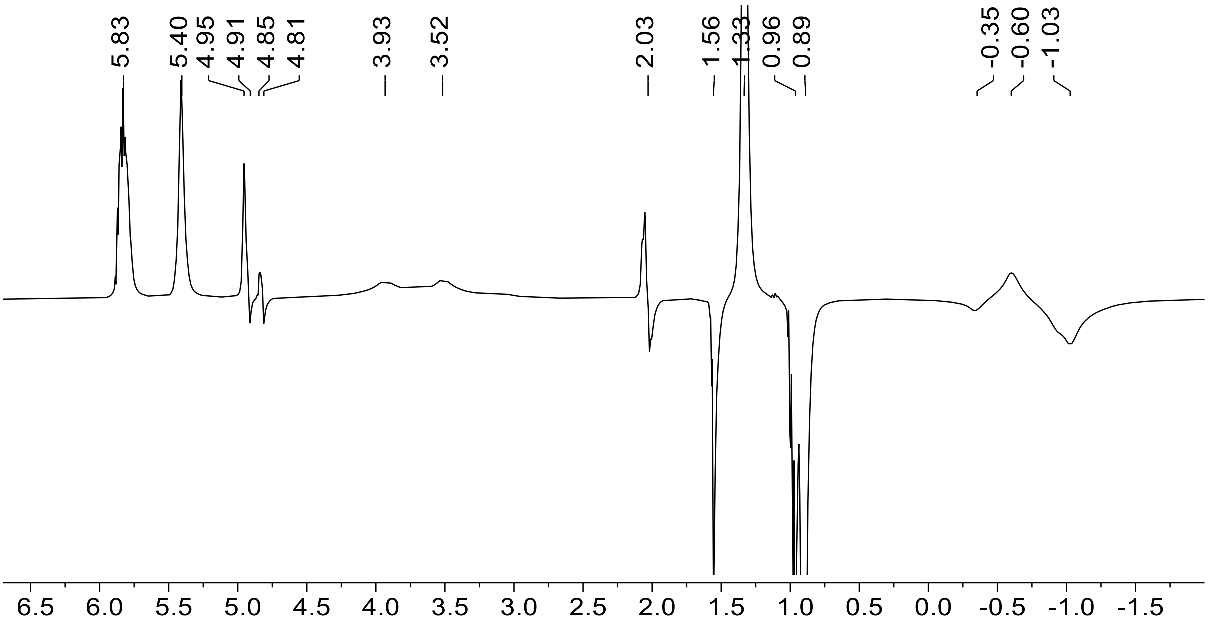

Figure S91. *Gas-phase ^{1}H NMR spectrum of the PHIP hydrogenation of 1-butyne to 1-butene and butane using catalyst **1** under ALTADENA conditions with increased scaling to demonstrate broad signals tentatively assigned as a PHIP enhanced σ-complex (298 K).*

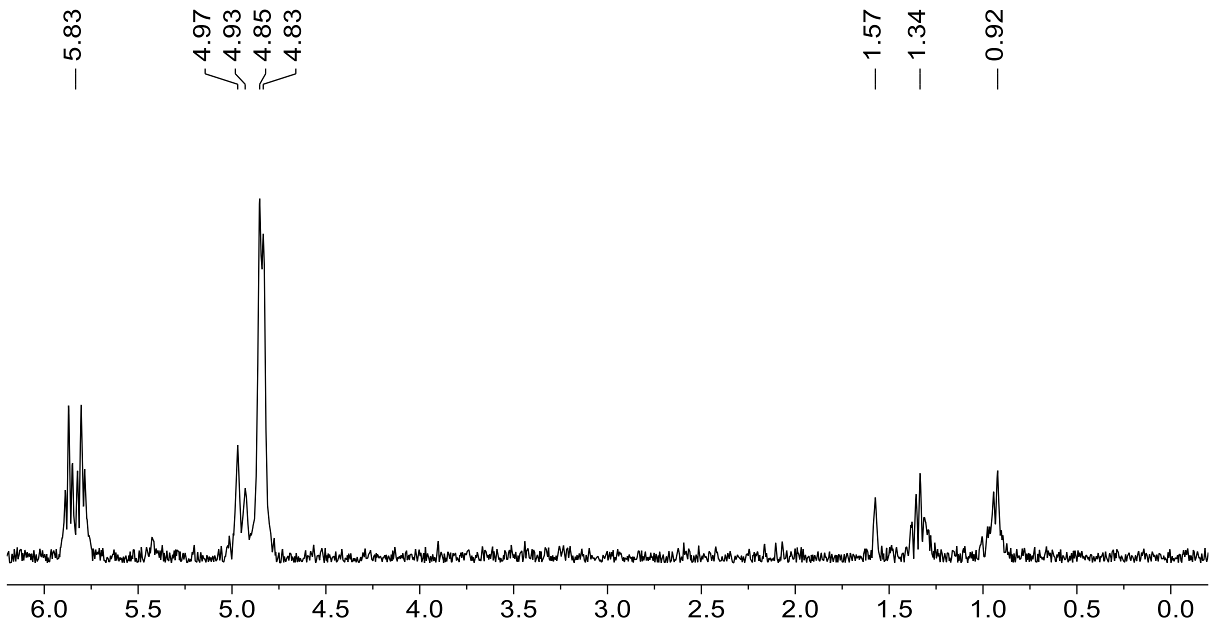

Figure S92. *Gas-phase OPSYdq spectrum of the PHIP hydrogenation of 1-butyne to 1-butene and butane using catalyst **1** under mostly ALTADENA conditions. The spectrum has been magnitude calculated to demonstrate all components in phase (298 K).*

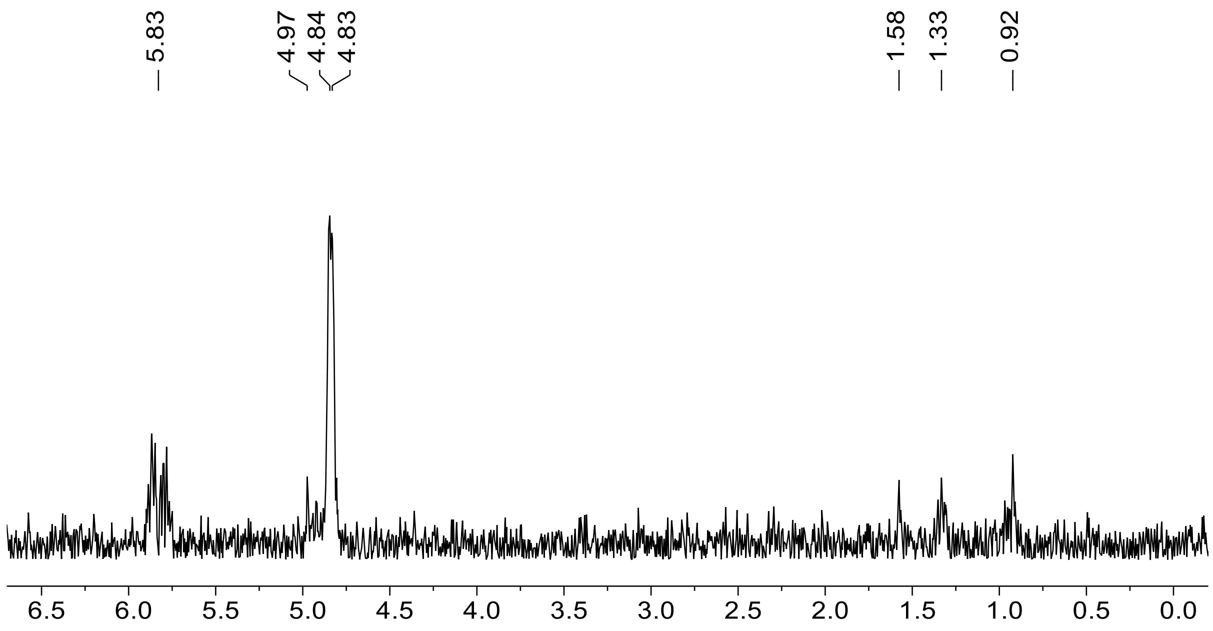

Figure S93. *Gas-phase OPSYdq spectrum of the PHIP hydrogenation of 1-butyne to 1-butene and butane using catalyst **1** under PASADENA conditions (approximately 30 seconds after insertion into the spectrometer). The spectrum has been magnitude calculated to demonstrate all components in phase (298 K).*

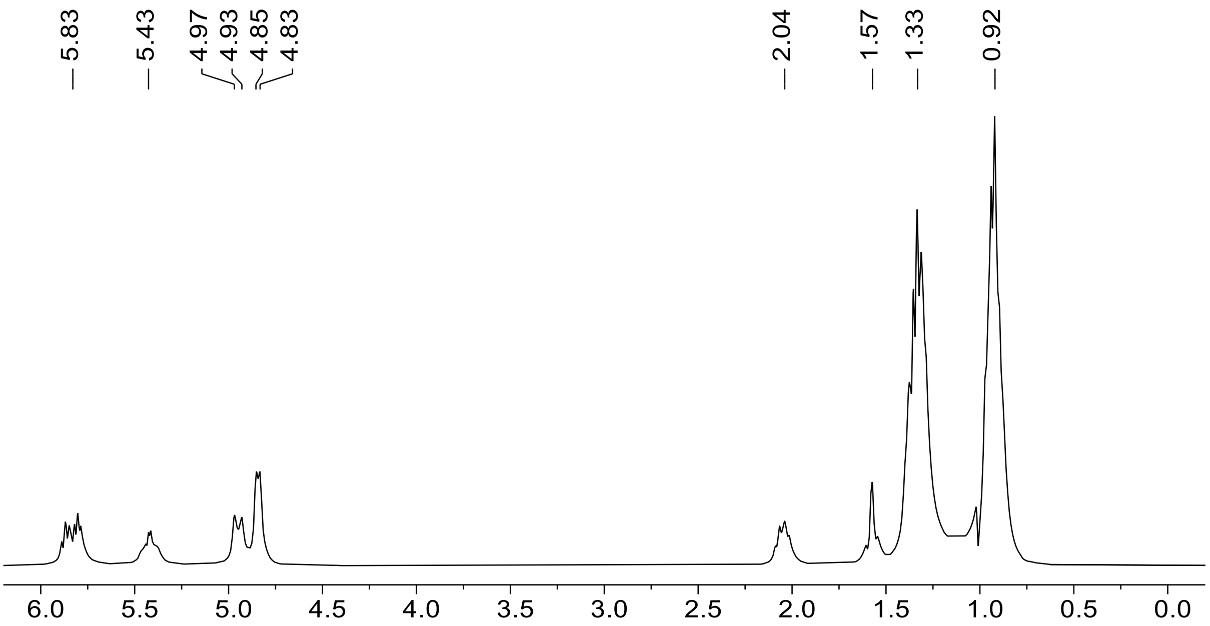

Figure S94. *Gas-phase OPSYdq spectrum of the PHIP hydrogenation of 1-butyne to 1-butene and butane using catalyst **1** under PASADENA conditions (approximately 230 seconds after insertion into the spectrometer). The spectrum has been magnitude calculated to demonstrate all components in phase (298 K).*

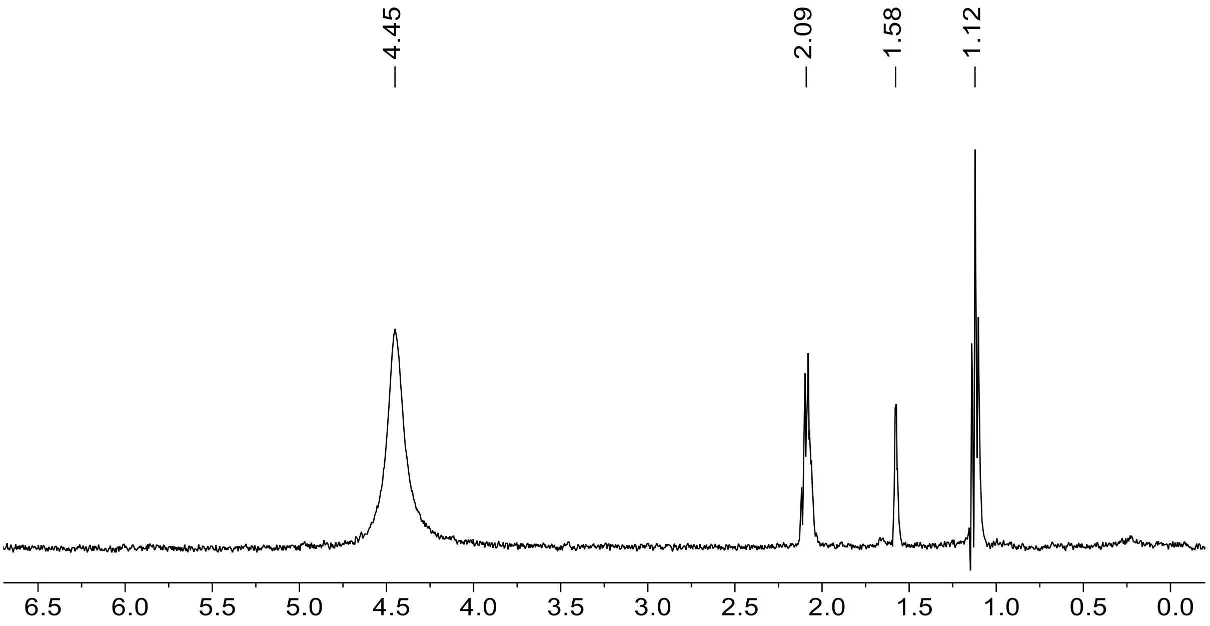

Figure S95. *Gas-phase ^{1}H NMR spectrum of the hydrogenation of 1-butyne to 1-butene and butane recorded immediately after insertion into the NMR spectrometer using catalyst **1** and thermally polarised hydrogen (298 K).*

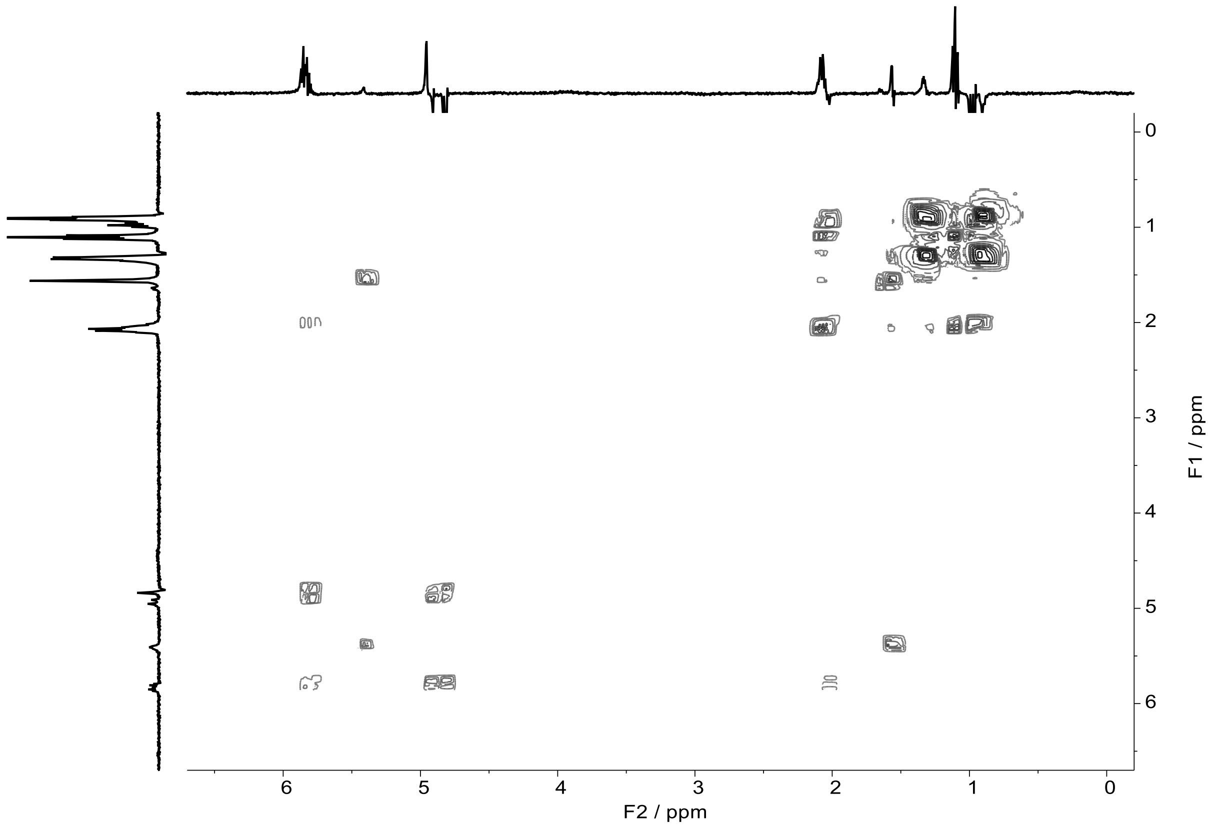

Figure S96. *Gas-phase* [1]*H-*[1]*H COSY of the PHIP hydrogenation of 1-butyne to 1-butene and butane using catalyst* ***1*** *(298 K).*

Representative Spectra: 1-Butene/Propene Mixtures

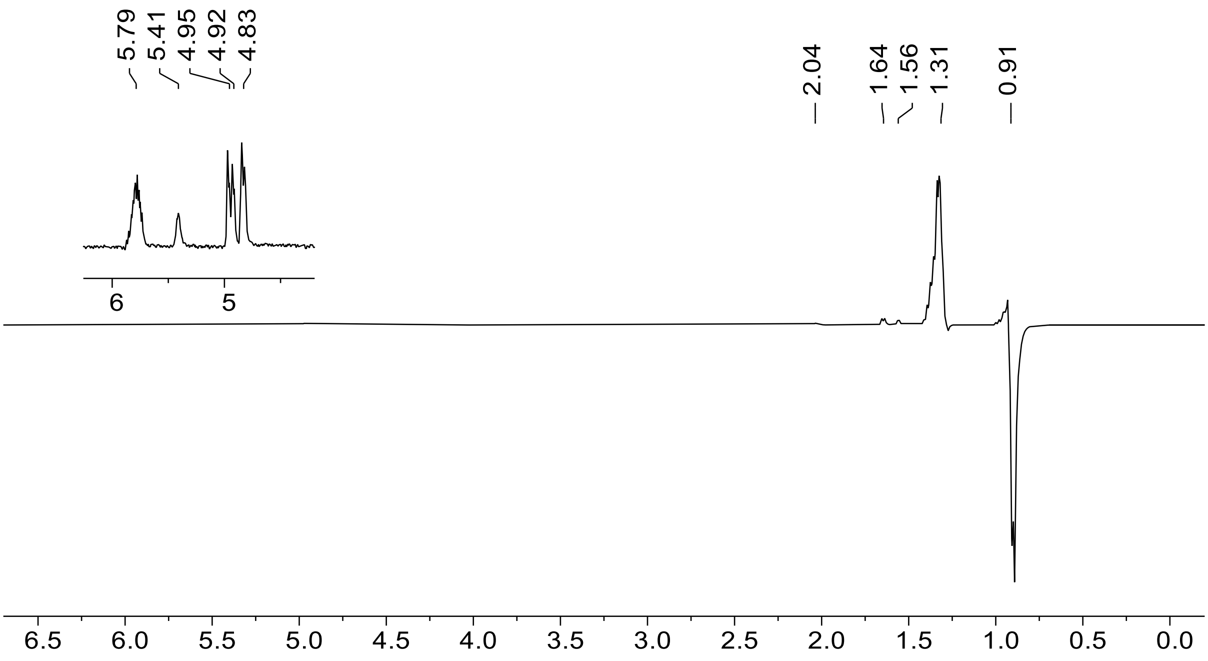

Figure S97. *Gas-phase* ^{1}H *NMR spectrum of the PHIP hydrogenation of a mixture of 1-butene and propene using catalyst* ***1*** *under mostly ALTADENA conditions. The inset region is magnified approximately 80-fold to demonstrate the alkene resonances (298 K).*

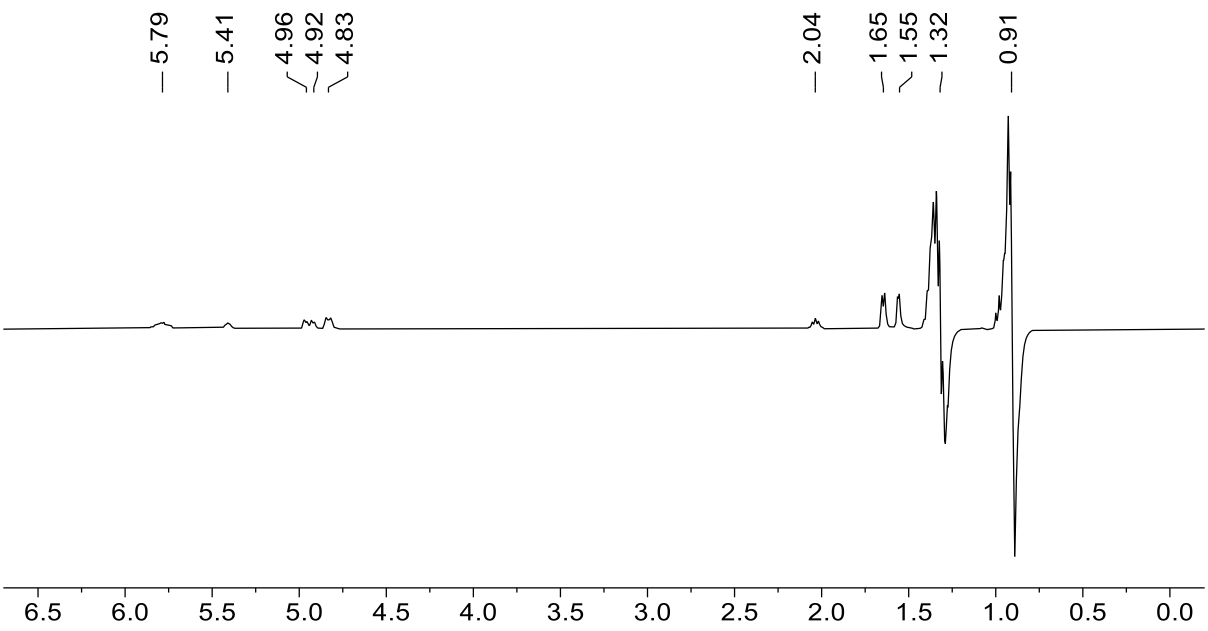

Figure S98. *Gas-phase* ^{1}H *NMR spectrum of the PHIP hydrogenation of a mixture of 1-butene and propene using catalyst* ***1*** *under mostly PASADENA (approximately 30 seconds after insertion into the spectrometer) conditions (298 K).*

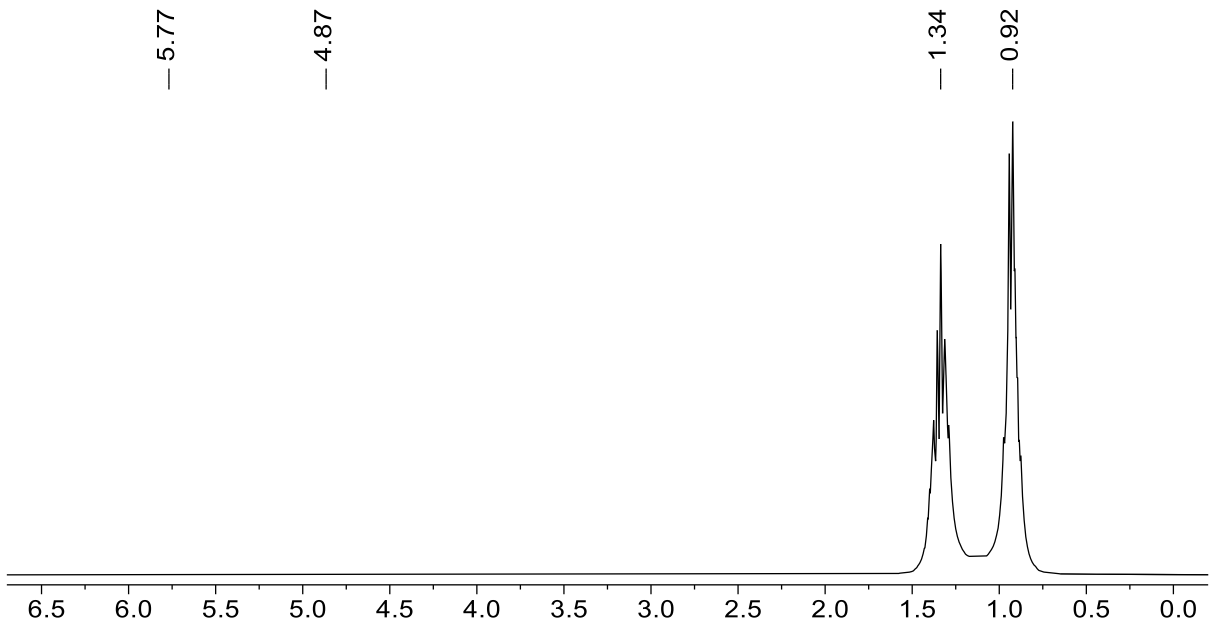

Figure S99. *Gas-phase OPSYdq spectrum of the PHIP hydrogenation of 1-butene and propene using catalyst* ***1****. The spectrum has been magnitude calculated to demonstrate all components in phase. (298 K).*

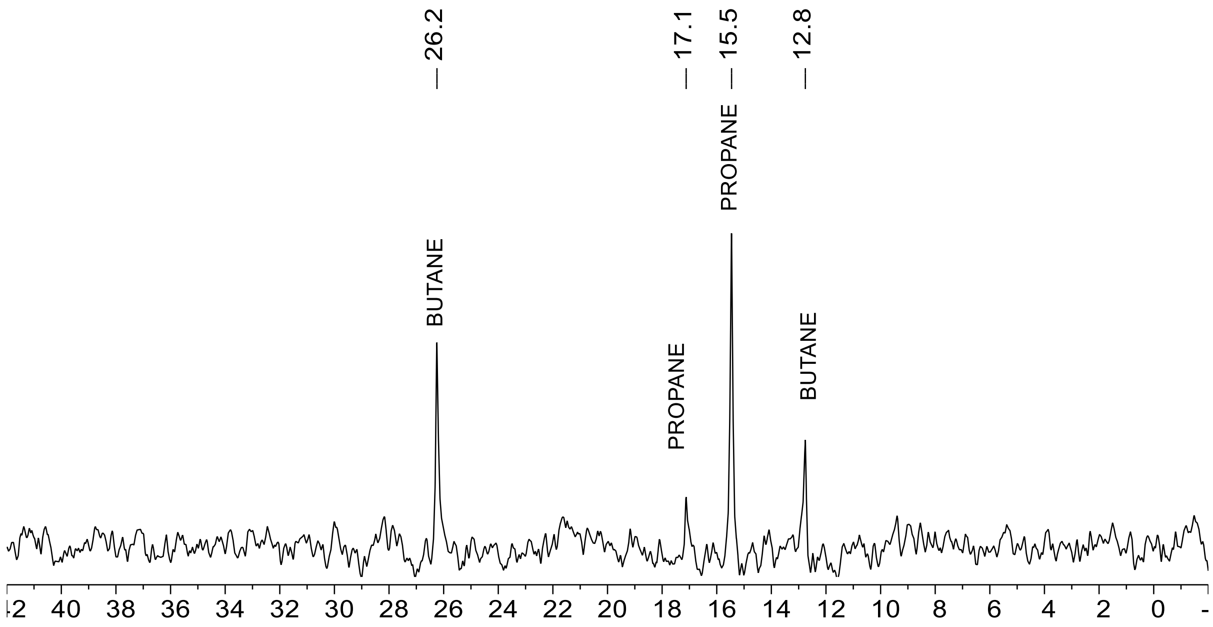

Figure S100. *Gas-phase $^{13}C\{^{1}H\}$ INEPT of the PHIP hydrogenation of a mixture of 1-butene and propene using catalyst* ***1*** *under mostly ALTADENA conditions (298 K).*

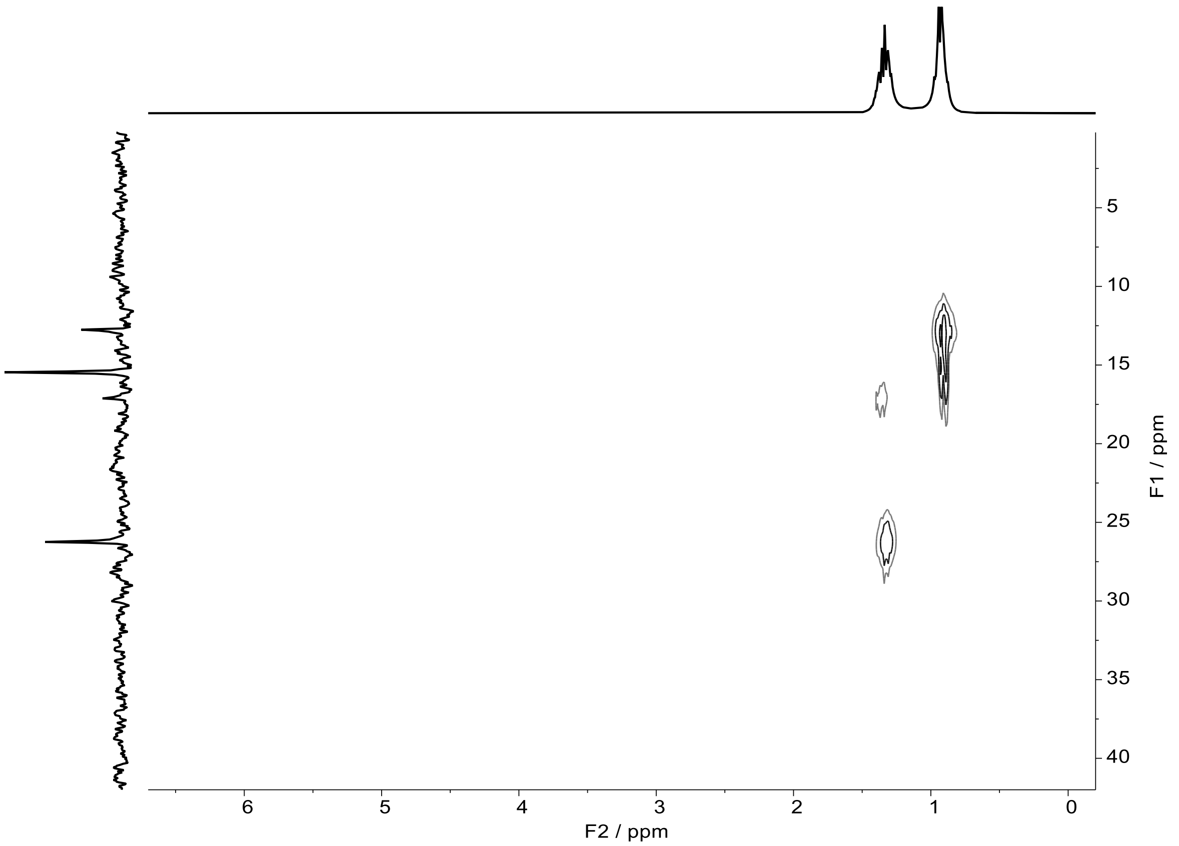

Figure S101. *Gas-phase* ^{13}C*-*^{1}H *HMQC of the PHIP hydrogenation of a mixture of 1-butene and propene using catalyst* ***1*** *(298 K).*

Single Crystal X-Ray Diffraction

Single-crystal X-ray diffraction data were collected (ω-scans) on a Rigaku SuperNova diffractometers with Cu-K_α (λ = 1.54184 Å) radiation equipped with an N_2 gas Oxford Cryosystems Cryostream unit. Diffraction images from raw frame data were reduced using CrysAlis Pro. The structures were solved using SHELXT[15] and refined to convergence on F^2 and against all independent reflections by full-matrix least-squares using SHELXL[16] in combination with the Olex2 GUI.[17] All non-hydrogen atoms were refined anisotropically; hydrogen atoms were geometrically placed unless otherwise stated and allowed to ride on their parent atoms. The CF_3 groups on the [BAr^F_4] anions were disordered and modelled over two domains and restrained to maintain sensible geometries. Distances and angles were calculated using the full covariance matrix.

	**1**
**Empirical Formula**	$C_{54}H_{60}BCl_2F_{24}P_2Rh$
**Formula Weight**	1411.58
**Temperature/K**	110.0(10)
**Crystal System**	Tetragonal
**Space group**	$P4_12_12$
**a/Å**	13.15540(10)
**b/Å**	13.15540(10)
**c/Å**	69.8799(5)
**α/°**	90
**β/°**	90
**γ/°**	90
**Volume/Å³**	12093.7(2)
**Z**	8
**ρ_{calc} g/cm³**	1.551
**μ/mm⁻¹**	4.593
**F(000)**	5712.0
**Crystal size/mm³**	0.219 × 0.157 × 0.1
**Radiation**	Cu-Kα (λ = 1.54184)
**2Θ range for data collection/°**	6.838 to 133.182
**Index ranges**	-15 ≤ h ≤ 15, -11 ≤ k ≤ 15, -83 ≤ l ≤ 83
**Reflections collected**	105812
**Independent reflections**	10698 [R_{int} = 0.0712, R_{sigma} = 0.0378]
**Data/restraints/parameters**	10698/1004/966
**Goodness-of-fit on F²**	1.171
**Final R index [I>=2σ (I)]**	R_1 = 0.0889, wR_2 = 0.1959
**Final R index [all data]**	R_1 = 0.0909, wR_2 = 0.1970
**Largest diff. peak/hole / e Å⁻³**	1.01/-0.94
**CCDC Deposition Number**	2222279

Table S4. *Selected crystallographic data.*

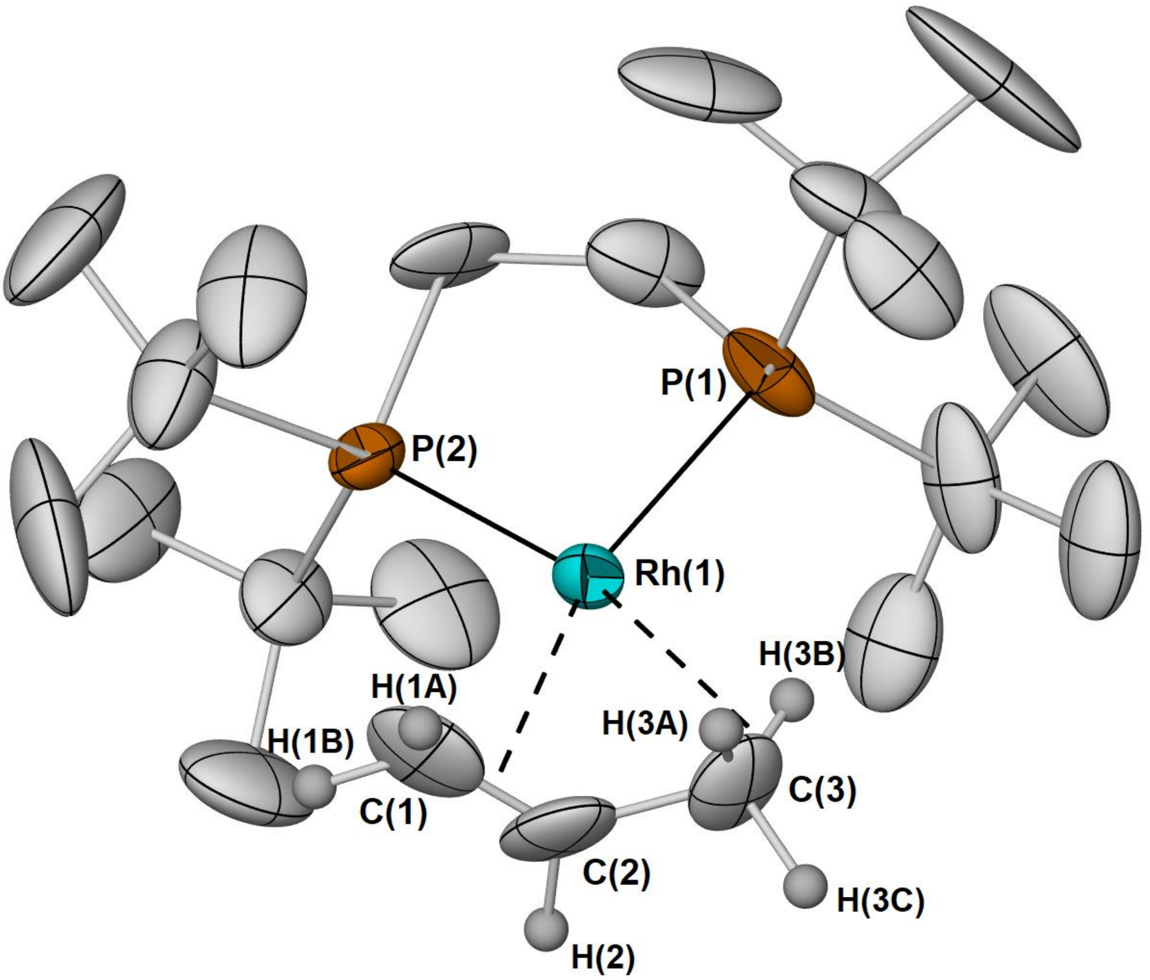

Figure S102. *The molecular structure of [Rh(^{t}Bu$_2$PCH$_2$CH$_2$P^{t}Bu$_2$)(nbd)][BAr^F$_4$]* ***1*** *as determined by single crystal X-ray diffraction from material recrystallised from CH$_2$Cl$_2$/hexane.*

References

(1) Pangborn, A. B.; Giardello, M. A.; Grubbs, R. H.; Rosen, R. K.; Timmers, F. J. Safe and Convenient Procedure for Solvent Purification. *Organometallics* **1996**, *15* (5), 1518–1520. DOI: 10.1021/om9503712.

(2) Furfari, S. K.; Tegner, B. E.; Burnage, A. L.; Doyle, L. R.; Bukvic, A. J.; Macgregor, S. A.; Weller, A. S. Selectivity of Rh···H−C Binding in a Σ-Alkane Complex Controlled by the Secondary Microenvironment in the Solid State. *Chem. Eur. J.* **2021**, *27* (9), 3177–3183. DOI: 10.1002/chem.202004585.

(3) Doyle, L. R.; Heath, A.; Low, C. H.; Ashley, A. E. A Convenient Synthetic Protocol to 1,2-Bis(Dialkylphosphino)Ethanes. *Adv. Synth. Catal.* **2014**, *356* (2–3), 603–608. DOI: 10.1002/adsc.201300787.

(4) Chadwick, F. M.; McKay, A. I.; Martinez-Martinez, A. J.; Rees, N. H.; Krämer, T.; Macgregor, S. A.; Weller, A. S. Solid-State Molecular Organometallic Chemistry. Single-Crystal to Single-Crystal Reactivity and Catalysis with Light Hydrocarbon Substrates. *Chem. Sci.* **2017**, *8* (9), 6014–6029. DOI: 10.1039/C7SC01491K.

(5) Fulmer, G. R.; Miller, A. J. M.; Sherden, N. H.; Gottlieb, H. E.; Nudelman, A.; Stoltz, B. M.; Bercaw, J. E.; Goldberg, K. I. NMR Chemical Shifts of Trace Impurities: Common Laboratory Solvents, Organics, and Gases in Deuterated Solvents Relevant to the Organometallic Chemist. *Organometallics* **2010**, *29* (9), 2176–2179. DOI: 10.1021/om100106e.

(6) Aguilar, J. A.; Elliott, P. I. P.; López-Serrano, J.; Adams, R. W.; Duckett, S. B. Only Para-Hydrogen Spectroscopy (OPSY), a Technique for the Selective Observation of Para-Hydrogen Enhanced NMR Signals. *Chem. Commun.* **2007**, No. 11, 1183–1185. DOI: 10.1039/B616307F.

(7) Morcombe, C. R.; Zilm, K. W. Chemical Shift Referencing in MAS Solid State NMR. *J. Magn. Reson.* **2003**, *162* (2), 479–486. DOI: 10.1016/S1090-7807(03)00082-X.

(8) Hu, B.; Gay, I. D. Probing Surface Acidity by 31 P Nuclear Magnetic Resonance Spectroscopy of Arylphosphines. *Langmuir* **1999**, *15* (2), 477–481. DOI: 10.1021/la980750a.

(9) Martínez-Martínez, A. J.; Royle, C. G.; Furfari, S. K.; Suriye, K.; Weller, A. S. Solid–State Molecular Organometallic Catalysis in Gas/Solid Flow (Flow-SMOM) as Demonstrated by Efficient Room Temperature and Pressure 1-Butene Isomerization. *ACS Catal.* **2020**, *10* (3), 1984–1992. DOI: 10.1021/acscatal.9b03727.

(10) Richardson, P. M.; John, R. O.; Parrott, A. J.; Rayner, P. J.; Iali, W.; Nordon, A.; Halse, M. E.; Duckett, S. B. Quantification of Hyperpolarisation Efficiency in SABRE and SABRE-Relay Enhanced NMR Spectroscopy. *Phys. Chem. Chem. Phys.* **2018**, *20* (41), 26362–26371. DOI: 10.1039/C8CP05473H.

(11) Blazina, D.; Duckett, S. B.; Halstead, T. K.; Kozak, C. M.; Taylor, R. J. K.; Anwar, M. S.; Jones, J. A.; Carteret, H. A. Generation and Interrogation of a Pure Nuclear Spin State by Parahydrogen-Enhanced NMR Spectroscopy: A Defined Initial State for Quantum Computation. *Magn. Reson. Chem.* **2005**, *43* (3), 200–208. DOI: 10.1002/mrc.1540.

(12) Newville, M. IFEFFIT : Interactive XAFS Analysis and FEFF Fitting. *J. Synchrotron Radiat.* **2001**, *8* (2), 322–324. DOI: 10.1107/S0909049500016964.

(13) Ravel, B.; Newville, M. ATHENA , ARTEMIS , HEPHAESTUS : Data Analysis for X-Ray Absorption Spectroscopy Using IFEFFIT. *J. Synchrotron Radiat.* **2005**, *12* (4), 537–541. DOI: 10.1107/S0909049505012719.

(14) Lubben, A. T.; McIndoe, J. S.; Weller, A. S. Coupling an Electrospray Ionization Mass Spectrometer with a Glovebox: A Straightforward, Powerful, and Convenient Combination for Analysis of Air-Sensitive Organometallics. *Organometallics* **2008**, *27* (13), 3303–3306. DOI: 10.1021/om800164e.

(15) Sheldrick, G. M. SHELXT – Integrated Space-Group and Crystal-Structure Determination. *Acta Crystallogr. Sect. A Found. Adv.* **2015**, *71* (1), 3–8. DOI: 10.1107/S2053273314026370.

(16) Sheldrick, G. M. Crystal Structure Refinement with SHELXL. *Acta Crystallogr. Sect. C Struct. Chem.* **2015**, *71* (1), 3–8. DOI: 10.1107/S2053229614024218.

(17) Dolomanov, O. V.; Bourhis, L. J.; Gildea, R. J.; Howard, J. A. K.; Puschmann, H. OLEX2 : A Complete Structure Solution, Refinement and Analysis Program. *J. Appl. Crystallogr.* **2009**, *42* (2), 339–341. DOI: 10.1107/S0021889808042726.
